# LMP1 enhances aerobic glycolysis in natural killer/T cell lymphoma

**DOI:** 10.1038/s41419-024-06999-7

**Published:** 2024-08-20

**Authors:** Wenting Song, Yuyang Gao, Jiazhuo Wu, Hongwen Li, Zhuangzhuang Shi, Chen Gong, Zihe Zhang, Zhaoming Li, Mingzhi Zhang

**Affiliations:** 1 Department of Oncology, The First Affiliated Hospital of Zhengzhou University, Zhengzhou, Henan China; 2 State Key Laboratory of Esophageal Cancer Prevention & Treatment and Henan Key Laboratory for Esophageal Cancer Research, The First Affiliated Hospital of Zhengzhou University, Zhengzhou, Henan China

**Keywords:** Cancer metabolism, T-cell lymphoma

## Abstract

Natural killer/T cell lymphoma (NKTCL) exhibits highly aggressive clinical behavior, and the outcomes for relapsed/refractory patients are still poor. Recently, the mechanism underlying the effect of Epstein-Barr virus (EBV) infection, which has not been fully defined in NKTCL, has attracted great attention. We explored how LMP1 promoted aerobic glycolysis via metabolic sequencing combined with mRNA sequencing and immunoprecipitation coupled to mass spectrometry. Experimental assays were used to determine the effects of LMP1 and its downstream pathway on the function and glucose metabolism of NKTCL cells. The correlations between LMP1 expression in patients and their clinical features, treatment response, and prognosis were analyzed. Results show that LMP1 enhances NKTCL cell proliferation in vitro and in vivo, inhibits apoptosis, and decreases gemcitabine sensitivity. In addition, LMP1 also enhances aerobic glycolysis in NKTCL cells, as indicated by increases in glucose uptake, lactate production, and extracellular acidification rate. Clinically, LMP1 expression is correlated with risk stratification, treatment response, and prognosis, and higher LMP1 expression indicates greater SUVmax for NKTCL patients. Mechanistically, LMP1 competitively binds to TRAF3 to promote cell proliferation and aerobic glycolysis by regulating the noncanonical NF-κB pathway. The application of an NF-κB pathway inhibitor or reactivation of the NF-κB pathway affects aerobic glycolysis and the biological function of NKTCL cells. In summary, this study is the first to describe and define in detail how LMP1 affects glucose metabolism in NKTCL and might provide a novel perspective for further treatment.

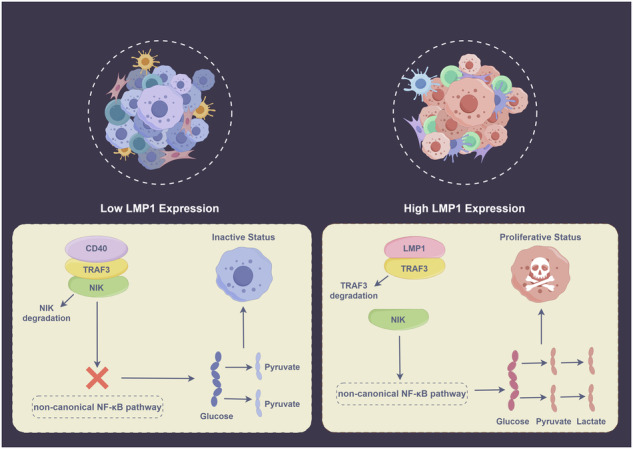

## Introduction

Natural killer/T cell lymphoma (NKTCL), a subtype of T cell and NK-cell lymphoid proliferations and lymphomas classified by the latest World Health Organization (WHO) [1], presents highly aggressive clinical behavior and is particularly prevalent in Asian and South American populations [2]. In recent decades, therapeutic regimens containing L-asparaginase, including DDGP [3], modified SMILE [4], and P-GemOx [5], have been recommended by the National Comprehensive Cancer Network guidelines based on improved clinical prognosis [6]. However, the outcomes for relapsed and refractory patients are still suboptimal [7]. Considering the close association of NKTCL with Epstein-Barr virus (EBV) infection [8], targeting EBV provides a promising strategy for future treatment. Latent membrane protein 1 (LMP1), encoded by EBV, promotes oncogenesis to facilitate malignant transformation in EBV-associated malignancies [9], and has been reported to play fundamental roles in nasopharyngeal carcinoma (NPC) [10, 11]. Thus, studies on the therapeutic potential of LMP1 for the treatment of NKTCL are urgently needed.

The reprogramming of metabolic pathways, regarded as a crucial hallmark of tumors, is associated with both tumorigenesis and disease progression [12]. Among them, the metabolism of glucose for aerobic glycolysis rather than oxidative phosphorylation, known as the Warburg effect, is an important metabolic feature of malignant lymphomas [13, 14], and can support infinite replication, self-sufficiency in growth signals, resistance to antigrowth signals, tumor invasion and metastasis, angiogenesis, and disturbance of the microenvironment [15]. It has been widely reported that viral infection is related to tumor pathogenesis and development, and the mechanism underlying the effect of viral infection on tumor metabolism has attracted great attention in recent years. Studies have shown that EBV and its encoded LMP1 can potentially alter glucose metabolism in NPC, through increasing the expression of vascular endothelial growth factor to stimulate angiogenesis [16], inhibiting glucose deprivation and metabolism to restore sensitivity to apoptosis induction [17], and decreasing monocyte migration and T cell activation to promote immune escape [18]. Hence, targeting LMP1 may represent a promising approach for treating EBV-positive malignancies by interfering with aerobic glycolysis. However, the effect of LMP1 on glucose metabolism in EBV-associated NKTCL and the detailed mechanism have not been fully defined.

In this study, we explored how LMP1 promoted aerobic glycolysis via metabolic sequencing combined with mRNA sequencing and immunoprecipitation (IP) coupled to mass spectrometry. Experimental assays determined the effects of LMP1 and its downstream pathway on the function and glucose metabolism of NKTCL cells. Clinically, the correlations between LMP1 expression in NKTCL patients and their clinical features, treatment response, prognosis, and the baseline maximum standardized uptake value (SUVmax) before treatment were analyzed. In summary, this study is the first to describe and define in detail the effect of LMP1 on glucose metabolism in NKTCL and might provide a novel perspective for further treatment.

## Materials and methods

### Patients and clinical data

A total of 58 formalin-fixed, paraffin-embedded tumor tissues from NKTCL patients were obtained from the First Affiliated Hospital of Zhengzhou University. All patients were reviewed and interpreted independently by three experienced pathologists, and diagnoses were made according to the latest WHO classification criteria. After diagnosis, enrolled patients received DDGP regimen, including gemcitabine, dexamethasone, cisplatin, and pegaspargase, as the first-line treatment. Immunohistochemistry (IHC) of LMP1 was performed according to standard procedures. The LMP1 antibody was listed in Additional File 1: Table S1. Staining was assessed according to the staining intensity and the positively stained area by a pathologist and verified by two other pathologists without prior knowledge of the patients’ information, and the specific evaluation criteria and definition of cut-off value were described in Additional File 2. The overall response rate (ORR) was defined as the proportion of patients who achieved a complete response (CR) or partial response (PR). Efficacy evaluation was conducted every 2 treatment cycles. Overall survival (OS) was defined as the interval from the date of first treatment to the date of death for any reason. Progression-free survival (PFS) was defined as the interval from the date of first treatment to the date of disease progression or death for any reason. The SUVmax was collected from the reports on pretreatment 18F-FDG PET-CT examinations. The clinical features of the patients and the correlations of these features with LMP1 expression were summarized in Table 1.

**Table 1:** Clinical features and their correlations with LMP1 expression of enrolled NKTCL patients.

Clinical features	*N*	LMP1 high expression	LMP1 low expression	*P*-value
Total	58	36	22	
**Gender**				0.7646
Male	46	29	17	
Female	12	7	5	
**Age (years)**				0.8444
>60	14	9	5	
≤60	44	27	17	
**“B” symptoms**				0.4722
Absent	32	18	14	
Present	26	18	8	
**CA staging**				0.5883
I–II	29	17	12	
III–IV	29	19	10	
**PINK-E score**				**0.0415**
0–1	22	10	12	
2–5	36	26	10	
**Serum LDH level**				0.07
Normal	23	11	12	
Elevated	35	25	10	
**Serum β2-MG level**				0.7368
Normal	30	18	12	
Elevated	28	18	10	
**Ki67 expression**				0.7646
å 50	46	29	17	
≤50	12	7	5	
**Bone marrow invasion**				0.6627
Absent	44	28	16	
Present	14	8	6	

*NKTCL* natural killer/T cell lymphoma, *N* number, *CA* the Chinese Southwest Oncology Group and Asia Lymphoma Study Group ENKTL, *PINK-E* Prognostic Index for Natural Killer cell lymphoma-Epstein-Barr virus, *LDH* lactate dehydrogenase, *β2-MG* β2-microglobulin.

The bold values include the main characteristics and the *P* value with statistical significance.

### Cell lines and culture

YT and NKYS cell lines were obtained from Dr. Wing C. Chan (City of Hope Medical Center), the SNT16 cell line was a gift from Guangzhou Bairui Biomedical Technology Company, Ltd. (China), and the SNK6 cell line was kindly provided by Dr. Norio Shimizu and Yu Zhang of Chiba University. The culture conditions were mentioned in Additional File 2.

### Construction of stable cell lines

Sequences for over-expression (OE)-LMP1 and its vector (OE-vector) were designed by Guangzhou Bairui Biomedical Technology Company, Ltd. (China). Sequences for short hairpin RNA (shRNA) of LMP1 (shLMP1) and negative control (shNC), and OE-RelB and its vector (OE-vector) were designed by Shanghai Genechem Company, Ltd. (China). Brief procedures were mentioned in Additional File 2.

### Cell proliferation assay

Cell proliferation assay was conducted with Cell Counting Kit-8 (CCK-8) reagent (UElandy, China). Brief procedures were mentioned in Additional File 2. Independent experiments were repeated at least three times.

### Cell apoptosis assay

Cell apoptosis assay was conducted with APC-Annexin V/PI Apoptosis Detection Kit (UElandy, China). Brief procedures were mentioned in Additional File 2. Independent experiments were repeated at least three times.

### Gemcitabine sensitivity assay

Gemcitabine sensitivity assay was conducted with CCK-8 reagent (UElandy, China). Brief procedures were mentioned in Additional File 2. Independent experiments were repeated at least three times.

### mRNA sequencing and metabolic sequencing analysis

Total RNA extraction, mRNA library construction and sequencing, sample preparation, LC–MS detection, and data analysis were performed by Suzhou PANOMIX Biomedical Tech Co., LTD. (China). Brief procedures were mentioned in Additional File 2.

### Droplet digital PCR (ddPCR)

RNA was extracted using RNAsimple Total RNA Kit (TIANGEN, China). cDNA was synthesized by UEIris RT mix with DNase (UElandy, China). The primers were synthesized by Hangzhou Shangyasai Biotechnology Co., Ltd. (China) and Sangon Biotech Co., Ltd. (China), with sequences listed in Additional File 1: Table S2. Brief procedures were mentioned in Additional File 2. Independent experiments were repeated at least three times.

### Glucose uptake and lactate production detection assays

Glucose uptake detection assay was conducted with the Glucose Oxidase Method Kit (APPLYGEN, China), and the lactate production detection assay was conducted with the Lactic Acid Assay kit (Nanjing Jiancheng Bioengineering Institute, China). Brief procedures were mentioned in Additional File 2. Independent experiments were repeated at least three times.

### Glycolysis stress assay

Extracellular acidification rate (ECAR) of NKTCL cells was conducted with Seahorse XF96 Flux Analyzer (Agilent, USA) and Glycolysis Stress Test Kit (Agilent, USA). Cell adhesion was described in Additional File 2. Independent experiments were repeated at least three times.

### Mass spectrometry analysis

Total sample preparation, mass spectrometry detection, and data analysis were performed as previously [19]. Brief procedures were mentioned in Additional File 2. The raw sequencing data was mentioned in Additional File 3.

### IP, CO-IP, and silver staining

Silver staining was conducted with Fast Silver Stain Kit (Beyotime, China). Brief procedures were mentioned in Additional File 2. The antibodies were listed in Additional File 1: Table S1.

### Western blotting

Brief procedures were mentioned in Additional File 2. Primary and secondary antibodies were listed in Additional File 1: Table S1.

### Xenograft tumor assay

BALB/c‐Nu nude mice and NOD-Scid mice were purchased from the GemPharmatech Company (China). Brief procedures were mentioned in Additional File 2. Animals were randomly selected and at least five animals in each group. The antibodies were listed in Additional File 1: Table S1.

### Statistical analysis

Statistical analyses were performed using SPSS software version 25.0 (IBM Corp., USA) and GraphPad Prism version 8.0 (GraphPad Software, Inc., USA). Data was expressed as the mean ± standard deviation for repeated measurements. Comparisons between groups were performed using Student’s *t*-test and analysis of variance. PFS and OS were analyzed using the Kaplan–Meier method and log-rank test. The correlation between LMP1 expression with clinical features and treatment response was assessed using the χ^2^-test. A value of *P* < 0.05 was considered statistically significant.

## Results

### LMP1 influences the aggressive biological behaviors of NKTCL cells and is related to the clinical characteristics of patients

**Fig. 1:**
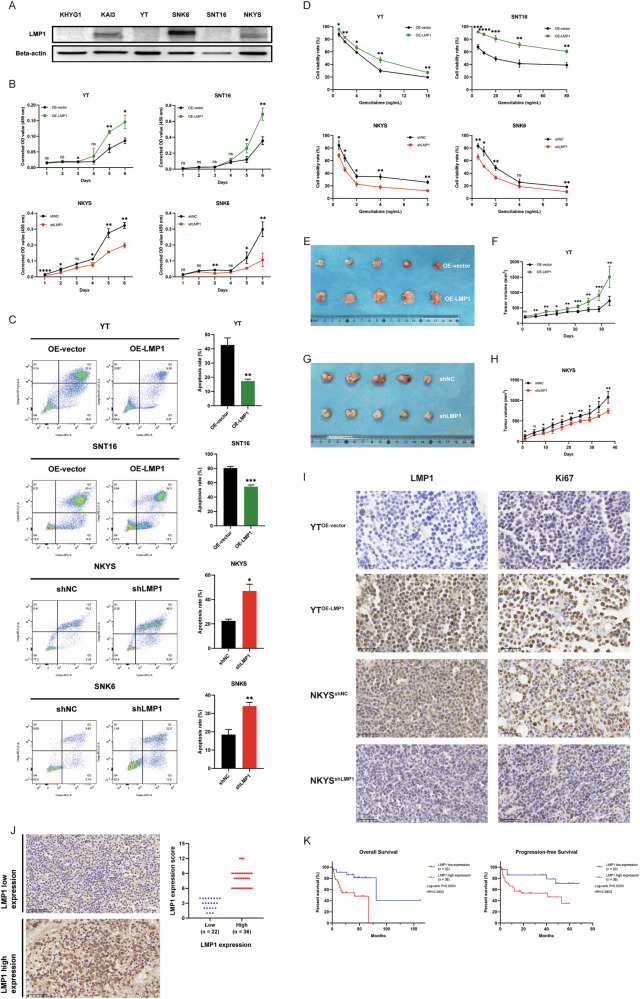
LMP1 influences the aggressive biological behaviors of NKTCL cells and is related to the clinical prognosis of patients. **A** LMP1 expression was examined in 6 NKTCL cell lines by western blotting. **B** Cell proliferation was detected by CCK-8. **C** Starvation-induced apoptosis was detected by flow cytometry. **D** Gemcitabine sensitivity was detected by CCK-8. **E** Representative images of the xenograft tumors of YT cells. **F** Tumor volumes of YT cells were analyzed. **G** Representative images of the xenograft tumors of NKYS cells. **H** Tumor volumes of NKYS cells were analyzed. **I** Representative IHC staining (400×) of LMP1 and Ki67 of the NKTCL xenograft tumor tissues. **J** Typical IHC staining (400×) and LMP1 expression scores of low and high LMP1 expression of the NKTCL patients. **K** Kaplan–Meier analysis of the correlation between the OS and PFS with the LMP1 expression. NKTCL natural killer/T cell lymphoma, CCK-8 Cell Counting Kit-8, IHC immunohistochemistry, OS overall survival, PFS progression-free survival, OD optical density, HR Hazard Ratio, ns no significance, *P < 0.05; **P < 0.01; ***P < 0.001; ****P < 0.0001.

To determine the role of LMP1 in the tumorigenesis and development of NKTCL, we examined LMP1 expression in 6 NKTCL cell lines (Fig. 1A). From this, we selected YT and SNT16 to generate OE-LMP1 cells (Additional File 4: Fig. S1A), and NKYS and SNK6 to generate shLMP1 cells (Additional File 4: Fig. S1B). Subsequently, cell proliferation, apoptosis resistance, and gemcitabine sensitivity were assessed in vitro. As expected, LMP1 overexpressing NKTCL cells, including YT^OE-LMP1^, SNT16^OE-LMP1^ cells, NKYS^shNC^, and SNK6^shNC^ cells, exhibited significantly enhanced cell proliferation (Fig. 1B), inhibition of the starvation-induced apoptosis (Fig. 1C) and reduced gemcitabine sensitivity (Fig. 1D). The role of LMP1 in tumorigenesis and tumor growth in vivo was also assessed in NKTCL xenograft mouse models (Additional File 4: Fig. S1C). We found that the mice injected with YT^OE-LMP1^ cells exhibited a greater tumor burden than the mice injected with YT^OE-vector^ cells (Fig. 1E, F) and that the mice injected with NKYS^shLMP1^ cells exhibited a lower tumor burden (Fig. 1G, H). In addition, the expression of Ki67 was related to the expression of the related protein LMP1 in mouse tissues (Fig. 1I). Furthermore, LMP1 expression was analyzed and scored by IHC staining in samples from 58 patients with pathologically verified NKTCL from the First Affiliated Hospital of Zhengzhou University (Fig. 1J). Interestingly, we found that LMP1 expression was significantly related to the Prognostic Index for Natural Killer cell lymphoma-Epstein-Barr virus (PINK-E) score of NKTCL patients (Table 1). Moreover, the results demonstrated that patients with low LMP1 expression were more likely to achieve a CR or a PR after treatment (Table 2), and patients with aberrantly high LMP1 expression had a worse prognosis, indicated by shorter OS and PFS (Fig. 1K). Taken together, these findings indicate that LMP1 supports NKTCL cell biological functions both in vitro and in vivo and is associated with the risk stratification, treatment response, and prognosis of NKTCL patients.

**Table 2:** Treatment response and the correlation with LMP1 expression of enrolled NKTCL patients.

Treatment response	*N*	LMP1 high expression	LMP1 low expression	*P*-value
Total	58	36	22	
**ORR**				**0.0307**
Yes (CR + PR)	43	23 (63.9%)	20 (90.9%)	
No (SD + PD)	15	13 (36.1%)	2 (9.1%)	

*NKTCL* natural killer/T cell lymphoma, *N* number, *ORR* overall response rate, *CR* complete response, *PR* partial response, *SD* stable disease, *PD* progressive disease.

The bold values include the main characteristics and the *P* value with statistical significance.

### LMP1 influences the aerobic glycolysis in NKTCL cells and is related to SUVmax of patients

**Fig. 2:**
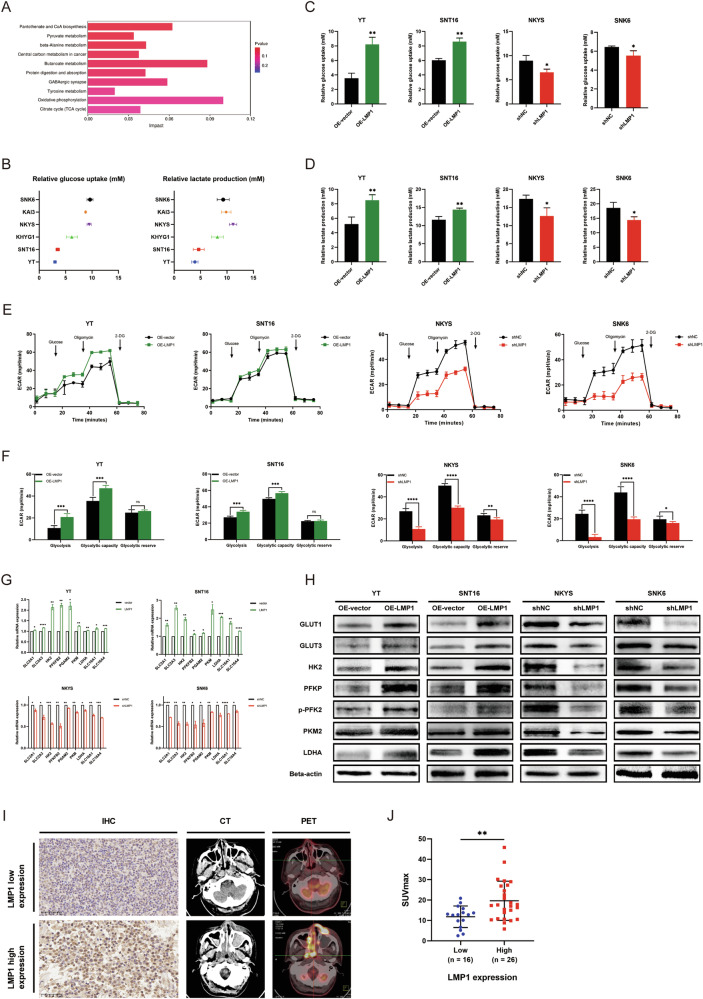
LMP1 influences the aerobic glycolysis in NKTCL cells and is related to SUVmax of patients. **A** KEGG enrichment of untargeted metabolomic sequencing between NKYS^shNC^ and NKYS^shLMP1^ cells. **B** Relative glucose uptake and lactate production of 6 NKTCL cell lines were detected and analyzed. **C** Relative glucose uptake was detected and analyzed. **D** Relative lactate production was detected and analyzed. **E** ECAR was detected by glycolysis stress assay. **F** Glycolysis, glycolytic capacity, and glycolytic reserve were analyzed. **G** Relative mRNA level of glycolysis-related genes was detected and analyzed by ddPCR. **H** Relative protein expression of glycolysis-related proteins was detected by western blotting. **I** Typical IHC staining (400×) of low and high LMP1 expression along with the corresponding CT and PET-CT images of the NKTCL patients. **J** The correlation of LMP1 expression of the NKTCL patients and their SUVmax was analyzed. NKTCL natural killer/T cell lymphoma, KEGG Kyoto Encyclopedia of Genes and Genomes, ECAR extracellular acidification rate, ddPCR droplet digital PCR, IHC immunohistochemistry, SUVmax maximum standardized uptake value, ns no significance, *P < 0.05; **P < 0.01; ***P < 0.001; ****P < 0.0001.

To explore the functional role of metabolism in LMP1-enhanced NKTCL cell proliferation, untargeted metabolomics sequencing was conducted, and 778 differentially expressed metabolites between NKYS^shNC^ and NKYS^shLMP1^ cells were identified (Additional File 4: Fig. S2A–D). KEGG enrichment analysis revealed that the main differentially expressed metabolic pathways were glucose-related metabolic pathways (Fig. 2A), and the function of LMP1 in aerobic glycolysis in NPC has been confirmed [20]. Therefore, we assessed glucose uptake and lactate production in the 6 NKTCL cell lines. Interestingly, the LMP1-positive cell lines SNK6, KAI3, and NKYS took up more glucose and produced more lactate than did the LMP1-negative cell lines KHYG1, SNT16 and YT (Fig. 2B). OE-LMP1 and shLMP1 had the same effects on YT and SNT16 cells (Fig. 2C), and on NKYS and SNK6 cells (Fig. 2D). Moreover, the changes in the ECAR induced by LMP1 were measured using a glycolysis stress assay. As expected, the OE-LMP1 cells had an increased ECAR, while the shLMP1 cells had a significantly decreased ECAR (Fig. 2E, F). In addition, the expression of glycolysis-related genes and proteins was also investigated and recapitulated (Fig. 2G, H). Subsequently, ^18^F-FDG uptake was analyzed in 42 NKTCL patients with LMP1 expression scored who underwent PET-CT examination before treatment. These findings suggested that aberrantly high LMP1 expression was accompanied by a greater SUVmax than low LMP1 expression (Fig. 2I, J). In summary, these results demonstrate that LMP1 strengthens NKTCL cell biological function by contributing to increased aerobic glycolysis.

### LMP1 regulates aerobic glycolysis via competitive binding with TRAF3 to activate the noncanonical NF-κB pathway

**Fig. 3:**
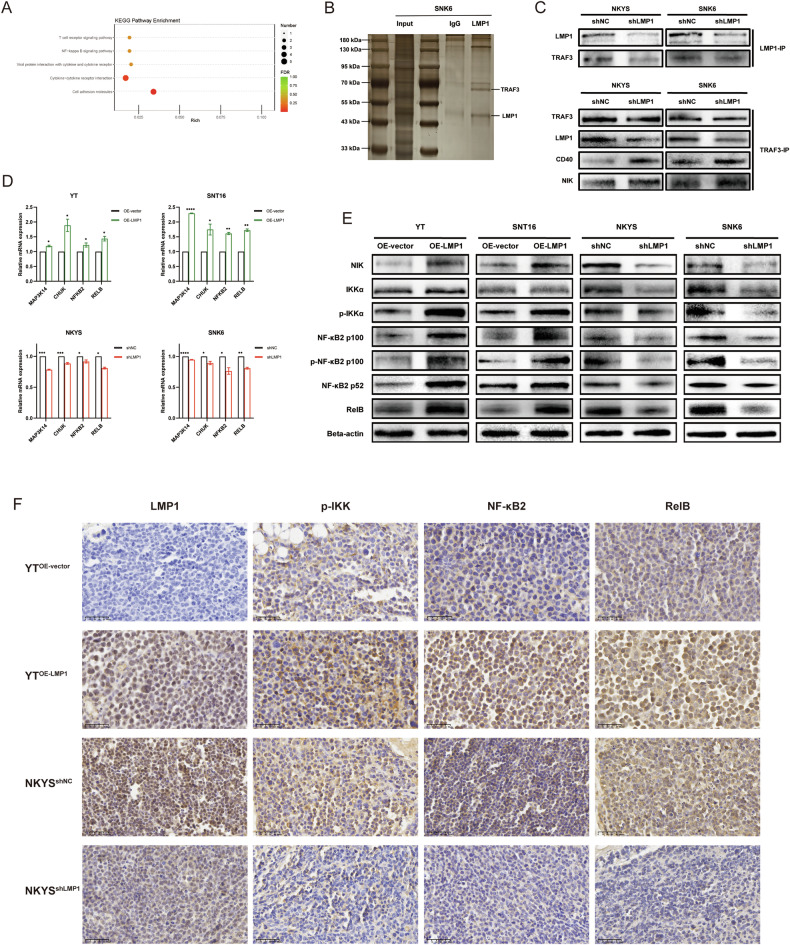
LMP1 regulates aerobic glycolysis via competitive binding with TRAF3 to activate the noncanonical NF-κB pathway. **A** KEGG enrichment of mRNA sequencing between NKYS^shNC^ and NKYS^shLMP1^ cells. **B** Silver staining image of SNK6 cell lysates immunoprecipitated with aniti-LMP1 antibody (covalently conjugated to agarose beads). **C** LMP1 (upper panels) and TRAF3 (bottom panels) were immunoprecipitated from lysates of the indicated shLMP1 cell lines of NKYS and SNK6, and then immunoprecipitates were subjected to SDS-PAGE and western blotting with the antibodies for the indicated proteins. **D** Relative mRNA level of noncanonical NF-κB-related genes was detected and analyzed by ddPCR. **E** Relative protein expression of noncanonical NF-κB-related proteins was detected by western blotting. **F** Representative IHC staining (400×) of LMP1 and noncanonical NF-κB-related proteins in the NKTCL xenograft tumor tissues. KEGG Kyoto Encyclopedia of Genes and Genomes, ddPCR droplet digital PCR, IHC immunohistochemistry, NKTCL natural killer/T cell lymphoma, *P < 0.05; **P < 0.01; ***P < 0.001; ****P < 0.0001.

**Fig. 4:**
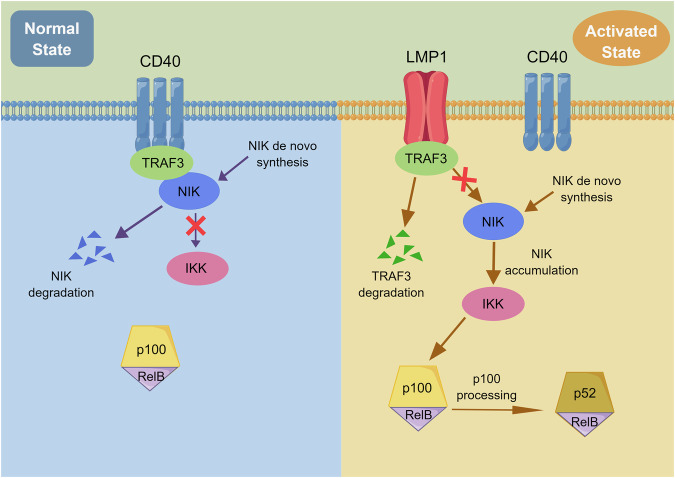
Graphic model of LMP1 competitive binding with TRAF3 to regulate the noncanonical NF-κB pathway in NKTCL cells. NKTCL natural killer/T cell lymphoma.

The underlying mechanism by which LMP1 regulates aerobic glycolysis in NKTCL remains unclear, and to address this problem, we performed RNA sequencing using RNA extracted from NKYS^shNC^ and NKYS^shLMP1^ cells, and KEGG enrichment analysis indicated that the NF-κB signaling pathway was significantly enriched in the shNC group (Fig. 3A). Furthermore, we identified one LMP1 binding partner, TRAF3, from the SNK6 cell line using IP coupled to mass spectrometry according to Significance Analysis of the INTeractome (SAINT) score [21, 22] (Fig. 3B, Additional File 3). It has been previously reported that LMP1 sequesters and binds TRAF3 more effectively than CD40 to regulate the NF-κB pathway in B-cell lymphoma cells [23]. Accordingly, a CO-IP assay was implemented to verify the competitive binding of LMP1 and CD40 with TRAF3 in NKTCL cells. The results showed that NKYS^shNC^ and SNK6^shNC^ cells bound more TRAF3, and NKYS^shLMP1^ and SNK6^shLMP1^ cells bound more CD40 (Fig. 3C). Depending on the dysregulated function of TRAF3 in the noncanonical NF-κB pathway, degradation of TRAF3 after competitive binding to LMP1 with increased affinity also promoted the accumulation of NIK, which activated the downstream noncanonical NF-κB signaling pathway, as confirmed by CO-IP assay (Fig. 3C). The expression of noncanonical NF-κB pathway-related genes and proteins was tested by ddPCR (Fig. 3D) and western blotting in NKTCL cells (Fig. 3E), and by IHC staining in NKTCL xenograft tumor tissues (Fig. 3F), and these results collectively suggested that shLMP1 inhibited the activation of the downstream noncanonical NF-κB pathway. In brief, LMP1 competes with CD40 for TRAF3 binding and subsequently activates the noncanonical NF-κB signaling pathway in NKTCL cells to promote aerobic glycolysis (Fig. 4).

### Activation of the noncanonical NF-κB pathway affects aerobic glycolysis in NKTCL cells

**Fig. 5:**
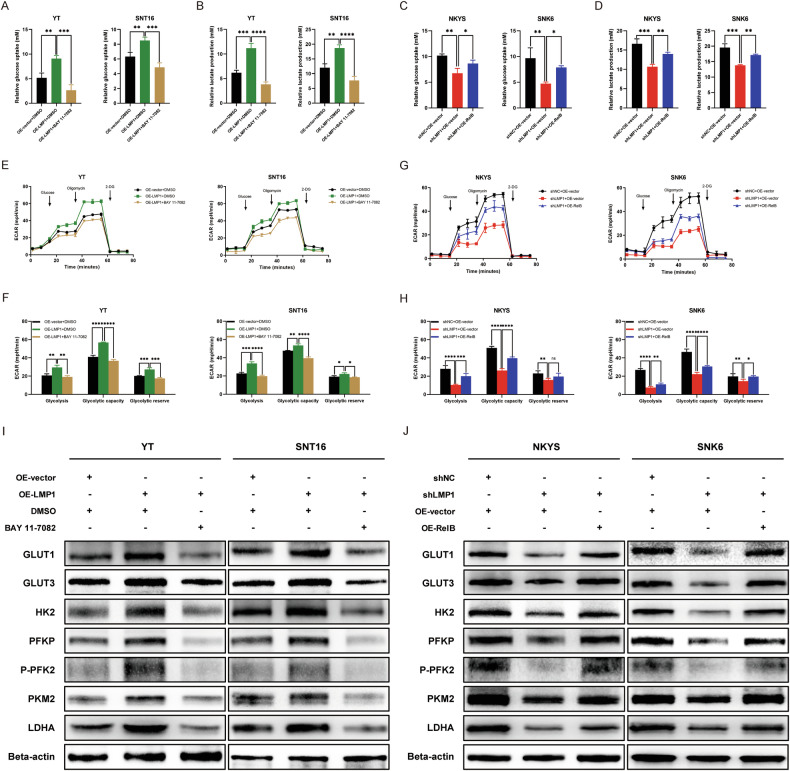
Activation of the noncanonical NF-κB pathway affects aerobic glycolysis in NKTCL cells. **A** Relative glucose uptake was detected and analyzed with inhibition of NF-κB pathway. **B** Relative lactate production was detected and analyzed with inhibition of NF-κB pathway. **C** Relative glucose uptake was detected and analyzed with reactivation of NF-κB pathway. **D** Relative lactate production was detected and analyzed with reactivation of NF-κB pathway. **E** ECAR was detected by glycolysis stress assay with inhibition of NF-κB pathway. **F** Glycolysis, glycolytic capacity, and glycolytic reserve were analyzed with inhibition of NF-κB pathway. **G** ECAR was detected by glycolysis stress assay with reactivation of NF-κB pathway. **H** Glycolysis, glycolytic capacity, and glycolytic reserve were analyzed with reactivation of NF-κB pathway. **I** Relative protein expression of glycolysis-related proteins was detected by western blotting with inhibition of NF-κB pathway. **J** Relative protein expression of glycolysis-related proteins was detected by western blotting with reactivation of NF-κB pathway. NKTCL natural killer/T cell lymphoma, OE-RelB over-expression RelB, ECAR extracellular acidification rate, no significance, *P < 0.05; **P < 0.01; ***P < 0.001; ****P < 0.0001.

To confirm the effect of the noncanonical NF-κB pathway reactivation on aerobic glycolysis in NKTCL, NKYS^shLMP1^ and SNK6^shLMP1^ cells with OE-RelB were generated (Additional File 4: Fig. S3). In addition, the NF-κB pathway inhibitor BAY 11-7082 was applied to the YT and SNT16 cells with OE-LMP1. BAY 11-7082 greatly reduced glucose uptake and lactate production compared with those in OE-LMP1 cells (Fig. 5A, B), and NKYS^shLMP1+OE-RelB^ and SNK6^shLMP1+OE-RelB^ cells took up more glucose and produced more lactate than NKYS^shLMP1+OE-vector^ and SNK6^shLMP1+OE-vector^ cells (Fig. 5C, D). Moreover, the glycolysis stress assay indicated that the ECAR was significantly decreased by the application of BAY 11-7082 in YT and SNT16 cells (Fig. 5E, F) and increased by OE-RelB in NKYS and SNK6 cells (Fig. 5G, H). Furthermore, the expression of glycolysis-related proteins was also confirmed by western blotting (Fig. 5I, J). In summary, the inhibition or reactivation of the NF-κB pathway affects aerobic glycolysis in NKTCL cells.

### Activation of the noncanonical NF-κB pathway and aerobic glycolysis affect the aggressive biological function of NKTCL cells

**Fig. 6:**
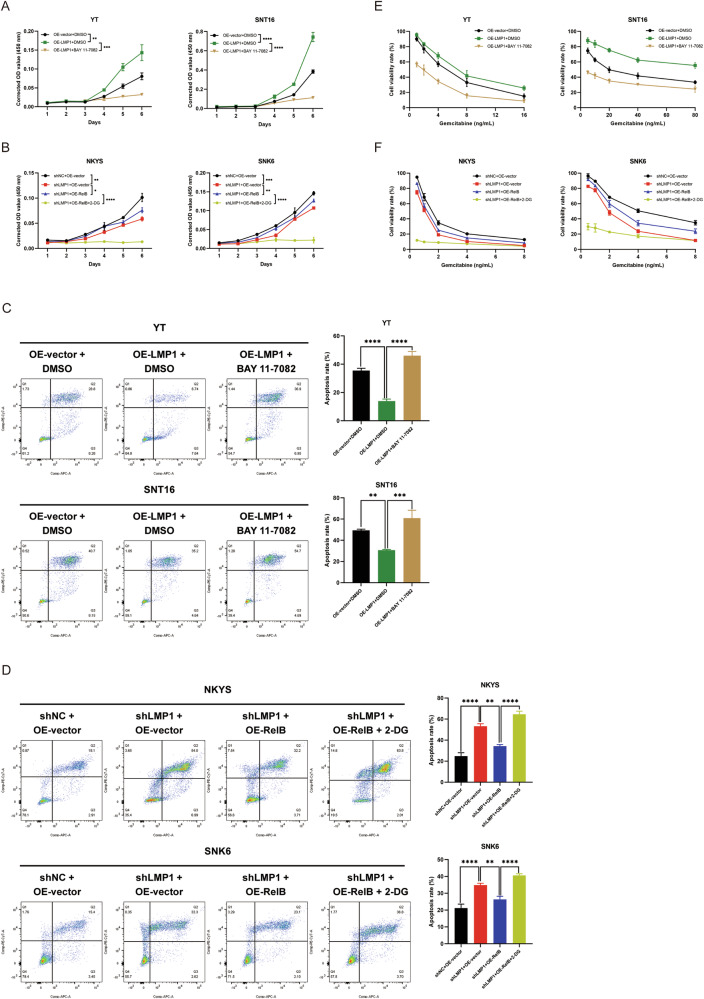
Activation of the noncanonical NF-κB pathway and aerobic glycolysis affect the aggressive biological function of NKTCL cells. **A** Cell proliferation was detected by CCK-8 with inhibition of NF-κB pathway. **B** Cell proliferation was detected by CCK-8 with reactivation of NF-κB pathway and inhibition of glycolysis. **C** Starvation-induced apoptosis was detected by flow cytometry with inhibition of NF-κB pathway. **D** Starvation-induced apoptosis was detected by flow cytometry with reactivation of NF-κB pathway and inhibition of glycolysis. **E** Gemcitabine sensitivity was detected by CCK-8 with inhibition of NF-κB pathway. **F** Gemcitabine sensitivity was detected by CCK-8 with reactivation of NF-κB pathway and inhibition of glycolysis. NKTCL natural killer/T cell lymphoma, CCK-8 Cell Counting Kit-8, no significance, *P < 0.05; **P < 0.01; ***P < 0.001; ****P < 0.0001.

Whether the activation of the noncanonical NF-κB pathway and aerobic glycolysis affect the tumorigenesis and development of NKTCL cells remains to be studied. Thus, cell proliferation, apoptosis resistance, and gemcitabine sensitivity were assessed in vitro. As expected, inhibition of the NF-κB pathway significantly suppressed the proliferation of NKTCL cells (Fig. 6A), and interestingly, OE-RelB attenuated the decrease in NKTCL cell proliferation caused by shLMP1, which was also inhibited by 2-DG, an aerobic glycolysis inhibitor (Fig. 6B). Consistently, in the apoptosis resistance and gemcitabine sensitivity experiments, BAY 11-7082 increased starvation-induced cell apoptosis (Fig. 6C), and OE-RelB decreased this effect, and the effect of OE-RelB was inhibited by treatment with 2-DG (Fig. 6D). Moreover, BAY 11-7082 reduced gemcitabine sensitivity in NKTCL cells (Fig. 6E) and OE-RelB promoted gemcitabine sensitivity, which was inhibited by 2-DG treatment (Fig. 6F). In summary, the activation of the noncanonical NF-κB pathway and aerobic glycolysis affect the proliferation and development of NKTCL cells modulated by LMP1.

## Discussion

Previous studies have comprehensively illuminated the phenotypic effects and mechanism of LMP1 on NPC. LMP1 plays an important role in the tumorigenesis and development of NPC through activating multiple signaling pathways, including cell proliferation and survival, angiogenesis, and invasion pathways [11, 24, 25]. In addition, LMP1 can affect cell-cell interactions, antigen presentation, cytokine and chemokine production, and modulation of the tumor microenvironment [10, 26, 27]. LMP1 is also reported to enhance cell proliferation in EBV-driven malignancies and serves as a prognostic marker for NKTCL patients [28, 29]. Therefore, we detected LMP1 expression in 6 NKTCL cell lines and established stable OE-LMP1 and shLMP1 cells. Our results showed that high LMP1 expression promoted NKTCL cell proliferation in vitro and in vivo, and was positively correlated with Ki67 expression in xenograft mouse tissues. In addition, experimental assays demonstrated that LMP1 inhibited starvation-induced apoptosis and reduced the gemcitabine sensitivity of NKTCL cells. Clinically, LMP1 expression was scored in 58 NKTCL patient tissues, and its correlations with clinical features, treatment response, and prognosis were analyzed. These findings suggest that LMP1 affects the tumorigenesis and development of NKTCL cells and serves as an indicator of the risk stratification, treatment response, and prognosis of NKTCL patients.

In recent years, studies on metabolic reprogramming, especially glucose metabolism, have attracted increased attention. Based on the results of untargeted metabolic sequencing, we found that compared with LMP1-negative cells, LMP1-positive NKTCL cells exhibited greater glucose uptake and lactate production. Consistent results were obtained for stable OE-LMP1 and shLMP1 cells. Moreover, the glycolysis stress assay suggested that the ECAR was affected by LMP1 expression. The expression of glycolysis-related genes and proteins also recapitulated this finding. In addition, we analyzed the available 18F-FDG uptake of NKTCL patients who underwent PET-CT examination before treatment, and the correlation between the SUVmax and LMP1 expression showed that aberrantly high LMP1 expression was accompanied by a greater SUVmax in NKTCL patients. Mechanistically, some studies have indicated that LMP1 promotes aerobic glycolysis by regulating oncogenic signaling pathways [17, 30], involving in epigenetic processes [31, 32], and activating glucose metabolic enzymes in NPC [20]. To date, few investigations have focused on the mechanism by which LMP1 regulates aerobic glycolysis in NKTCL, which is the novel aspect of our study.

To further explain the role of LMP1 in aerobic glycolysis in NKTCL, RNA sequencing was conducted and KEGG enrichment analysis revealed that the NF-κB signaling pathway was significantly enriched in the high LMP1 expression group. Subsequently, via IP coupled to mass spectrometry, we identified one LMP1-interacting protein, TRAF3, which plays a negative role in regulating the NF-κB signaling pathway [33]. Previous studies demonstrated that LMP1 led to TRAF3 sequestration in B-lymphoma cells, which inhibited the negative regulation of pro-survival membrane, cytoplasmic, and nuclear signaling events by TRAF3 [23]. Therefore, the interactions between LMP1 and TRAF3 and between TRAF3 and CD40 upon LMP1 availability were examined in our research. The results showed that cells with high LMP1 expression exhibited greater binding of LMP1 and TRAF3, and shLMP1 cells exhibited greater binding of LMP1 and CD40. Because of its competitive binding to LMP1 with greater affinity, the subsequent degradation of TRAF3 also protected NIK from self-degradation and subsequently caused its accumulation, which activated the downstream noncanonical NF-κB signaling pathway. The expression of noncanonical NF-κB pathway-related genes and proteins also confirmed this phenomenon.

Several studies have shown that the NF-κB pathway governs glycolysis via direct engagement of the cellular networks, with profound implications on inflammation, metabolic diseases, and tumorigenesis [34, 35]. A study on diffuse large B-cell lymphoma (DLBCL) showed that TP53 mutations cooperated with c-Rel to promote NF-κB functions and led to enhanced invasion and metastasis in malignant cells [36]. Moreover, the activation of the NF-κB signaling pathway increased glucose uptake by inducing the plasma membrane localization of GLUT1, blocking apoptosis, and promoting B-cell lymphoma growth [37]. However, this effect on NKTCL is not fully understood, and whether the activation of the noncanonical NF-κB signaling pathway affects aerobic glycolysis and the biological function of NKTCL cells remains to be explored. Thus, in our study, an NF-κB pathway inhibitor was applied to YT^OE-LMP1^ and SNT16 ^OE-LMP1^ cells, and OE-RelB cells were established from NKYS^shLMP1^ and SNK6^shLMP1^ cells. The results showed that with the inhibition of the NF-κB pathway, NKTCL cells exhibited suppression of aerobic glycolysis including decreased glucose uptake, lactate production, and ECAR, while OE-RelB cells showed promotion of aerobic glycolysis. In addition, glycolysis-related genes and proteins were also examined. Further experiments confirmed that inhibition of the NF-κB pathway could suppress the aggressive behavior of NKTCL cells and that of OE-RelB cells could restore these behaviors. Moreover, the addition of 2-DG, an inhibitor of glycolysis, significantly inhibited biological cellular functions.

Recently, accumulating evidence has also revealed the immune-related effects of LMP1 in NKTCL. Li et al. highlighted the crucial role of malignant NK cells with LMP1 expression in reshaping the cellular landscape and fostering an immunosuppressive microenvironment [38], and two other studies suggested that LMP1 expression is positively correlated with PD-L1 expression in NKTCL [8, 39]. In addition, LMP1 also serves as a promising target for novel adaptive T cell immunotherapy for the treatment of NKTCL [40]. Moreover, glycolysis has been implicated in the regulation of the tumor microenvironment and development by inhibiting monocyte migration, suppressing T cell activation, and promoting the release of cytokines in DLBCL [41]. Thus, whether enhancement of aerobic glycolysis is involved in the regulation of the NKTCL microenvironment and whether immunotherapy can attenuate abnormal glycolysis in NKTCL cells need to be elucidated in further studies.

In summary, the present study is the first to reveal the role and detailed mechanism of LMP1 in promoting aerobic glycolysis and aggressive biological functions in NKTCL. Our results elucidate the effects of viral infection on abnormal metabolism in NKTCL patients, which expands the understanding of the pathogenesis and progression of this disease and might provide a promising perspective for the treatment of NKTCL.

### Supplementary information

Additional File 1

Additional File 2

Additional File 3

Additional File 4

Checklist

Original Data File

## Data Availability

Raw untargeted metabolic sequencing data are available in the Metabolights under the accession number MTBLS9482. Raw mRNA sequencing data are deposited in the NCBI BioProject under the accession number PRJNA1071043. Any other data is available from the authors upon reasonable request.
