# Supplementary material for: LMP1 enhances aerobic glycolysis in natural killer/T cell lymphoma
Source: Cell Death Dis. 2024 Aug 20;15(8):604. doi: 10.1038/s41419-024-06999-7 (PMC11335758; doi:10.1038/s41419-024-06999-7)
Supplement: Supplementary file 1 — Additional File 1 [file 41419_2024_6999_MOESM1_ESM.pdf]

Table S1. Information of antibodies.

Name (anti-)	Cat No.	Concentration	Application	Corporation
LMP1	ab78113	1:200	IHC	Abcam
		1:1000	WB	
		1:50	IP	
Ki67	28074-1-AP	1:2000	IHC	Proteintech
GLUT1	66290-1-lg	1:1000	WB	Proteintech
GLUT3	20403-1-AP	1:500	WB	Proteintech
HK2	66974-1-lg	1:5000	WB	Proteintech
PFKP	68129-1-lg	1:5000	WB	Proteintech
P-PFK2	sc-373806	1:50	WB	Santa Cruz
PKM2	#4053	1:1000	WB	Cell Signaling Technology
LDHA	19978-1-AP	1:2000	WB	Proteintech
TRAF3	#4729	1:1000	WB	Cell Signaling Technology
		1:50	IP	
TRAF3	sc-6933	1:200	WB	Santa Cruz
		2μg/500μg	IP	
CD40	#86165	1:1000	WB	Cell Signaling Technology
CD40	sc-13128	1:1000	WB	Santa Cruz
NIK	#4994	1:1000	WB	Cell Signaling Technology
IKKα	#11930	1:1000	WB	Cell Signaling Technology
p-IKKα/β	#2697	1:1000	WB	Cell Signaling Technology
		1:200	IHC	Cell Signaling Technology
NF-κB2 p100/p52	#4882	1:1000	WB	Cell Signaling Technology
	15503-1-AP	1:200	IHC	Proteintech
p-NF-κB2 p100	#4810	1:1000	WB	Cell Signaling Technology
RelB	#4922	1:1000	WB	Cell Signaling Technology
	25027-1-AP	1:200	IHC	Proteintech
Beta-Actin	66009-1-lg	1:20000	WB	Proteintech
CD3	17617-1-AP	1:500	IHC	Proteintech
NCAM1/CD56	14255-1-AP	1:2000	IHC	Proteintech
Granzyme B	13588-1-AP	1:200	IHC	Proteintech
TIA-1	12133-2-AP	1:200	IHC	Proteintech
HRP-conjugated Affinipure Goat Anti-Rabbit IgG (H+L)	SA00001-2	1:5000	WB	Proteintech
HRP-conjugated Affinipure Goat Anti-Mouse IgG (H+L)	SA00001-1	1:5000	WB	Proteintech

IHC: immunohistochemistry; WB: western blotting; IP: immunoprecipitation.

Table S2. Primers for ddPCR.

Gene names	Sequencing (5’ to 3’)
Beta-actin-F	GAGAAAATCTGGCACCACACC
Beta-actin-R	GGATAGCACAGCCTGGATAGCAA
SLC2A1-F	TCACTGTGCTCCTGGTTCTG
SLC2A1-R	GCTCCTCGGGTGTCTTGTC
SLC2A3-F	GTGCAGCCCTTCCATTTT
SLC2A3-R	GGTGACTTGCTTTTCTTGTG
HK2-F	CACCTACGTGTGTGCTACCC
HK2-R	CTCCACCCCACTTCCCAT
PFKFB2-F	TTCCCCAAGAACCAAACCCC
PFKFB2-R	CCGGCTCCCAACACTGTAAT
PGAM2-F	CATTACGGGGGCCTCACA
PGAM2-R	TGCTTCTCGTCCATCGGG
PKM-F	ACAGCCAAAGGGGACTATC
PKM-R	GGCGGAGTTCCTCAAATA
LDHA-F	CTGTATGGAGTGGAATGAATG
LDHA-R	CAATAGCCCAGGATGTGTAG
SLC16A1-F	TTGTGGAATGCTGTCCTGTC
SLC16A1-R	CTTTTTCTGCTCGTTTGCTT
SLC16A4-F	GCAGGTATCCTTGAGACGGT
SLC16A4-R	AGTAGTGGAAATGTGGTGGC
MAP3K14-F	GCAGCTGGAAATAGAATTATTCCTC
MAP3K14-R	AATAGCTTGGGGTGTCGGTG
CHUK-F	TGTACACAGAGTTCTGCCCG
CHUK-R	TGCCCTGTTCCTCATTTGCC
NFKB2-F	AAGATTGAGCGGCCTGTAAC
NFKB2-R	CTCTTCCTTGTCTTCCACCAG
RELB-F	AAGTAGACATGAATGTGGTGAGGAT
RELB-R	TGATATGTCCTCTTTCTGCACCTT

ddPCR, droplet digital PCR.
