# Supplementary material for: LMP1 enhances aerobic glycolysis in natural killer/T cell lymphoma
Source: Cell Death Dis. 2024 Aug 20;15(8):604. doi: 10.1038/s41419-024-06999-7 (PMC11335758; doi:10.1038/s41419-024-06999-7)
Supplement: Supplementary file 2 — Additional File 2 [file 41419_2024_6999_MOESM2_ESM.pdf]

Evaluation criteria of immunohistochemical staining of LMP1

The score was accessed based on the staining intensity and the positively stained area. Staining intensity was divided into 0, 1, 2, and 3, which indicated no, weak, moderate and strong staining, respectively. The grades for positively stained cells included 1, 2, 3, and 4, which indicated a positively stained area of <25%, 25%–50%, 50%–75% and >75%, respectively. The score was calculated by multiplying staining intensity index and positively stained area grade, which could be 0, 1, 2, 3, 4, 6, 8, 9, and 12 score. 0-4 score was defined as low expression and 6-12 score was defined as high expression.

Cell lines and culture

YT, SNT16 and NKYS cell lines were cultured in RPMI 1640 medium (Invitrogen, USA) added to 10% heat-inactivated fetal bovine serum (FBS) (Clark Bioscience, USA) supplemented with 100U/mL penicillin and 100mg/mL streptomycin (Invitrogen, USA). SNK6 cell line was cultured in X-VIVO^{TM}15 serum-free hematopoietic cell medium (LONZA, Swiss) added to 10% heat-inactivated FBS (Clark Bioscience, USA). All cells were cultured in 5% CO_2 atmosphere at 37℃. Among them, NKYS and SNK6 were interleukin-2 (100IU/mL for NKYS and 500IU/mL for SNK6, SL Pharm, China)-dependent.

Construction of stable cell lines

Stable LMP1 and RelB OE cell lines were generated using lentiviral constructs of LMP1 or RelB (OE-LMP1/OE-RelB) and corresponding negative controls (OE-vector). Stable shLMP1 cell lines were generated using lentiviral constructs expressing short hairpin RNA (shRNA) of LMP1 (shLMP1) and negative control (shNC). Constructs encoding OE-LMP1, shLMP1 and OE-RelB and the packaging plasmids, that RRE, REV and VSVG for OE-LMP1, and PAX2 and MD2.G for shLMP1 and OE-RelB, were co-transfected into HEK-293T cells using Polyethylenimine (Polysciences, Ltd., USA). Supernatant containing recombinant lentivirus was collected 48h after transfection. A total of $1\text{-}5\times10^5$ cells were seeded in 24-well plates, which were infected with 40μL HitransG P solution and the lentivirus including OE-LMP1, shLMP1, OE-RelB, and corresponding controls. After more than 24h, the cells were transferred to the fresh medium, which included 0.25

to 0.5μg/mL Puromycin (Biosharp, Korea) or 10μg/mL Blasticidin S (Biosharp, Korea). Subsequently, the transfection efficiency was measured by flow cytometry and western blotting, and the stable cell lines were established for the following experiments.

Cell proliferation assay

Cells were seeded in 96-well plates at a density of 1 to 2×10^3 cells/well in 200μL of cell culture medium or mixture, containing 180μL of cell culture medium, 20μL of 5mM 2-Deoxy-D-glucose (2-DG) (Selleck, USA), and 20μL of 1-10μM BAY 11-7082 (Selleck, USA). 10μL Cell Counting Kit-8 (CCK-8) (UElandy, China) reagent was added to each well at the indicated days, and the cells were incubated at 37°C in the dark for 1h. Optical density (OD) value was measured with the Multiskan FC microplate reader (Thermo Scientific, USA). The corrected value was directly proportional to the number of viable cells.

Cell apoptosis assay

Cell apoptosis was analyzed using APC-Annexin V/PI Apoptosis Detection Kit (UElandy, China). After starvation with phosphate buffer saline (PBS) for 4 to 12h with or without treatment of 10mM 2-DG and 1-10μM BAY 11-7082, the cells in each group were fully harvested and resuspended in Annexin-V Binding Buffer. Then, the cells were stained with Annexin V-APC and PI Viability Staining Solution and incubated in dark for 15min at room temperature (RT) before being detected by FACSDiva software version 6.1.2 (BD Biosciences, USA) and analyzed by FlowJo software version 10.0 (Tree Star, Inc., USA).

Gemcitabine sensitivity assay

Cells were seeded in 96-well plates at a density of 1×10^4 cells/well in 100μL of mixture, containing 80μL of cell culture medium with or without 10μL of 5mM 2-DG and 10μL of 1-10μM BAY 11-7082, and 10μL gemcitabine (Selleck, USA) with different concentrations for 48h. After incubated with 10μL of CCK-8 reagent at 37°C in the dark for 1h, cell viability rate was evaluated as [OD (Gemcitabine treatment)–OD (Blank)] / [OD (Control)–OD (Blank)] × 100%.

mRNA sequencing analysis

Total RNA was isolated using the Trizol Reagent (Invitrogen Life Technologies), after which the concentration, quality and integrity were determined using a NanoDrop spectrophotometer (Thermo Scientific, USA). Three micrograms of RNA were used as input material for the RNA sample preparations. Sequencing libraries were generated according to the following steps. Firstly, mRNA was purified from total RNA using poly-T oligo-attached magnetic beads. Fragmentation was carried out using divalent cations under elevated temperature in an Illumina proprietary fragmentation buffer. First strand cDNA was synthesized using random oligonucleotides and Super Script II. Second strand cDNA synthesis was subsequently performed using DNA Polymerase I and RNase H. Remaining overhangs were converted into blunt ends via exonuclease/polymerase activities and the enzymes were removed. After adenylation of the 3′ ends of the DNA fragments, Illumina PE adapter oligonucleotides were ligated to prepare for hybridization. To select cDNA fragments of the preferred 400-500 bp in length, the library fragments were purified using the AMPure XP system (Beckman Coulter,Beverly, CA, USA). DNA fragments with ligated adaptor molecules on both ends were selectively enriched using Illumina PCR Primer Cocktail in a 15 cycle PCR reaction. Products were purified (AMPure XP system) and quantified using the Agilent high sensitivity DNA assay on a Bioanalyzer 2100 system (Agilent, USA). The sequencing library was then sequenced on NovaSeq 6000 platform (Illumina, USA) by Suzhou PANOMIX Biomedical Tech Co., LTD. (China).

Metabolic sequencing analysis

Samples preprocessing

1. Chemicals and reagents

LC-MS grade acetonitrile (ACN) and methanol (MeOH) were purchased from Fisher Scientific (Loughborough, UK). Formic acid was obtained from TCI (Shanghai, China). Ultrapure water was generated using a Milli-Q system (Millipore, Bedford, USA). 2-Amino-3-(2-chlorophenyl)-propionic acid was obtained from Aladdin (Shanghai, China).

2. Equipment.

High-speed centrifuge was obtained from Hunan Xiangyi Experiment Equipment Co., Ltd. (Hunan,

China). Vortex mixer was obtained from Haimen Kylin-bell Lab Instruments Co., Ltd. (Haimen, China). Centrifugal vacuum evaporator was from Eppendorf China Ltd. (Shanghai, China). Tissue grinder was obtained from Zhejiang Meibi Experiment Equipment Co., Ltd. (Zhejiang, China). Microporous membrane filters (0.22 μm) was purchased from Tianjin Jinteng Experiment Equipment Co., Ltd. (Tianjin, China). Glass bead was obtained from Sigma-Aldrich (Shanghai, China).

3. Sample preparation

3.1. Transfer all cell samples into a 2 mL centrifuge tube, add 100 mg glass bead;

3.2. Accurately add 1000 μL acetonitrile (ACN): methanol: H2O mixed solution (2:2:1, V / V / V) (stored at 4 °C), vortex for 30 s;

3.3. Put the centrifuge tube containing the sample into the 2 mL adapter matched with the instrument, immerse it in liquid nitrogen for rapid freezing for 5 min, take out the centrifuge tube and thaw at room temperature, put the centrifuge tube into the 2 mL adapter again, install it into the tissue grinder and grind it at 55 Hz for 2 min;

3.4. Repeat step 3.3. twice;

3.5. Take out the centrifuge tube, centrifuge for 10 min at 12,000 rpm and 4 °C, take all the supernatant, transfer it to a new 2 mL centrifuge tube, concentrate and dry it;

3.6. Accurately add 300 μL acetonitrile: 2-Amino-3-(2-chloro-phenyl)-propionic acid (4 ppm) solution prepared with 0.1% formic acid (1:9, V / V) (stored at 4 °C) to re-dissolve the sample, filter the supernatant by 0.22 μm membrane and transfer into the detection bottle for LC-MS detection.

Detection

1. Reagents

LC-MS grade acetonitrile (ACN) was purchased from Fisher Scientific (Loughborough, UK). Formic acid was obtained from TCI (Shanghai, China). Ammonium formate was obtained from Sigma-Aldrich (Shanghai, China). Ultrapure water was generated using a Milli-Q system (Millipore, USA).

2. Liquid chromatography conditions

The LC analysis was performed on a Vanquish UHPLC System (Thermo Fisher Scientific, USA). Chromatography was carried out with an ACQUITY UPLC ® HSS T3 (150×2.1 mm, 1.8 μm)

(Waters, USA). The column maintained at 40 °C. The flow rate and injection volume were set at 0.25 mL/min and 2 μL, respectively. For LC-ESI (+)-MS analysis, the mobile phases consisted of (C) 0.1% formic acid in acetonitrile (v/v) and (D) 0.1% formic acid in water (v/v). Separation was conducted under the following gradient: 0~1 min, 2% C; 1~9 min, 2%~50% C; 9~12 min, 50%~98% C; 12~13.5 min, 98% C; 13.5~14 min, 98%~2% C; 14~20 min, 2% C. For LC-ESI (-)-MS analysis, the analytes were carried out with (A) acetonitrile and (B) ammonium formate (5mM). Separation was conducted under the following gradient: 0~1 min, 2%A; 1~9 min, 2%~50%A; 9~12 min, 50%~98%A; 12~13.5 min, 98%A; 13.5~14 min, 98%~2%A; 14~17 min, 2%A.

3. Mass spectrum conditions

Mass spectrometric detection of metabolites was performed on Q Exactive (Thermo Fisher Scientific, USA) with ESI ion source. Simultaneous MS1 and MS/MS (Full MS-ddMS2 mode, data-dependent MS/MS) acquisition was used. The parameters were as follows: sheath gas pressure, 30 arb; aux gas flow, 10 arb; spray voltage, 3.50 kV and -2.50 kV for ESI (+) and ESI (-), respectively; capillary temperature, 325 °C; MS1 range, m/z 81-1000; MS1 resolving power, 70000 FWHM; number of data dependant scans per cycle, 10; MS/MS resolving power, 17500 FWHM; normalized collision energy, 30%; dynamic exclusion time, automatic.

Droplet digital PCR (ddPCR)

After RNA was extracted, the concentration and purity of extracted RNA were measured by Nanodrop 1000 spectrophotometry (Thermo Scientific, USA). The total volume of the ddPCR mixture was 20μL, and ddPCR was performed using a QX200 Droplet Digital PCR System (Bio-Rad, USA). Droplet were then detected on the QX200 Droplet Reader (Bio-Rad, USA) and analyzed using Quantasoft software version 1.7.4 (Bio-Rad, USA) with a user-defined threshold. Droplet positivity was deteteermined by the fluorescence intensity; only droplets above a minimum amplitude threshold were counted as positive. Beta-actin was used as a reference gene for mRNA quantitation. Independent experiments were repeated at least three times.

Glucose uptake and lactate production detection assays

Cells were seeded in 6-well plates at a density of 5×10^5 cells/well in 4mL of cell culture medium

mixture with or without treatment of 1-10μM BAY 11-7082 (Selleck, USA) for 48h. Next, the supernatants were collected by centrifugation to remove the cells, and the levels of glucose (APPLYGEN, China) and lactate (Nanjing Jiancheng Bioengineering Institute, China) were measured using the Multiskan FC microplate reader (Thermo Scientific, USA) according to the manufacturers' instructions.

Cell adhesion for glycolysis stress assay

Cells, with or without treated with 1-10μM BAY 11-7082 (Selleck, USA), were seeded in the 0.1mg/mL poly-L-lysine hydrobromide (Solarbio, China)-coated incubation plate at a density of 3×10^4 cells/well at 37°C for 30min to allow adhesion.

Mass spectrometry analysis

The desalted sample was dissolved in solvent A and analyzed via reverse-phase high-pressure liquid chromatography electrospray ionization tandem mass spectrometry (RP-HPLC-ESI-MS/MS) using a TripleTOF 5600+ mass spectrometer (SCIEX, Canada) coupled to a NanoSpray III ion source and a nanoLC Eksigent 415 system (SCIEX, Canada). Nano-scale reverse-phase liquid chromatography (RPLC) was performed with a trap-and-elution configuration using a Nano cHiPLC Trap column (200 μm × 0.5 mm ChromXP C18-CL 3 μm 120 Å) and a nano-scale analytical column (75 μm × 15 cm ChromXP C18-CL 3 μm 120 Å). Solvents A and B were 2 % and 98 % acetonitrile in water supplemented with 0.1 % formic acid. The sample was loaded in the trap column at a flow rate of 3 μL/min for 7 min using 100 % solvent A. For each sample, a stepwise gradient of 60 min (0 - 0.5 min, 95 - 92 % A; 0.5 - 38 min, 92 - 75 % A; 38 - 44 min, 75 – 65 % A; 44 - 45 min, 65 – 20 % A; 45 - 51 min, 20 % A; 51 - 52 min, 20 - 95 % A; 52 - 60 min, 95 % A) or 90 min (0 - 0.5 min, 95 - 93 % A; 0.5 - 55 min, 93 - 75 % A; 55 - 70 min, 75 - 65 % A; 70 - 75 min, 65 - 50 % A; 75 - 76 min, 50 - 20 % A; 76 - 81 min, 20 % A; 81 - 82 min, 20 - 95 % A; 82 - 90 min, 95 % A) was separately performed using a flow rate of 300 μL/min.

The following parameters were used with the mass spectrometer: MS survey scan range, 350 - 1500 m/z; MS survey, 0.25 s; MS/MS scan range, 100 - 1500 m/z. The precursor ions were fragmented

in the collision cell using rolling collision energy, and the collision energy spread (CES) was set to 10. Up to 30 precursorions were selected for subsequent MS/MS experiments with an accumulation time of 0.07 s per MS/MS scan and a total cycle time of 2.4 s. The selection criteria for precursor ions included an intensity greater than 200 cps and a charge state ranging from + 2 to + 5. The precursor ions were fragmented in the collision cell using rolling collision energy, and the CES was set to 5. The following parameters were used: dynamic excluded time, 18 s; ignore peaks within 6 Da; exclude after occurrences, 1; mass tolerance, 50 ppm.

The original .wiff files were converted to .mgf files using PeakView software (SCIEX, Canada), and the resulting files were subjected to database searches using Mascot 2.3 (Matrix Science, UK). The following parameters were used for database search: trypsin, KR/P; max cleavage sites, 2; fixed modification, carboxamidomethylation (C); variable modification, oxidation (M), deamidated (NQ), acetyl (N-term); mass tolerance, 0.05 Da for the precursor ion and 0.1 Da for the fragment ion. The searches were conducted in a UniProt Swiss-Prot database containing whole Homo sapiens proteins and the same number of reversed protein sequences. SAINT express (v3.6.1) was the statistical tool utilized to calculate the probability of protein–protein interaction from background, nonspecific interactions with a SAINT probability threshold of ≥ 0.8.

IP, CO-IP and silver staining

After washed with phosphate buffer saline (PBS), cells were lysed in cold IP lysis buffer (Beyotime, China) with protease and phosphatase inhibitor cocktail (Beyotime, China) for 30min on ice. The cell lysates were clarified by centrifugation at 13,000×g for 30min at 4°C. Cell lysate (1000μg) was combined with the antibody and 50μL agarose beads (Beyotime, China) and incubated overnight at 4°C with rotation. The antigen–antibody-agarose beads mixture was washed 5 to 10 times with cold PBS, and eluted before SDS-PAGE. Silver staining was conducted with Fast Silver Stain Kit (Beyotime, China) according to manufacture instructions.

Western blotting

After washed with PBS, cells were lysed in cold RIPA lysis buffer (CWBIO, China) and protease

and phosphatase inhibitor cocktail (Beyotime, China) for 30min on ice. The cell lysates were clarified by centrifugation at 13,000×g for 30min at 4℃. Proteins were resolved by SDS-PAGE and transferred onto polyvinylidene fluoride membranes (Millipore, USA). After blocked into tris buffered saline tween buffer containing 5% non-fat milk or 2% BSA at RT for 2h, the membranes incubated with primary antibodies at 4℃ overnight and secondary antibodies for 1h at RT. Detections was carried out with ECL Kit (UElandy, China). The band images were digitally captured and quantified with a ChemiDocTM XRC + system (Bio-Rad, USA).

Xenograft tumor assay

Female BALB/c-Nu nude mice (3 to 4 weeks old, 15 to 20g) and female NOD-Scid mice (3 to 4 weeks old, 15 to 20g) were used in this study. The mice were housed 3 to 5 per cage and fed a standard sterile laboratory diet under the humidity and temperature-controlled condition. Xenograft models were created by subcutaneous injection of 1×10^7 cells in 400μL of medium/Matrigel (Corning Incorporated, USA) (1:1 mixture) into the right axilla region, YT for BALB/c-Nu nude mice and NKYS for NOD-Scid mice. Since the tumors were formed for 15 to 20 days, the mice were given different treatments by intraperitoneal injection: 2-DG (100mg/kg, twice a week), BAY 11-7082(2.5mg/kg, twice a week), and corresponding controls, with condition and the tumor size monitored every 2 days. Tumor volume was calculated as: Volume=$ab^2/2$ (a meant the long diameter and b meant the short diameter). The expression of LMP1, Ki67, and noncanonical NF-κB pathway-related proteins were detected by IHC staining.
