# Supplementary material for: LMP1 enhances aerobic glycolysis in natural killer/T cell lymphoma
Source: Cell Death Dis. 2024 Aug 20;15(8):604. doi: 10.1038/s41419-024-06999-7 (PMC11335758; doi:10.1038/s41419-024-06999-7)
Supplement: Supplementary file 3 — Additional File 3 [file 41419_2024_6999_MOESM3_ESM.pdf]

Bait	Prey	PreyGene	Spec	SpecSum	AvgSpec	NumReplicates	ctrlCounts	AvgP	MaxP	TopoAvgP	TopoMaxP	SaintScore	FoldChange	FDR
LMP1	Q86TT2		62937	62937	62937	1	0	1	1	1	1	1	629370	0
LMP1	A2N0T1	VH6DJ	59928	59928	59928	1	0	1	1	1	1	1	599280	0
LMP1	Q13469	NFATC2	12791	12791	12791	1	0	1	1	1	1	1	127910	0
LMP1	P36578	RPL4	35766	35766	35766	1	0	1	1	1	1	1	357660	0
LMP1	P02768	ALB	40645	40645	40645	1	0	1	1	1	1	1	406450	0
LMP1	S6AWF0		10676	10676	10676	1	0	1	1	1	1	1	106760	0
LMP1	P35527	KRT9	65098	65098	65098	1	2973	1	1	1	1	1	21.9	0
LMP1	P01861	IGHG4	62719	62719	62719	1	11507	1	1	1	1	1	5.45	0
LMP1	Q9UL78		57083	57083	57083	1	0	1	1	1	1	1	570830	0
LMP1	A0A1K0GXZ1	GLNC1	48996	48996	48996	1	0	1	1	1	1	1	489960	0
LMP1	A8K3K1		61826	61826	61826	1	0	1	1	1	1	1	618260	0
LMP1	P13645	KRT10	40087	40087	40087	1	2496	1	1	1	1	1	16.06	0
LMP1	P19338	NCL	56127	56127	56127	1	17809	1	1	1	1	1	3.15	0
LMP1	A0A024RBS2	RPLP0	54194	54194	54194	1	13997	1	1	1	1	1	3.87	0
LMP1	A0A0C4DH25	IGKV3D-20	40059	40059	40059	1	3163	1	1	1	1	1	12.66	0
LMP1	Q96PK6	RBM14	21220	21220	21220	1	0	1	1	1	1	1	212200	0
LMP1	V9HW68	HEL-214	13632	13632	13632	1	0	1	1	1	1	1	136320	0
LMP1	P42766	RPL35	36881	36881	36881	1	3194	1	1	1	1	1	11.55	0
LMP1	Q02978	SLC25A11	44809	44809	44809	1	2256	1	1	1	1	1	19.86	0
LMP1	Q02543	RPL18A	54079	54079	54079	1	14278	1	1	1	1	1	3.79	0
LMP1	P62277	RPS13	25270	25270	25270	1	1999	1	1	1	1	1	12.64	0
LMP1	K4TZQ2	HLA-A	14238	14238	14238	1	0	1	1	1	1	1	142380	0
LMP1	A8MUS3	RPL23A	62973	62973	62973	1	0	1	1	1	1	1	629730	0
LMP1	A8K4W0	RPS3A	43370	43370	43370	1	0	1	1	1	1	1	433700	0
LMP1	P25398	RPS12	29245	29245	29245	1	8418	1	1	1	1	1	3.47	0
LMP1	P62244	RPS15A	2468	2468	2468	1	237	1	1	1	1	1	10.41	0
LMP1	P62249	RPS16	6167	6167	6167	1	2280	1	1	1	1	1	2.7	0
LMP1	Q53GG0		41954	41954	41954	1	14171	1	1	1	1	1	2.96	0
LMP1	A2RUM7	RPL5	3686	3686	3686	1	742	1	1	1	1	1	4.97	0
LMP1	S6AWE6		54182	54182	54182	1	0	1	1	1	1	1	541820	0
LMP1	H2AM05	HLA-A	31649	31649	31649	1	0	1	1	1	1	1	316490	0
LMP1	A0A120HG46		30793	30793	30793	1	0	1	1	1	1	1	307930	0
LMP1	Q6IPI1	RPL29	30547	30547	30547	1	6979	1	1	1	1	1	4.38	0
LMP1	Q8TBC3	SHKBP1	36426	36426	36426	1	0	1	1	1	1	1	364260	0
LMP1	A0A109PW65		32122	32122	32122	1	0	1	1	1	1	1	321220	0
LMP1	V9HWB4	HEL-S-89n	65518	65518	65518	1	25046	1	1	1	1	1	2.62	0
LMP1	E9PGV9	ABCG1	49249	49249	49249	1	4613	1	1	1	1	1	10.68	0
LMP1	Q562L9	ACT	49631	49631	49631	1	4537	1	1	1	1	1	10.94	0
LMP1	K7EM38	ACTG1	61107	61107	61107	1	8638	1	1	1	1	1	7.07	0
LMP1	Q9UL94		36255	36255	36255	1	5542	1	1	1	1	1	6.54	0
LMP1	P68366	TUBA4A	48227	48227	48227	1	18094	1	1	1	1	1	2.67	0
LMP1	A0A075B7D0	IGHV1OR15-1	21156	21156	21156	1	0	1	1	1	1	1	211560	0
LMP1	B1N7B8		25356	25356	25356	1	0	1	1	1	1	1	253560	0
LMP1	A0A0S2Z491	NPM1	8055	8055	8055	1	2105	1	1	1	1	1	3.83	0
LMP1	A0A0G2JLV0	TAP2	22698	22698	22698	1	0	1	1	1	1	1	226980	0
LMP1	P01714	IGLV3-19	41062	41062	41062	1	0	1	1	1	1	1	410620	0
LMP1	A0A024RAZ7	HNRPA1	64851	64851	64851	1	745	1	1	1	1	1	87.05	0
LMP1	S6BGE9		2626	2626	2626	1	0	1	1	1	1	1	26260	0
LMP1	S6BGD4		47755	47755	47755	1	15963	1	1	1	1	1	2.99	0
LMP1	Q8N6K7	SAMD3	21332	21332	21332	1	0	1	1	1	1	1	213320	0
LMP1	A0A0C4DG17	RPSA	43802	43802	43802	1	1502	1	1	1	1	1	29.16	0
LMP1	P06312	IGKV4-1	56684	56684	56684	1	16876	1	1	1	1	1	3.36	0
LMP1	A0A024RB85	PA2G4	10459	10459	10459	1	0	1	1	1	1	1	104590	0
LMP1	A2MYD0	V1-17	25372	25372	25372	1	3579	1	1	1	1	1	7.09	0
LMP1	F8WE88	MYO5A	63902	63902	63902	1	0	1	1	1	1	1	639020	0
LMP1	V9HVZ7	HEL-176	13697	13697	13697	1	95	1	1	1	1	1	144.18	0
LMP1	A2MYD2	V1-19	23190	23190	23190	1	0	1	1	1	1	1	231900	0
LMP1	A0A024R611	CORO1A	8363	8363	8363	1	0	1	1	1	1	1	83630	0
LMP1	A0A087WSY6	IGKV3D-15	46352	46352	46352	1	0	1	1	1	1	1	463520	0
LMP1	A0A0G2JPR0	C4A	5811	5811	5811	1	0	1	1	1	1	1	58110	0
LMP1	P01859	IGHG2	33758	33758	33758	1	2744	1	1	1	1	1	12.3	0
LMP1	Q9BRR6	ADPGK	38608	38608	38608	1	0	1	1	1	1	1	386080	0
LMP1	A0A1B0GUU9	IGHM	21798	21798	21798	1	0	1	1	1	1	1	217980	0
LMP1	C9JIJ7	ANKRD7	36464	36464	36464	1	0	1	1	1	1	1	364640	0
LMP1	D3DTX6	PPP1R9B	51984	51984	51984	1	5813	1	1	1	1	1	8.94	0
LMP1	A0A087X0X3	HNRNPM	33750	33750	33750	1	10716	1	1	1	1	1	3.15	0
LMP1	O15144	ARPC2	23768	23768	23768	1	7470	1	1	1	1	1	3.18	0
LMP1	A0A024R5X2		8858	8858	8858	1	0	1	1	1	1	1	88580	0
LMP1	K7EK43	TUBB6	63845	63845	63845	1	0	1	1	1	1	1	638450	0
LMP1	Q6MZQ6	DKFZp686G11190	29794	29794	29794	1	2605	1	1	1	1	1	11.44	0
LMP1	F8WCF6	ARPC4-TTLL3	60602	60602	60602	1	6015	1	1	1	1	1	10.08	0
LMP1	A0A0B4J1Y9	IGHV3-72	42516	42516	42516	1	9836	1	1	1	1	1	4.32	0
LMP1	A0A125U0V4		63690	63690	63690	1	9508	1	1	1	1	1	6.7	0
LMP1	Q6GMX6	IGH@	33302	33302	33302	1	4186	1	1	1	1	1	7.96	0
LMP1	Q6PIL8	IGK@	15183	15183	15183	1	0	1	1	1	1	1	151830	0
LMP1	Q9BUR5	APOO	10068	10068	10068	1	0	1	1	1	1	1	100680	0
LMP1	Q14103	HNRNPD	13613	13613	13613	1	340	1	1	1	1	1	40.04	0
LMP1	S0F3B9	HLA-DRB1	35828	35828	35828	1	11226	1	1	1	1	1	3.19	0
LMP1	F8W0W8	PPP1CC	5529	5529	5529	1	0	1	1	1	1	1	55290	0
LMP1	A0A0E3DC45	HLA-A	34869	34869	34869	1	0	1	1	1	1	1	348690	0
LMP1	P02533	KRT14	48658	48658	48658	1	14080	1	1	1	1	1	3.46	0
LMP1	Q59FF0		65413	65413	65413	1	0	1	1	1	1	1	654130	0
LMP1	B5BU08	U2AF1	14759	14759	14759	1	5277	1	1	1	1	1	2.8	0
LMP1	H0Y300	HP	37422	37422	37422	1	0	1	1	1	1	1	374220	0
LMP1	Q9NPP6		46709	46709	46709	1	1322	1	1	1	1	1	35.33	0
LMP1	A2NB45		26090	26090	26090	1	3383	1	1	1	1	1	7.71	0
LMP1	B2R7E8		35041	35041	35041	1	1922	1	1	1	1	1	18.23	0
LMP1	E7EMK3	FLOT2	23262	23262	23262	1	0	1	1	1	1	1	232620	0
LMP1	A0A024R8U5	SFRS2	17374	17374	17374	1	0	1	1	1	1	1	173740	0
LMP1	P07900	HSP90AA1	7415	7415	7415	1	2907	1	1	1	1	1	2.55	0
LMP1	Q12906	ILF3	40245	40245	40245	1	0	1	1	1	1	1	402450	0
LMP1	A0A0M3LD09	HLA-DRB1	14936	14936	14936	1	0	1	1	1	1	1	149360	0
LMP1	O75369	FLNB	24145	24145	24145	1	0	1	1	1	1	1	241450	0
LMP1	Q9NZ01	TECR	1316	1316	1316	1	0	1	1	1	1	1	13160	0
LMP1	A0A5E4		43473	43473	43473	1	0	1	1	1	1	1	434730	0
LMP1	G0WVA4	HLA-A	23211	23211	23211	1	0	1	1	1	1	1	232110	0
LMP1	B2RC41		40042	40042	40042	1	0	1	1	1	1	1	400420	0
LMP1	O00299	CLIC1	57140	57140	57140	1	10793	1	1	1	1	1	5.29	0
LMP1	P06396	GSN	25310	25310	25310	1	2604	1	1	1	1	1	9.72	0

LMP1	B4E368		42215	42215	42215	1	0	1	1	1	1	1	422150	0
LMP1	A0A024RAD5	DDOST	13528	13528	13528	1	624	1	1	1	1	1	21.68	0
LMP1	P50402	EMD	44619	44619	44619	1	15190	1	1	1	1	1	2.94	0
LMP1	A0A0A7C6R5	HLA-C	5072	5072	5072	1	0	1	1	1	1	1	50720	0
LMP1	Q6PIK1	IGL@	51776	51776	51776	1	0	1	1	1	1	1	517760	0
LMP1	J3KTA4	DDX5	2475	2475	2475	1	0	1	1	1	1	1	24750	0
LMP1	P55884	EIF3B	6000	6000	6000	1	0	1	1	1	1	1	60000	0
LMP1	O14980	XPO1	48764	48764	48764	1	6976	1	1	1	1	1	6.99	0
LMP1	A0A024RDG1	VDP	7661	7661	7661	1	0	1	1	1	1	1	76610	0
LMP1	P18085	ARF4	47562	47562	47562	1	0	1	1	1	1	1	475620	0
LMP1	Q53H12	AGK	51237	51237	51237	1	7398	1	1	1	1	1	6.93	0
LMP1	Q5NV84	V1-3	45398	45398	45398	1	11358	1	1	1	1	1	4	0
LMP1	Q53HW7		21429	21429	21429	1	0	1	1	1	1	1	214290	0
LMP1	Q9NR30	DDX21	55993	55993	55993	1	17514	1	1	1	1	1	3.2	0
LMP1	P61981	YWHAG	45103	45103	45103	1	0	1	1	1	1	1	451030	0
LMP1	A0A1C9J6T1		46627	46627	46627	1	13305	1	1	1	1	1	3.5	0
LMP1	A2IPI6		21638	21638	21638	1	1559	1	1	1	1	1	13.88	0
LMP1	Q9Y3I0	RTCB	56221	56221	56221	1	7625	1	1	1	1	1	7.37	0
LMP1	O60506	SYNCRIP	60901	60901	60901	1	0	1	1	1	1	1	609010	0
LMP1	A0A0C4DH36	IGHV3-38	45511	45511	45511	1	14119	1	1	1	1	1	3.22	0
LMP1	Q6GMX0		31198	31198	31198	1	10769	1	1	1	1	1	2.9	0
LMP1	B2R7B5		30447	30447	30447	1	1107	1	1	1	1	1	27.5	0
LMP1	Q53F64		16793	16793	16793	1	4239	1	1	1	1	1	3.96	0
LMP1	A0A024R6N1	TRAF3	32230	32230	32230	1	0	1	1	1	1	1	322300	0
LMP1	J3KQN4	RPL36A	6176	6176	6176	1	0	1	1	1	1	1	61760	0
LMP1	B2RMV2	CYTSA	53638	53638	53638	1	18645	1	1	1	1	1	2.88	0
LMP1	A0A0X9UWM4		60567	60567	60567	1	19979	1	1	1	1	1	3.03	0
LMP1	A0N2N3		59190	59190	59190	1	0	1	1	1	1	1	591900	0
LMP1	P11234	RALB	63059	63059	63059	1	13175	1	1	1	1	1	4.79	0
LMP1	Q59EC0		40713	40713	40713	1	5459	1	1	1	1	1	7.46	0
LMP1	A0A0A0MQX8	MBNL1	6569	6569	6569	1	0	1	1	1	1	1	65690	0
LMP1	P05362	ICAM1	15462	15462	15462	1	2344	1	1	1	1	1	6.6	0
LMP1	P62318	SNRPD3	28070	28070	28070	1	2209	1	1	1	1	1	12.71	0
LMP1	P63104	YWHAZ	56049	56049	56049	1	0	1	1	1	1	1	560490	0
LMP1	A0A0A0MQS9	LAMA4	58130	58130	58130	1	0	1	1	1	1	1	581300	0
LMP1	P62891	RPL39	36342	36342	36342	1	0	1	1	1	1	1	363420	0
LMP1	P20290	BTF3	23155	23155	23155	1	0	1	1	1	1	1	231550	0
LMP1	Q0KKI6		54109	54109	54109	1	0	1	1	1	1	1	541090	0
LMP1	Q9HBB9		29920	29920	29920	1	0	1	1	1	1	1	299200	0
LMP1	P10114	RAP2A	64274	64274	64274	1	7777	1	1	1	1	1	8.26	0
LMP1	U5YKD2	HLA-A	40995	40995	40995	1	0	1	1	1	1	1	409950	0
LMP1	Q8NC51	SERBP1	61765	61765	61765	1	11476	1	1	1	1	1	5.38	0
LMP1	P15918	RAG1	36218	36218	36218	1	0	1	1	1	1	1	362180	0
LMP1	P50990	CCT8	9250	9250	9250	1	3393	1	1	1	1	1	2.73	0
LMP1	B2R6V6		27844	27844	27844	1	0	1	1	1	1	1	278440	0
LMP1	Q01844	EWSR1	62866	62866	62866	1	12967	1	1	1	1	1	4.85	0
LMP1	Q14213	EBI3	14237	14237	14237	1	0	1	1	1	1	1	142370	0
LMP1	B1PS43	MYH11	24955	24955	24955	1	0	1	1	1	1	1	249550	0
LMP1	Q5SRQ6	CSNK2B	47701	47701	47701	1	0	1	1	1	1	1	477010	0
LMP1	B4DS05		29260	29260	29260	1	8751	1	1	1	1	1	3.34	0
LMP1	A0A024RBE7	TMPO	59069	59069	59069	1	15459	1	1	1	1	1	3.82	0
LMP1	A0A068LKQ8		1393	1393	1393	1	0	1	1	1	1	1	13930	0
LMP1	Q9H2L7		62226	62226	62226	1	0	1	1	1	1	1	622260	0
LMP1	Q59F99		1395	1395	1395	1	0	1	1	1	1	1	13950	0
LMP1	P34897	SHMT2	5787	5787	5787	1	0	1	1	1	1	1	57870	0
LMP1	A8K761	NDUFB10	34171	34171	34171	1	1837	1	1	1	1	1	18.6	0
LMP1	P09543	CNP	46682	46682	46682	1	58	1	1	1	1	1	804.86	0
LMP1	E7EVA0	MAP4	64594	64594	64594	1	4659	1	1	1	1	1	13.86	0
LMP1	P22087	FBL	62301	62301	62301	1	335	1	1	1	1	1	185.97	0
LMP1	A0A0N9MXA6	HLA-A	18547	18547	18547	1	0	1	1	1	1	1	185470	0
LMP1	P09496	CLTA	6905	6905	6905	1	0	1	1	1	1	1	69050	0
LMP1	A0A0D9SF53	DDX3X	40301	40301	40301	1	7727	1	1	1	1	1	5.22	0
LMP1	A2J422		48027	48027	48027	1	8808	1	1	1	1	1	5.45	0
LMP1	Q93008	USP9X	34118	34118	34118	1	0	1	1	1	1	1	341180	0
LMP1	O14893	GEMIN2	15810	15810	15810	1	0	1	1	1	1	1	158100	0
LMP1	P30876	POLR2B	18465	18465	18465	1	1987	1	1	1	1	1	9.29	0
LMP1	Q6N091	DKFZp686C02220	50312	50312	50312	1	145	1	1	1	1	1	346.98	0
LMP1	A8K897		31774	31774	31774	1	0	1	1	1	1	1	317740	0
LMP1	A8K4W2		60888	60888	60888	1	0	1	1	1	1	1	608880	0
LMP1	G1FM92		30362	30362	30362	1	0	1	1	1	1	1	303620	0
LMP1	Q8WXH0	SYNE2	61815	61815	61815	1	0	1	1	1	1	1	618150	0
LMP1	A0A0S2Z492	MCM3	55065	55065	55065	1	11890	1	1	1	1	1	4.63	0
LMP1	A0A0B4J2B5	IGHV3OR16-9	64614	64614	64614	1	0	1	1	1	1	1	646140	0
LMP1	A8K588		50434	50434	50434	1	0	1	1	1	1	1	504340	0
LMP1	B3KW67		37066	37066	37066	1	0	1	1	1	1	1	370660	0
LMP1	A2N2F4	VK3	29674	29674	29674	1	10270	1	1	1	1	1	2.89	0
LMP1	A0A140TA86	C19orf70	23879	23879	23879	1	0	1	1	1	1	1	238790	0
LMP1	A0A024R5H0	BANF1	64634	64634	64634	1	2558	1	1	1	1	1	25.27	0
LMP1	Q8IYV2	DDX20	29539	29539	29539	1	3883	1	1	1	1	1	7.61	0
LMP1	P10644	PRKAR1A	63945	63945	63945	1	0	1	1	1	1	1	639450	0
LMP1	Q0ZCH9		29872	29872	29872	1	1799	1	1	1	1	1	16.6	0
LMP1	G3XAB9	GSG1	5397	5397	5397	1	0	1	1	1	1	1	53970	0
LMP1	P0CG39	POTEJ	50967	50967	50967	1	0	1	1	1	1	1	509670	0
LMP1	P20851	C4BPB	63525	63525	63525	1	0	1	1	1	1	1	635250	0
LMP1	P79483	HLA-DRB3	38088	38088	38088	1	0	1	1	1	1	1	380880	0
LMP1	P13073	COX4I1	28138	28138	28138	1	0	1	1	1	1	1	281380	0
LMP1	O75964	ATP5MG	22766	22766	22766	1	0	1	1	1	1	1	227660	0
LMP1	V9HWA6	HEL32	11161	11161	11161	1	0	1	1	1	1	1	111610	0
LMP1	A0A0S2Z4L3	PROS1	5626	5626	5626	1	0	1	1	1	1	1	56260	0
LMP1	P13995	MTHFD2	2156	2156	2156	1	0	1	1	1	1	1	21560	0
LMP1	Q6IBR2	FARSLA	17967	17967	17967	1	0	1	1	1	1	1	179670	0
LMP1	A0A024RAC5	RCC2	52864	52864	52864	1	0	1	1	1	1	1	528640	0
LMP1	Q9HCN4	GPN1	9843	9843	9843	1	0	1	1	1	1	1	98430	0
LMP1	A8K5T7		40635	40635	40635	1	0	1	1	1	1	1	406350	0
LMP1	B4DU42		25263	25263	25263	1	0	1	1	1	1	1	252630	0
LMP1	M1VPF6	CD74-ROS1_C6;R32	17344	17344	17344	1	0	1	1	1	1	1	173440	0
LMP1	A0A024R2A7	LMAN1	34590	34590	34590	1	0	1	1	1	1	1	345900	0
LMP1	P50479	PDLIM4	780	780	780	1	0	1	1	1	1	1	7800	0
LMP1	B2RBP7		55651	55651	55651	1	12766	1	1	1	1	1	4.36	0
LMP1	A0A1C3PI56	HLA-DPB1	18137	18137	18137	1	0	1	1	1	1	1	181370	0

LMP1	P37108	SRP14	6443	6443	6443	1	0	1	1	1	1	1	64430	0
LMP1	O75165	DNAJC13	50350	50350	50350	1	1764	1	1	1	1	1	28.54	0
LMP1	B4DNE1		4283	4283	4283	1	0	1	1	1	1	1	42830	0
LMP1	P09382	LGALS1	63970	63970	63970	1	0	1	1	1	1	1	639700	0
LMP1	A0A0I9N852	HLA-DQB1	60077	60077	60077	1	0	1	1	1	1	1	600770	0
LMP1	Q9Y678	COPG1	12752	12752	12752	1	0	1	1	1	1	1	127520	0
LMP1	A8K5B6		44852	44852	44852	1	0	1	1	1	1	1	448520	0
LMP1	O75694	NUP155	38684	38684	38684	1	0	1	1	1	1	1	386840	0
LMP1	Q9NZM1	MYOF	17179	17179	17179	1	0	1	1	1	1	1	171790	0
LMP1	Q53FN7		56188	56188	56188	1	21316	1	1	1	1	1	2.64	0
LMP1	Q5SRD1	TIMM23B	16795	16795	16795	1	0	1	1	1	1	1	167950	0
LMP1	Q9P0J0	NDUFA13	16186	16186	16186	1	0	1	1	1	1	1	161860	0
LMP1	A0A090N7Y2	ABCF2	54511	54511	54511	1	0	1	1	1	1	1	545110	0
LMP1	Q8IVT5	KSR1	55604	55604	55604	1	9481	1	1	1	1	1	5.86	0
LMP1	O75396	SEC22B	40790	40790	40790	1	0	1	1	1	1	1	407900	0
LMP1	P17275	JUNB	38078	38078	38078	1	7489	1	1	1	1	1	5.08	0
LMP1	L0R588	C11orf48	46532	46532	46532	1	0	1	1	1	1	1	465320	0
LMP1	A2NYV0		39473	39473	39473	1	0	1	1	1	1	1	394730	0
LMP1	A0A0X9T0I7		22315	22315	22315	1	543	1	1	1	1	1	41.1	0
LMP1	H0Y886	NDUFB5	54618	54618	54618	1	0	1	1	1	1	1	546180	0
LMP1	P48668	KRT6C	6510	6510	6510	1	0	1	1	1	1	1	65100	0
LMP1	Q8NCA5	FAM98A	24343	24343	24343	1	0	1	1	1	1	1	243430	0
LMP1	O15371	EIF3D	41968	41968	41968	1	0	1	1	1	1	1	419680	0
LMP1	E9PBD8	LSP1	30043	30043	30043	1	0	1	1	1	1	1	300430	0
LMP1	A0A024R7J0	PRKACA	20764	20764	20764	1	0	1	1	1	1	1	207640	0
LMP1	P62304	SNRPE	10289	10289	10289	1	0	1	1	1	1	1	102890	0
LMP1	A0A1B0GW05	DPY19L1	5499	5499	5499	1	0	1	1	1	1	1	54990	0
LMP1	A0A0A0MT11	CAMK2G	57285	57285	57285	1	0	1	1	1	1	1	572850	0
LMP1	Q86YT6	MIB1	40618	40618	40618	1	11849	1	1	1	1	1	3.43	0
LMP1	A0A024R3W2	TOMM20	61874	61874	61874	1	17514	1	1	1	1	1	3.53	0
LMP1	A0A075B6H7	IGKV3-7	54764	54764	54764	1	0	1	1	1	1	1	547640	0
LMP1	Q96T51	RUFY1	54013	54013	54013	1	18200	1	1	1	1	1	2.97	0
LMP1	A0A024RCG2	SLC22A18	803	803	803	1	0	1	1	1	1	1	8030	0
LMP1	H7C2Q8	EBNA1BP2	39628	39628	39628	1	0	1	1	1	1	1	396280	0
LMP1	P60604	UBE2G2	37150	37150	37150	1	5170	1	1	1	1	1	7.19	0
LMP1	A0A087WYN9	DHX29	59761	59761	59761	1	20955	1	1	1	1	1	2.85	0
LMP1	A0A0U1RQC9	TP53	16509	16509	16509	1	0	1	1	1	1	1	165090	0
LMP1	B4DLN1		2934	2934	2934	1	0	1	1	1	1	1	29340	0
LMP1	P35249	RFC4	28849	28849	28849	1	0	1	1	1	1	1	288490	0
LMP1	Q9NSD9	FARSB	63445	63445	63445	1	0	1	1	1	1	1	634450	0
LMP1	Q12899	TRIM26	62740	62740	62740	1	0	1	1	1	1	1	627400	0
LMP1	A0A109PW33		61624	61624	61624	1	7073	1	1	1	1	1	8.71	0
LMP1	Q30155	HLA-DRB2	46705	46705	46705	1	0	1	1	1	1	1	467050	0
LMP1	Q53EX5		59132	59132	59132	1	0	1	1	1	1	1	591320	0
LMP1	Q9Y3F4	STRAP	9879	9879	9879	1	0	1	1	1	1	1	98790	0
LMP1	P18031	PTPN1	42305	42305	42305	1	0	1	1	1	1	1	423050	0
LMP1	P29692	EEF1D	14955	14955	14955	1	0	1	1	1	1	1	149550	0
LMP1	Q08J23	NSUN2	30042	30042	30042	1	0	1	1	1	1	1	300420	0
LMP1	B7Z6D1		21632	21632	21632	1	0	1	1	1	1	1	216320	0
LMP1	A8K719		49474	49474	49474	1	0	1	1	1	1	1	494740	0
LMP1	P07195	LDHB	59302	59302	59302	1	7714	1	1	1	1	1	7.69	0
LMP1	P05023	ATP1A1	35348	35348	35348	1	0	1	1	1	1	1	353480	0
LMP1	A0A0X9T0H6		25385	25385	25385	1	9590	1	1	1	1	1	2.65	0
LMP1	Q9BVP2	GNL3	6333	6333	6333	1	0	1	1	1	1	1	63330	0
LMP1	B2R7W4		22433	22433	22433	1	0	1	1	1	1	1	224330	0
LMP1	Q8WUK0	PTPMT1	49450	49450	49450	1	4911	1	1	1	1	1	10.07	0
LMP1	A0A024RDA9	EXOC1	5526	5526	5526	1	0	1	1	1	1	1	55260	0
LMP1	A0A024RDQ9	SLC7A1	17479	17479	17479	1	0	1	1	1	1	1	174790	0
LMP1	A8K6A2		58158	58158	58158	1	0	1	1	1	1	1	581580	0
LMP1	A0A024R7M7	JAK3	37062	37062	37062	1	0	1	1	1	1	1	370620	0
LMP1	B7Z809		53473	53473	53473	1	5219	1	1	1	1	1	10.25	0
LMP1	A0A0K3AWD6	HLA-DQB1	15032	15032	15032	1	0	1	1	1	1	1	150320	0
LMP1	G5EA09	SDCBP	43207	43207	43207	1	0	1	1	1	1	1	432070	0
LMP1	J9JIE6	TMCO1	55620	55620	55620	1	0	1	1	1	1	1	556200	0
LMP1	B0AZQ4		51681	51681	51681	1	0	1	1	1	1	1	516810	0
LMP1	Q6FGH9	DNCL1	54862	54862	54862	1	0	1	1	1	1	1	548620	0
LMP1	Q15306	IRF4	64136	64136	64136	1	10572	1	1	1	1	1	6.07	0
LMP1	B2RAR2		40174	40174	40174	1	0	1	1	1	1	1	401740	0
LMP1	Q9H3U1	UNC45A	31946	31946	31946	1	10444	1	1	1	1	1	3.06	0
LMP1	Q8TDN6	BRIX1	15027	15027	15027	1	2186	1	1	1	1	1	6.87	0
LMP1	P51116	FXR2	35971	35971	35971	1	0	1	1	1	1	1	359710	0
LMP1	A0A0A0MS14	IGHV1-45	63554	63554	63554	1	6501	1	1	1	1	1	9.78	0
LMP1	Q8IXB1	DNAJC10	47999	47999	47999	1	18811	1	1	1	1	1	2.55	0
LMP1	A0A109PVK5		16731	16731	16731	1	0	1	1	1	1	1	167310	0
LMP1	A8K521		65126	65126	65126	1	0	1	1	1	1	1	651260	0
LMP1	O14602	EIF1AY	30412	30412	30412	1	0	1	1	1	1	1	304120	0
LMP1	D3U460	HLA-A	28796	28796	28796	1	0	1	1	1	1	1	287960	0
LMP1	Q9HD42	CHMP1A	8196	8196	8196	1	1642	1	1	1	1	1	4.99	0
LMP1	Q8NF50	DOCK8	38894	38894	38894	1	0	1	1	1	1	1	388940	0
LMP1	K7ERQ8		59215	59215	59215	1	0	1	1	1	1	1	592150	0
LMP1	M0R0P8	MYO9B	34124	34124	34124	1	3915	1	1	1	1	1	8.72	0
LMP1	P19320	VCAM1	62902	62902	62902	1	0	1	1	1	1	1	629020	0
LMP1	B4E1J8		60588	60588	60588	1	0	1	1	1	1	1	605880	0
LMP1	Q02880	TOP2B	11290	11290	11290	1	0	1	1	1	1	1	112900	0
LMP1	G1EP57	HLA-A	42878	42878	42878	1	0	1	1	1	1	1	428780	0
LMP1	Q6MZU6	DKFZp686C15213	42026	42026	42026	1	14312	1	1	1	1	1	2.94	0
LMP1	O43464	HTRA2	41589	41589	41589	1	0	1	1	1	1	1	415890	0
LMP1	Q96F88	POP1	37549	37549	37549	1	0	1	1	1	1	1	375490	0
LMP1	P09622	DLD	15130	15130	15130	1	5006	1	1	1	1	1	3.02	0
LMP1	B2RCM2		31204	31204	31204	1	0	1	1	1	1	1	312040	0
LMP1	A0A024R2W3	PRKAR2A	48591	48591	48591	1	0	1	1	1	1	1	485910	0
LMP1	Q5SSJ5	HP1BP3	41573	41573	41573	1	0	1	1	1	1	1	415730	0
LMP1	A2N0U2	VH6DJ	38404	38404	38404	1	9090	1	1	1	1	1	4.22	0
LMP1	P16401	HIST1H1B	60833	60833	60833	1	0	1	1	1	1	1	608330	0
LMP1	H0YEN0	RIC8A	64655	64655	64655	1	0	1	1	1	1	1	646550	0
LMP1	Q96A33	CCDC47	34577	34577	34577	1	624	1	1	1	1	1	55.41	0
LMP1	X5DR09	GTF2I	41094	41094	41094	1	0	1	1	1	1	1	410940	0
LMP1	Q00688	FKBP3	37298	37298	37298	1	0	1	1	1	1	1	372980	0
LMP1	Q86WV6	TMEM173	13543	13543	13543	1	0	1	1	1	1	1	135430	0
LMP1	P43686	PSMC4	8773	8773	8773	1	0	1	1	1	1	1	87730	0

LMP1	O75306	NDUFS2	27109	27109	27109	1	0	1	1	1	1	1	271090	0
LMP1	B4DL31		42566	42566	42566	1	0	1	1	1	1	1	425660	0
LMP1	Q9NXS2	QPCTL	48608	48608	48608	1	3329	1	1	1	1	1	14.6	0
LMP1	A0A068LKR4		37987	37987	37987	1	0	1	1	1	1	1	379870	0
LMP1	Q5JTV8	TOR1AIP1	21463	21463	21463	1	0	1	1	1	1	1	214630	0
LMP1	B4DM85		6577	6577	6577	1	0	1	1	1	1	1	65770	0
LMP1	B2RAH5		40782	40782	40782	1	0	1	1	1	1	1	407820	0
LMP1	F4NBQ7	HLA-B	46319	46319	46319	1	7435	1	1	1	1	1	6.23	0
LMP1	A0A024R4K3	MDH2	46594	46594	46594	1	7700	1	1	1	1	1	6.05	0
LMP1	A8K3H8		29838	29838	29838	1	0	1	1	1	1	1	298380	0
LMP1	Q9UMS4	PRPF19	61198	61198	61198	1	0	1	1	1	1	1	611980	0
LMP1	Q8TBP6	SLC25A40	4463	4463	4463	1	0	1	1	1	1	1	44630	0
LMP1	B2RDH6		35885	35885	35885	1	0	1	1	1	1	1	358850	0
LMP1	Q59FU8		44830	44830	44830	1	0	1	1	1	1	1	448300	0
LMP1	Q8NE86	MCU	56517	56517	56517	1	0	1	1	1	1	1	565170	0
LMP1	Q8IZD4	DCP1B	33275	33275	33275	1	0	1	1	1	1	1	332750	0
LMP1	Q8TEM1	NUP210	18672	18672	18672	1	1990	1	1	1	1	1	9.38	0
LMP1	P61221	ABCE1	34321	34321	34321	1	54	1	1	1	1	1	635.57	0
LMP1	Q9HCC0	MCCC2	62028	62028	62028	1	0	1	1	1	1	1	620280	0
LMP1	A8K2U2		56857	56857	56857	1	0	1	1	1	1	1	568570	0
LMP1	A0A024RDQ8	RFC3	37941	37941	37941	1	0	1	1	1	1	1	379410	0
LMP1	O60759	CYTIP	1693	1693	1693	1	0	1	1	1	1	1	16930	0
LMP1	A8K1F4		60741	60741	60741	1	0	1	1	1	1	1	607410	0
LMP1	Q13422	IKZF1	63783	63783	63783	1	44	1	1	1	1	1	1449.61	0
LMP1	Q9Y5B9	SUPT16H	45532	45532	45532	1	0	1	1	1	1	1	455320	0
LMP1	J3KPS3	ALDOA	42776	42776	42776	1	0	1	1	1	1	1	427760	0
LMP1	Q8WWM7	ATXN2L	65090	65090	65090	1	0	1	1	1	1	1	650900	0
LMP1	P25490	YY1	50408	50408	50408	1	16244	1	1	1	1	1	3.1	0
LMP1	A8K6Q3		42125	42125	42125	1	0	1	1	1	1	1	421250	0
LMP1	P01601	IGKV1D-16	53879	53879	53879	1	1015	1	1	1	1	1	53.08	0
LMP1	Q4LE34	MYO1F variant protein	40450	40450	40450	1	0	1	1	1	1	1	404500	0
LMP1	O15247	CLIC2	30054	30054	30054	1	0	1	1	1	1	1	300540	0
LMP1	B4DUT8	CNN2	24979	24979	24979	1	0	1	1	1	1	1	249790	0
LMP1	A0A024R0G2	DENND2D	21483	21483	21483	1	0	1	1	1	1	1	214830	0
LMP1	Q7RTP6	MICAL3	8099	8099	8099	1	0	1	1	1	1	1	80990	0
LMP1	B3KWQ9		39813	39813	39813	1	1264	1	1	1	1	1	31.5	0
LMP1	Q86U42	PABPN1	52560	52560	52560	1	0	1	1	1	1	1	525600	0
LMP1	P23284	PPIB	45588	45588	45588	1	0	1	1	1	1	1	455880	0
LMP1	B3KMR5		3494	3494	3494	1	0	1	1	1	1	1	34940	0
LMP1	Q96A26	FAM162A	13310	13310	13310	1	0	1	1	1	1	1	133100	0
LMP1	Q9Y4R8	TELO2	63726	63726	63726	1	0	1	1	1	1	1	637260	0
LMP1	Q9Y266	NUDC	6586	6586	6586	1	0	1	1	1	1	1	65860	0
LMP1	Q6ICQ8	ARHG	51179	51179	51179	1	0	1	1	1	1	1	511790	0
LMP1	A0A087WX23	PEG10	6405	6405	6405	1	0	1	1	1	1	1	64050	0
LMP1	P35612	ADD2	42023	42023	42023	1	0	1	1	1	1	1	420230	0
LMP1	A7BI36	RRBP1	6603	6603	6603	1	0	1	1	1	1	1	66030	0
LMP1	P62273	RPS29	7810	7810	7810	1	0	1	1	1	1	1	78100	0
LMP1	A8K319		48750	48750	48750	1	0	1	1	1	1	1	487500	0
LMP1	P13804	ETFA	34809	34809	34809	1	0	1	1	1	1	1	348090	0
LMP1	A3KMH1	VWA8	39112	39112	39112	1	0	1	1	1	1	1	391120	0
LMP1	M0QYZ2	AP2S1	35355	35355	35355	1	0	1	1	1	1	1	353550	0
LMP1	H0YGM0	CLPB	21287	21287	21287	1	0	1	1	1	1	1	212870	0
LMP1	P51159	RAB27A	3633	3633	3633	1	0	1	1	1	1	1	36330	0
LMP1	Q9BT88	SYT11	35316	35316	35316	1	0	1	1	1	1	1	353160	0
LMP1	Q99720	SIGMAR1	46074	46074	46074	1	0	1	1	1	1	1	460740	0
LMP1	P48426	PIP4K2A	8561	8561	8561	1	0	1	1	1	1	1	85610	0
LMP1	Q14683	SMC1A	57938	57938	57938	1	13831	1	1	1	1	1	4.19	0
LMP1	Q15392	DHCR24	36066	36066	36066	1	12024	1	1	1	1	1	3	0
LMP1	P32189	GK	346	346	346	1	0	1	1	1	1	1	3460	0
LMP1	Q01813	PFKP	53582	53582	53582	1	15980	1	1	1	1	1	3.35	0
LMP1	Q9Y5Y5	PEX16	2004	2004	2004	1	0	1	1	1	1	1	20040	0
LMP1	A0A024R8T9	SYNGR2	64718	64718	64718	1	0	1	1	1	1	1	647180	0
LMP1	P82914	MRPS15	14913	14913	14913	1	0	1	1	1	1	1	149130	0
LMP1	P42695	NCAPD3	8920	8920	8920	1	0	1	1	1	1	1	89200	0
LMP1	J3KN16	ECPAS	15634	15634	15634	1	2853	1	1	1	1	1	5.48	0
LMP1	A1L4Q0	DGKE	9495	9495	9495	1	0	1	1	1	1	1	94950	0
LMP1	J9R021	eIF3a	47259	47259	47259	1	0	1	1	1	1	1	472590	0
LMP1	Q8WUM0	NUP133	22010	22010	22010	1	0	1	1	1	1	1	220100	0
LMP1	Q53G26	DNAJA3	44379	44379	44379	1	14444	1	1	1	1	1	3.07	0
LMP1	Q12792	TWF1	50793	50793	50793	1	0	1	1	1	1	1	507930	0
LMP1	O95071	UBR5	31807	31807	31807	1	10059	1	1	1	1	1	3.16	0
LMP1	B2R6L0		13419	13419	13419	1	0	1	1	1	1	1	134190	0
LMP1	Q53HU7		11571	11571	11571	1	0	1	1	1	1	1	115710	0
LMP1	Q9GZP9	DERL2	62165	62165	62165	1	0	1	1	1	1	1	621650	0
LMP1	Q8WU90	ZC3H15	45130	45130	45130	1	0	1	1	1	1	1	451300	0
LMP1	Q5TEU4	NDUFAF5	47446	47446	47446	1	0	1	1	1	1	1	474460	0
LMP1	A8K9U0		29875	29875	29875	1	0	1	1	1	1	1	298750	0
LMP1	J3KSJ5	CYBC1	5211	5211	5211	1	0	1	1	1	1	1	52110	0
LMP1	A0A024R9W5	HUWE1	65335	65335	65335	1	24148	1	1	1	1	1	2.71	0
LMP1	Q9UIU0	CACNA2D1	35888	35888	35888	1	0	1	1	1	1	1	358880	0
LMP1	Q6PGP7	TTC37	62721	62721	62721	1	0	1	1	1	1	1	627210	0
LMP1	Q96AK3	APOBEC3D	12516	12516	12516	1	0	1	1	1	1	1	125160	0
LMP1	B4E1U9		64827	64827	64827	1	20944	1	1	1	1	1	3.1	0
LMP1	P61803	DAD1	25822	25822	25822	1	0	1	1	1	1	1	258220	0
LMP1	Q9BT22	ALG1	18647	18647	18647	1	0	1	1	1	1	1	186470	0
LMP1	O75616	ERAL1	31281	31281	31281	1	0	1	1	1	1	1	312810	0
LMP1	A0A0S2Z4A5	MCM7	34990	34990	34990	1	3042	1	1	1	1	1	11.5	0
LMP1	P51648	ALDH3A2	1400	1400	1400	1	0	1	1	1	1	1	14000	0
LMP1	B7Z6M0		61865	61865	61865	1	6771	1	1	1	1	1	9.14	0
LMP1	Q7Z2E8	VL3L	40264	40264	40264	1	0	1	1	1	1	1	402640	0
LMP1	Q99698	LYST	36274	36274	36274	1	0	1	1	1	1	1	362740	0
LMP1	P26368	U2AF2	22863	22863	22863	1	3800	1	1	1	1	1	6.02	0
LMP1	B2R802		50900	50900	50900	1	0	1	1	1	1	1	509000	0
LMP1	I3L504	EIF5A	18963	18963	18963	1	0	1	1	1	1	1	189630	0
LMP1	Q9ULT0	TTC7A	16430	16430	16430	1	0	1	1	1	1	1	164300	0
LMP1	Q96NB2	SFXN2	60946	60946	60946	1	1385	1	1	1	1	1	44	0
LMP1	Q59GW7		47558	47558	47558	1	0	1	1	1	1	1	475580	0
LMP1	Q92522	H1FX	51575	51575	51575	1	0	1	1	1	1	1	515750	0
LMP1	Q9Y5Z9	UBIAD1	39590	39590	39590	1	0	1	1	1	1	1	395900	0
LMP1	B2RWN5	HEATR1	32006	32006	32006	1	0	1	1	1	1	1	320060	0

LMP1	Q9BUB7	TMEM70	16673	16673	16673	1	0	1	1	1	1	1	166730	0
LMP1	A4D1K4	FAM40B	2463	2463	2463	1	0	1	1	1	1	1	24630	0
LMP1	Q92604	LPGAT1	59424	59424	59424	1	0	1	1	1	1	1	594240	0
LMP1	Q13144	EIF2B5	28729	28729	28729	1	0	1	1	1	1	1	287290	0
LMP1	A0A0B4J207	PRPS1L1	38434	38434	38434	1	0	1	1	1	1	1	384340	0
LMP1	B4E2A6		25532	25532	25532	1	0	1	1	1	1	1	255320	0
LMP1	A8K651		36354	36354	36354	1	3291	1	1	1	1	1	11.05	0
LMP1	Q13489	BIRC3	33122	33122	33122	1	0	1	1	1	1	1	331220	0
LMP1	Q7Z739	YTHDF3	12455	12455	12455	1	0	1	1	1	1	1	124550	0
LMP1	Q16186	ADRM1	26373	26373	26373	1	0	1	1	1	1	1	263730	0
LMP1	P61927	RPL37	21433	21433	21433	1	0	1	1	1	1	1	214330	0
LMP1	A0A1B0GUZ7	EFR3A	19474	19474	19474	1	0	1	1	1	1	1	194740	0
LMP1	Q32Q12	NME1-NME2	49806	49806	49806	1	0	1	1	1	1	1	498060	0
LMP1	P62195	PSMC5	29526	29526	29526	1	4226	1	1	1	1	1	6.99	0
LMP1	G3C9V4	HLA-A	5093	5093	5093	1	0	1	1	1	1	1	50930	0
LMP1	Q9H0F6	SHARPIN	40513	40513	40513	1	0	1	1	1	1	1	405130	0
LMP1	O75643	SNRNP200	33230	33230	33230	1	0	1	1	1	1	1	332300	0
LMP1	Q14694	USP10	3673	3673	3673	1	0	1	1	1	1	1	36730	0
LMP1	B3KS98	EIF3H	14651	14651	14651	1	0	1	1	1	1	1	146510	0
LMP1	A8K245		64404	64404	64404	1	0	1	1	1	1	1	644040	0
LMP1	Q9UN37	VPS4A	61698	61698	61698	1	0	1	1	1	1	1	616980	0
LMP1	Q4W4Y1	DRIP4	44717	44717	44717	1	0	1	1	1	1	1	447170	0
LMP1	O76021	RSL1D1	38950	38950	38950	1	8020	1	1	1	1	1	4.86	0
LMP1	A8K4N4		24096	24096	24096	1	0	1	1	1	1	1	240960	0
LMP1	Q9UPT5	EXOC7	59765	59765	59765	1	0	1	1	1	1	1	597650	0
LMP1	Q9Y2Y4	ZBTB32	57031	57031	57031	1	18762	1	1	1	1	1	3.04	0
LMP1	P35659	DEK	43687	43687	43687	1	0	1	1	1	1	1	436870	0
LMP1	Q5T4S7	UBR4	31938	31938	31938	1	0	1	1	1	1	1	319380	0
LMP1	B2RB23		31544	31544	31544	1	0	1	1	1	1	1	315440	0
LMP1	Q9Y2X9	ZNF281	27401	27401	27401	1	0	1	1	1	1	1	274010	0
LMP1	Q9UKE5	TNIK	17915	17915	17915	1	3733	1	1	1	1	1	4.8	0
LMP1	Q9NPJ8	NXT2	50114	50114	50114	1	3953	1	1	1	1	1	12.68	0
LMP1	P27487	DPP4	17242	17242	17242	1	0	1	1	1	1	1	172420	0
LMP1	P19525	EIF2AK2	58149	58149	58149	1	0	1	1	1	1	1	581490	0
LMP1	Q9P0H9	RER1	52089	52089	52089	1	0	1	1	1	1	1	520890	0
LMP1	E5KLJ5	OPA1	21092	21092	21092	1	2483	1	1	1	1	1	8.49	0
LMP1	A0A024R2L1	WDR48	19395	19395	19395	1	0	1	1	1	1	1	193950	0
LMP1	P08754	GNAI3	53714	53714	53714	1	0	1	1	1	1	1	537140	0
LMP1	O75558	STX11	45131	45131	45131	1	0	1	1	1	1	1	451310	0
LMP1	Q5U0I6	RAB1A	13684	13684	13684	1	0	1	1	1	1	1	136840	0
LMP1	A0A0S2Z404	RCC1	36413	36413	36413	1	0	1	1	1	1	1	364130	0
LMP1	Q96F07	CYFIP2	34821	34821	34821	1	0	1	1	1	1	1	348210	0
LMP1	A0A024QZM0	ASCC1	1958	1958	1958	1	0	1	1	1	1	1	19580	0
LMP1	A0A140EDI1	HLA-DPA1	45660	45660	45660	1	0	1	1	1	1	1	456600	0
LMP1	A0A0S2Z3H6	CLPTM1	22428	22428	22428	1	0	1	1	1	1	1	224280	0
LMP1	A0A024RAH4	RUNX3	44766	44766	44766	1	0	1	1	1	1	1	447660	0
LMP1	Q9HD45	TM9SF3	17719	17719	17719	1	0	1	1	1	1	1	177190	0
LMP1	Q4LE38	IKBKAP variant protein	63456	63456	63456	1	0	1	1	1	1	1	634560	0
LMP1	H7C1Y1	PPP1R21	41884	41884	41884	1	0	1	1	1	1	1	418840	0
LMP1	I3L0L0	RUNX2	24516	24516	24516	1	0	1	1	1	1	1	245160	0
LMP1	P81605	DCD	14376	14376	14376	1	0	1	1	1	1	1	143760	0
LMP1	Q8N6H7	ARFGAP2	18752	18752	18752	1	0	1	1	1	1	1	187520	0
LMP1	V9HWC7	HEL-S-128m	42810	42810	42810	1	11187	1	1	1	1	1	3.83	0
LMP1	Q5UE93	PIK3R6	58429	58429	58429	1	0	1	1	1	1	1	584290	0
LMP1	B3KSR0		25497	25497	25497	1	4322	1	1	1	1	1	5.9	0
LMP1	A0A1B0GVN0	TINCR	48366	48366	48366	1	0	1	1	1	1	1	483660	0
LMP1	Q8IXQ6	PARP9	45489	45489	45489	1	0	1	1	1	1	1	454890	0
LMP1	Q86UT6	NLRX1	18081	18081	18081	1	906	1	1	1	1	1	19.96	0
LMP1	O95470	SGPL1	50624	50624	50624	1	0	1	1	1	1	1	506240	0
LMP1	M0R1Y2	KDELR1	46656	46656	46656	1	0	1	1	1	1	1	466560	0
LMP1	B2R7S3		28496	28496	28496	1	0	1	1	1	1	1	284960	0
LMP1	A0A024RAJ8	IQGAP2	61239	61239	61239	1	0	1	1	1	1	1	612390	0
LMP1	A0A024RAE5	MGC10993	46270	46270	46270	1	6507	1	1	1	1	1	7.11	0
LMP1	Q53GF0		45907	45907	45907	1	0	1	1	1	1	1	459070	0
LMP1	Q9BVS4	RIOK2	22299	22299	22299	1	0	1	1	1	1	1	222990	0
LMP1	G0XQ39	STIM1	60155	60155	60155	1	0	1	1	1	1	1	601550	0
LMP1	O75170	PPP6R2	11436	11436	11436	1	0	1	1	1	1	1	114360	0
LMP1	Q5H9R7	PPP6R3	15659	15659	15659	1	0	1	1	1	1	1	156590	0
LMP1	K7EQW8		52121	52121	52121	1	5718	1	1	1	1	1	9.12	0
LMP1	Q9Y679	AUP1	41659	41659	41659	1	4641	1	1	1	1	1	8.98	0
LMP1	O00231	PSMD11	27017	27017	27017	1	0	1	1	1	1	1	270170	0
LMP1	Q9BWH6	RPAP1	49959	49959	49959	1	0	1	1	1	1	1	499590	0
LMP1	A0A024R755	CALU	44717	44717	44717	1	0	1	1	1	1	1	447170	0
LMP1	A0A0A0MRK6	MTX1	32423	32423	32423	1	0	1	1	1	1	1	324230	0
LMP1	Q6ZT12	UBR3	59671	59671	59671	1	0	1	1	1	1	1	596710	0
LMP1	Q15019	SEPT2	4649	4649	4649	1	0	1	1	1	1	1	46490	0
LMP1	Q9UGR2	ZC3H7B	43805	43805	43805	1	0	1	1	1	1	1	438050	0
LMP1	Q6IAW5	CALU	34289	34289	34289	1	8711	1	1	1	1	1	3.94	0
LMP1	Q5VUA4	ZNF318	25400	25400	25400	1	0	1	1	1	1	1	254000	0
LMP1	E7ERK9	EIF2B4	13418	13418	13418	1	0	1	1	1	1	1	134180	0
LMP1	Q969N2	PIGT	5241	5241	5241	1	0	1	1	1	1	1	52410	0
LMP1	J3KNN5	DDX41	15812	15812	15812	1	0	1	1	1	1	1	158120	0
LMP1	Q8IY17	PNPLA6	33347	33347	33347	1	0	1	1	1	1	1	333470	0
LMP1	P48735	IDH2	10468	10468	10468	1	0	1	1	1	1	1	104680	0
LMP1	Q13148	TARDBP	37308	37308	37308	1	0	1	1	1	1	1	373080	0
LMP1	P36776	LONP1	18167	18167	18167	1	0	1	1	1	1	1	181670	0
LMP1	A0A024R8S5	P4HB	58483	58483	58483	1	0	1	1	1	1	1	584830	0
LMP1	P46013	MKI67	56674	56674	56674	1	0	1	1	1	1	1	566740	0
LMP1	Q9BQ39	DDX50	52207	52207	52207	1	0	1	1	1	1	1	522070	0
LMP1	Q9ULH0	KIDINS220	14431	14431	14431	1	0	1	1	1	1	1	144310	0
LMP1	P09669	COX6C	6079	6079	6079	1	940	1	1	1	1	1	6.47	0
LMP1	Q9NZB2	FAM120A	15099	15099	15099	1	0	1	1	1	1	1	150990	0
LMP1	A8K9K8		52098	52098	52098	1	0	1	1	1	1	1	520980	0
LMP1	A0A024R7X0	ARFGEF1	44125	44125	44125	1	0	1	1	1	1	1	441250	0
LMP1	Q4LE69	PIK4CA variant protein	29684	29684	29684	1	0	1	1	1	1	1	296840	0
LMP1	A0A024R9J0	RAD21	9393	9393	9393	1	0	1	1	1	1	1	93930	0
LMP1	B3KUZ7		49617	49617	49617	1	4732	1	1	1	1	1	10.49	0
LMP1	B3KM95		24052	24052	24052	1	0	1	1	1	1	1	240520	0
LMP1	Q53FR4		48235	48235	48235	1	0	1	1	1	1	1	482350	0
LMP1	P38159	RBMX	34364	34364	34364	1	0	1	1	1	1	1	343640	0

LMP1	Q9NTI5	PDS5B	63762	63762	63762	1	0	1	1	1	1	1	637620	0
LMP1	J3QK89	CHERP	38760	38760	38760	1	0	1	1	1	1	1	387600	0
LMP1	O15160	POLR1C	34542	34542	34542	1	0	1	1	1	1	1	345420	0
LMP1	P52294	KPNA1	55601	55601	55601	1	0	1	1	1	1	1	556010	0
LMP1	A0A024R056	GNB1	39771	39771	39771	1	0	1	1	1	1	1	397710	0
LMP1	A0A024RDE8	PDLIM5	28950	28950	28950	1	0	1	1	1	1	1	289500	0
LMP1	B7Z2P6		25364	25364	25364	1	0	1	1	1	1	1	253640	0
LMP1	A0A0A0MSI8	EXOC5	13340	13340	13340	1	0	1	1	1	1	1	133400	0
LMP1	Q9UPY3	DICER1	50853	50853	50853	1	0	1	1	1	1	1	508530	0
LMP1	Q7Z4H7	HAUS6	47388	47388	47388	1	0	1	1	1	1	1	473880	0
LMP1	P61165	TMEM258	46117	46117	46117	1	0	1	1	1	1	1	461170	0
LMP1	Q15008	PSMD6	34400	34400	34400	1	0	1	1	1	1	1	344000	0
LMP1	Q86X83	COMMD2	25711	25711	25711	1	0	1	1	1	1	1	257110	0
LMP1	B7ZMF2	FANCI	5557	5557	5557	1	0	1	1	1	1	1	55570	0
LMP1	Q13268	DHRS2	28957	28957	28957	1	0	1	1	1	1	1	289570	0
LMP1	B7ZM71	MYO3B	50739	50739	50739	1	0	1	1	1	1	1	507390	0
LMP1	E7EW49	CLASP2	23175	23175	23175	1	0	1	1	1	1	1	231750	0
LMP1	Q9NW64	RBM22	40908	40908	40908	1	0	1	1	1	1	1	409080	0
LMP1	B2R9U2		27648	27648	27648	1	0	1	1	1	1	1	276480	0
LMP1	O14975	SLC27A2	53725	53725	53725	1	0	1	1	1	1	1	537250	0
LMP1	B2R823		14104	14104	14104	1	0	1	1	1	1	1	141040	0
LMP1	Q9ULX6	AKAP8L	9112	9112	9112	1	0	1	1	1	1	1	91120	0
LMP1	X5D907	FMR1	60128	60128	60128	1	0	1	1	1	1	1	601280	0
LMP1	O60645	EXOC3	26336	26336	26336	1	0	1	1	1	1	1	263360	0
LMP1	P53985	SLC16A1	61587	61587	61587	1	12673	1	1	1	1	1	4.86	0
LMP1	Q460N5	PARP14	6085	6085	6085	1	0	1	1	1	1	1	60850	0
LMP1	P57772	EEFSEC	62210	62210	62210	1	0	1	1	1	1	1	622100	0
LMP1	A7E244	TBC1D10A	53759	53759	53759	1	0	1	1	1	1	1	537590	0
LMP1	Q9Y2W1	THRAP3	16620	16620	16620	1	0	1	1	1	1	1	166200	0
LMP1	Q53GS9	USP39	23491	23491	23491	1	0	1	1	1	1	1	234910	0
LMP1	D3DR65	SPFH1	26705	26705	26705	1	0	1	1	1	1	1	267050	0
LMP1	Q9Y4W2	LAS1L	13561	13561	13561	1	0	1	1	1	1	1	135610	0
LMP1	Q08379	GOLGA2	15345	15345	15345	1	0	1	1	1	1	1	153450	0
LMP1	B5BU61	HDAC1	14054	14054	14054	1	0	1	1	1	1	1	140540	0
LMP1	Q9BRJ6	C7orf50	43376	43376	43376	1	0	1	1	1	1	1	433760	0
LMP1	A0A024R203	PSME3	42230	42230	42230	1	0	1	1	1	1	1	422300	0
LMP1	Q9H2M9	RAB3GAP2	2769	2769	2769	1	0	1	1	1	1	1	27690	0
LMP1	Q9ULI1	NWD2	2323	2323	2323	1	0	1	1	1	1	1	23230	0
LMP1	Q6PCD5	RFWD3	53470	53470	53470	1	0	1	1	1	1	1	534700	0
LMP1	P51398	DAP3	33312	33312	33312	1	0	1	1	1	1	1	333120	0
LMP1	B2RBE5		330	330	330	1	0	1	1	1	1	1	3300	0
LMP1	A0PK00	TMEM120B	46339	46339	46339	1	0	1	1	1	1	1	463390	0
LMP1	A6XMH4		39450	39450	39450	1	0	1	1	1	1	1	394500	0
LMP1	O43933	PEX1	32602	32602	32602	1	0	1	1	1	1	1	326020	0
LMP1	B4DJ30		25906	25906	25906	1	0	1	1	1	1	1	259060	0
LMP1	A0A024R0Z3	DDX23	57429	57429	57429	1	0	1	1	1	1	1	574290	0
LMP1	B3KMS0		59292	59292	59292	1	9237	1	1	1	1	1	6.42	0
LMP1	Q6P4A7	SFXN4	54095	54095	54095	1	862	1	1	1	1	1	62.76	0
LMP1	A0A0S2Z556	PQBP1	49157	49157	49157	1	0	1	1	1	1	1	491570	0
LMP1	Q7Z4Q2	HEATR3	38585	38585	38585	1	0	1	1	1	1	1	385850	0
LMP1	P49720	PSMB3	44196	44196	44196	1	0	1	1	1	1	1	441960	0
LMP1	A8K8B2		40590	40590	40590	1	0	1	1	1	1	1	405900	0
LMP1	A0A024R3D8	DLAT	38612	38612	38612	1	0	1	1	1	1	1	386120	0
LMP1	A2A2Q9	AAR2	25769	25769	25769	1	0	1	1	1	1	1	257690	0
LMP1	Q0D2Q6	PGAM1	10225	10225	10225	1	0	1	1	1	1	1	102250	0
LMP1	Q9NR50	EIF2B3	1491	1491	1491	1	0	1	1	1	1	1	14910	0
LMP1	Q9UKZ1	CNOT11	58402	58402	58402	1	0	1	1	1	1	1	584020	0
LMP1	E9PFW3	AP2M1	41476	41476	41476	1	0	1	1	1	1	1	414760	0
LMP1	Q15043	SLC39A14	59467	59467	59467	1	0	1	1	1	1	1	594670	0
LMP1	O94874	UFL1	58244	58244	58244	1	0	1	1	1	1	1	582440	0
LMP1	A0A0A0MT49	SMARCA4	14345	14345	14345	1	0	1	1	1	1	1	143450	0
LMP1	P49792	RANBP2	6705	6705	6705	1	0	1	1	1	1	1	67050	0
LMP1	Q92615	LARP4B	31207	31207	31207	1	0	1	1	1	1	1	312070	0
LMP1	O00487	PSMD14	28560	28560	28560	1	0	1	1	1	1	1	285600	0
LMP1	Q9Y4L1	HYOU1	7200	7200	7200	1	0	1	1	1	1	1	72000	0
LMP1	P25788	PSMA3	5724	5724	5724	1	0	1	1	1	1	1	57240	0
LMP1	O00178	GTPBP1	37203	37203	37203	1	0	1	1	1	1	1	372030	0
LMP1	A6NHB5	ZMYM3	30070	30070	30070	1	0	1	1	1	1	1	300700	0
LMP1	O00411	POLRMT	5518	5518	5518	1	0	1	1	1	1	1	55180	0
LMP1	Q16822	PCK2	60840	60840	60840	1	0	1	1	1	1	1	608400	0
LMP1	B3KPP5		43256	43256	43256	1	0	1	1	1	1	1	432560	0
LMP1	Q7Z3U7	MON2	56471	56471	56471	1	0	1	1	1	1	1	564710	0
LMP1	B7ZVY5	SLFN13	40543	40543	40543	1	0	1	1	1	1	1	405430	0
LMP1	B4DYN5		65403	65403	65403	1	0	1	1	1	1	1	654030	0
LMP1	A8K4V2		50817	50817	50817	1	0	1	1	1	1	1	508170	0
LMP1	A8K1U0		42037	42037	42037	1	0	1	1	1	1	1	420370	0
LMP1	P52434	POLR2H	26085	26085	26085	1	0	1	1	1	1	1	260850	0
LMP1	Q9NX00	TMEM160	15160	15160	15160	1	0	1	1	1	1	1	151600	0
LMP1	Q09666	AHNAK	40350	40350	40350	1	0	1	1	1	1	1	403500	0
LMP1	Q9UFW8	CGGBP1	43542	43542	43542	1	0	1	1	1	1	1	435420	0
LMP1	Q8IY81	FTSJ3	25857	25857	25857	1	0	1	1	1	1	1	258570	0
LMP1	Q9UG56	PISD	14150	14150	14150	1	0	1	1	1	1	1	141500	0
LMP1	B3KSL5		57596	57596	57596	1	0	1	1	1	1	1	575960	0
LMP1	A0A158RFU6	RAB7A	53813	53813	53813	1	0	1	1	1	1	1	538130	0
LMP1	O60341	KDM1A	31179	31179	31179	1	0	1	1	1	1	1	311790	0
LMP1	Q9Y6R9	CCDC61	23056	23056	23056	1	0	1	1	1	1	1	230560	0
LMP1	Q71V34		47029	47029	47029	1	0	1	1	1	1	1	470290	0
LMP1	Q96EP0	RNF31	43097	43097	43097	1	0	1	1	1	1	1	430970	0
LMP1	P42345	MTOR	43036	43036	43036	1	0	1	1	1	1	1	430360	0
LMP1	E7ESZ7	NDUFA10	42656	42656	42656	1	0	1	1	1	1	1	426560	0
LMP1	A5YKK6	CNOT1	29129	29129	29129	1	0	1	1	1	1	1	291290	0
LMP1	Q643R3	LPCAT4	26917	26917	26917	1	0	1	1	1	1	1	269170	0
LMP1	Q9P253	VPS18	54586	54586	54586	1	0	1	1	1	1	1	545860	0
LMP1	Q8WUA4	GTF3C2	49549	49549	49549	1	0	1	1	1	1	1	495490	0
LMP1	Q8IVW6	ARID3B	21723	21723	21723	1	0	1	1	1	1	1	217230	0
LMP1	Q9H2W6	MRPL46	16565	16565	16565	1	0	1	1	1	1	1	165650	0
LMP1	Q9UK61	FAM208A	47364	47364	47364	1	0	1	1	1	1	1	473640	0
LMP1	Q96Q05	TRAPPC9	54186	54186	54186	1	0	1	1	1	1	1	541860	0
LMP1	F5H5R8	NAT1	29289	29289	29289	1	0	1	1	1	1	1	292890	0
LMP1	Q8NC56	LEMD2	6828	6828	6828	1	0	1	1	1	1	1	68280	0

LMP1	A0A0C4DGG9	CHD4	2776	2776	2776	1	0	1	1	1	1	1	27760	0
LMP1	A0A087X2I1	PSMC6	62395	62395	62395	1	0	1	1	1	1	1	623950	0
LMP1	J3KQ48	PTRH2	62245	62245	62245	1	0	1	1	1	1	1	622450	0
LMP1	O43772	SLC25A20	44514	44514	44514	1	0	1	1	1	1	1	445140	0
LMP1	A0A024R8A7		6503	6503	6503	1	0	1	1	1	1	1	65030	0
LMP1	Q16630	CPSF6	4807	4807	4807	1	0	1	1	1	1	1	48070	0
LMP1	O14641	DVL2	58029	58029	58029	1	0	1	1	1	1	1	580290	0
LMP1	B3KMC9		59145	59145	59145	1	0	1	1	1	1	1	591450	0
LMP1	P62633	CNBP	57052	57052	57052	1	0	1	1	1	1	1	570520	0
LMP1	Q4LE64	NUMA1 variant protein	54458	54458	54458	1	0	1	1	1	1	1	544580	0
LMP1	A0A024R8B6	NUP214	22907	22907	22907	1	0	1	1	1	1	1	229070	0
LMP1	Q96EE3	SEH1L	15879	15879	15879	1	0	1	1	1	1	1	158790	0
LMP1	A0A024RAE1	C1orf33	53759	53759	53759	1	0	1	1	1	1	1	537590	0
LMP1	Q8ND56	LSM14A	32831	32831	32831	1	0	1	1	1	1	1	328310	0
LMP1	Q14CZ7	FASTKD3	26786	26786	26786	1	0	1	1	1	1	1	267860	0
LMP1	Q9NZJ4	SACS	62613	62613	62613	1	0	1	1	1	1	1	626130	0
LMP1	P29597	TYK2	37922	37922	37922	1	0	1	1	1	1	1	379220	0
LMP1	B2R8H0		43188	43188	43188	1	0	1	1	1	1	1	431880	0
LMP1	Q03701	CEBPZ	25229	25229	25229	1	0	1	1	1	1	1	252290	0
LMP1	Q9NZJ0	DTL	5010	5010	5010	1	0	1	1	1	1	1	50100	0
LMP1	A0A0A0MT22	PTPRC	14876	14876	14876	1	0	1	1	1	1	1	148760	0
LMP1	G3V5Z7	PSMA6	25202	25202	25202	1	0	1	1	1	1	1	252020	0
LMP1	O94804	STK10	7471	7471	7471	1	0	1	1	1	1	1	74710	0
LMP1	Q9BQA1	WDR77	49638	49638	49638	1	0	1	1	1	1	1	496380	0
LMP1	A0A068F7M9	FHOD1	23910	23910	23910	1	0	1	1	1	1	1	239100	0
LMP1	A0A087X1Z3	PSME2	9667	9667	9667	1	0	1	1	1	1	1	96670	0
LMP1	A0A024R1X8	JUP	58114	58114	58114	1	20776	1	1	1	1	1	2.8	0
LMP1	O75843	AP1G2	52619	52619	52619	1	0	1	1	1	1	1	526190	0
LMP1	O75179	ANKRD17	17360	17360	17360	1	0	1	1	1	1	1	173600	0
LMP1	A0A024R7N4	RGS14	6978	6978	6978	1	0	1	1	1	1	1	69780	0
LMP1	O00186	STXBP3	35726	35726	35726	1	0	1	1	1	1	1	357260	0
LMP1	A0A024R2M7	OXSR1	18493	18493	18493	1	0	1	1	1	1	1	184930	0
LMP1	Q9BYG3	NIFK	17971	17971	17971	1	0	1	1	1	1	1	179710	0
LMP1	P40306	PSMB10	57528	57528	57528	1	0	1	1	1	1	1	575280	0
LMP1	Q58EX7	PLEKHG4	30950	30950	30950	1	0	1	1	1	1	1	309500	0
LMP1	A0A024R7U6	MCM4	11427	11427	11427	1	0	1	1	1	1	1	114270	0
LMP1	Q13895	BYSL	5867	5867	5867	1	0	1	1	1	1	1	58670	0
LMP1	Q96QU8	XPO6	44350	44350	44350	1	0	1	1	1	1	1	443500	0
LMP1	A8KAP3		18552	18552	18552	1	0	1	1	1	1	1	185520	0
LMP1	B3KWB6		58112	58112	58112	1	0	1	1	1	1	1	581120	0
LMP1	O43684	BUB3	24985	24985	24985	1	0	1	1	1	1	1	249850	0
LMP1	B2RB13		16204	16204	16204	1	0	1	1	1	1	1	162040	0
LMP1	Q96JJ7	TMX3	6243	6243	6243	1	0	1	1	1	1	1	62430	0
LMP1	Q9BRZ2	TRIM56	62034	62034	62034	1	0	1	1	1	1	1	620340	0
LMP1	B2R673		58753	58753	58753	1	0	1	1	1	1	1	587530	0
LMP1	Q9UBF2	COPG2	45357	45357	45357	1	0	1	1	1	1	1	453570	0
LMP1	Q3KQU3	MAP7D1	38410	38410	38410	1	0	1	1	1	1	1	384100	0
LMP1	Q9UK59	DBR1	22218	22218	22218	1	0	1	1	1	1	1	222180	0
LMP1	Q08945	SSRP1	4728	4728	4728	1	0	1	1	1	1	1	47280	0
LMP1	Q9H0A0	NAT10	55700	55700	55700	1	0	1	1	1	1	1	557000	0
LMP1	A0A024RDR0	HMGB1	32002	32002	32002	1	0	1	1	1	1	1	320020	0
LMP1	Q12981	BNIP1	7277	7277	7277	1	0	1	1	1	1	1	72770	0
LMP1	O94906	PRPF6	405	405	405	1	0	1	1	1	1	1	4050	0
LMP1	Q8IUF8	RIOX2	50423	50423	50423	1	0	1	1	1	1	1	504230	0
LMP1	Q53F02		51789	51789	51789	1	0	1	1	1	1	1	517890	0
LMP1	Q14139	UBE4A	43665	43665	43665	1	0	1	1	1	1	1	436650	0
LMP1	Q9Y305	ACOT9	56974	56974	56974	1	0	1	1	1	1	1	569740	0
LMP1	B4DYZ7		33716	33716	33716	1	0	1	1	1	1	1	337160	0
LMP1	P08240	SRPRA	21592	21592	21592	1	0	1	1	1	1	1	215920	0
LMP1	Q8WXW3	PIBF1	15516	15516	15516	1	0	1	1	1	1	1	155160	0
LMP1	A0A024QYX0	EBP	5280	5280	5280	1	0	1	1	1	1	1	52800	0
LMP1	Q92878	RAD50	43931	43931	43931	1	0	1	1	1	1	1	439310	0
LMP1	Q15286	RAB35	24424	24424	24424	1	0	1	1	1	1	1	244240	0
LMP1	B8ZZD4	TAX1BP1	49218	49218	49218	1	0	1	1	1	1	1	492180	0
LMP1	Q9P2K3	RCOR3	48815	48815	48815	1	0	1	1	1	1	1	488150	0
LMP1	P82650	MRPS22	48981	48981	48981	1	0	1	1	1	1	1	489810	0
LMP1	A0A024R9L1	AQR	48298	48298	48298	1	0	1	1	1	1	1	482980	0
LMP1	P11177	PDHB	52886	52886	52886	1	0	1	1	1	1	1	528860	0
LMP1	A0A024R0F1	TTF2	58366	58366	58366	1	0	1	1	1	1	1	583660	0
LMP1	O14874	BCKDK	46910	46910	46910	1	0	1	1	1	1	1	469100	0
LMP1	O95782	AP2A1	14052	14052	14052	1	0	1	1	1	1	1	140520	0
LMP1	J3KRY1	ARHGDIA	34476	34476	34476	1	0	1	1	1	1	1	344760	0
LMP1	Q96EK4	THAP11	21554	21554	21554	1	0	1	1	1	1	1	215540	0
LMP1	J3KNL2	SEPT1	15696	15696	15696	1	0	1	1	1	1	1	156960	0
LMP1	O94768	STK17B	49767	49767	49767	1	0	1	1	1	1	1	497670	0
LMP1	A0A1C9J6R2		38632	38632	38632	1	0	1	1	1	1	1	386320	0
LMP1	A0A0S2Z500	OPTN	28883	28883	28883	1	0	1	1	1	1	1	288830	0
LMP1	H3BQK9	MACF1	16826	16826	16826	1	0	1	1	1	1	1	168260	0
LMP1	Q13535	ATR	2935	2935	2935	1	0	1	1	1	1	1	29350	0
LMP1	Q9Y2W2	WBP11	15523	15523	15523	1	0	1	1	1	1	1	155230	0
LMP1	A0A024R3M1	THYN1	3606	3606	3606	1	0	1	1	1	1	1	36060	0
LMP1	Q59F14		34558	34558	34558	1	0	1	1	1	1	1	345580	0
LMP1	A0A090J7P6	BIVM-ERCC5	61453	61453	61453	1	0	1	1	1	1	1	614530	0
LMP1	P22079	LPO	16491	16491	16491	1	0	1	1	1	1	1	164910	0
LMP1	P23368	ME2	8745	8745	8745	1	0	1	1	1	1	1	87450	0
LMP1	Q6P4F7	ARHGAP11A	8211	8211	8211	1	0	1	1	1	1	1	82110	0
LMP1	Q92905	COPS5	21336	21336	21336	1	0	1	1	1	1	1	213360	0
LMP1	Q9H9T3	ELP3	37618	37618	37618	1	0	1	1	1	1	1	376180	0
LMP1	Q5TA45	INTS11	59842	59842	59842	1	0	1	1	1	1	1	598420	0
LMP1	Q5EFE6		34428	34428	34428	1	3907	1	1	1	1	1	8.81	0
LMP1	O43242	PSMD3	51556	51556	51556	1	0	1	1	1	1	1	515560	0
LMP1	Q53HS1		36074	36074	36074	1	0	1	1	1	1	1	360740	0
LMP1	A0A087WTP3	KHSRP	26932	26932	26932	1	0	1	1	1	1	1	269320	0
LMP1	Q8WV92	MITD1	20323	20323	20323	1	0	1	1	1	1	1	203230	0
LMP1	Q14247	CTTN	15346	15346	15346	1	0	1	1	1	1	1	153460	0
LMP1	P15924	DSP	63805	63805	63805	1	17822	1	1	1	1	1	3.58	0
LMP1	B1PBA3	SMPD4	25401	25401	25401	1	0	1	1	1	1	1	254010	0
LMP1	Q5T9A4	ATAD3B	41285	41285	41285	1	0	1	1	1	1	1	412850	0
LMP1	A8K9U1		15503	15503	15503	1	0	1	1	1	1	1	155030	0
LMP1	Q07011	TNFRSF9	54151	54151	54151	1	0	1	1	1	1	1	541510	0

LMP1	Q8NBJ5	COLGALT1	62292	62292	62292	1	0	1	1	1	1	1	622920	0
LMP1	R4GMX3	COMMD3-BMI1	9787	9787	9787	1	0	1	1	1	1	1	97870	0
LMP1	O43776	NARS	18359	18359	18359	1	0	1	1	1	1	1	183590	0
LMP1	Q6P1J9	CDC73	14825	14825	14825	1	0	1	1	1	1	1	148250	0
LMP1	Q5T0F3	GNL2	626	626	626	1	0	1	1	1	1	1	6260	0
LMP1	Q9H900	ZWILCH	37724	37724	37724	1	0	1	1	1	1	1	377240	0
LMP1	Q8TDD1	DDX54	46626	46626	46626	1	0	1	1	1	1	1	466260	0
LMP1	O43808	SLC25A17	15040	15040	15040	1	0	1	1	1	1	1	150400	0
LMP1	A4D218	MAD1L1	52852	52852	52852	1	0	1	1	1	1	1	528520	0
LMP1	Q53G47		34832	34832	34832	1	0	1	1	1	1	1	348320	0
LMP1	E5KN59		55002	55002	55002	1	0	1	1	1	1	1	550020	0
LMP1	B3KWH9	ELOVL5	46545	46545	46545	1	696	1	1	1	1	1	66.88	0
LMP1	Q6PJG6	BRAT1	37085	37085	37085	1	7536	1	1	1	1	1	4.92	0
LMP1	H0Y2S9	MPRIP	32226	32226	32226	1	0	1	1	1	1	1	322260	0
LMP1	O94915	FRYL	13351	13351	13351	1	0	1	1	1	1	1	133510	0
LMP1	B9A6K8	TBC1D9B	51191	51191	51191	1	0	1	1	1	1	1	511910	0
LMP1	P28838	LAP3	30001	30001	30001	1	0	1	1	1	1	1	300010	0
LMP1	P55010	EIF5	32866	32866	32866	1	0	1	1	1	1	1	328660	0
LMP1	Q86VV8	RTTN	24748	24748	24748	1	0	1	1	1	1	1	247480	0
LMP1	Q6P9B9	INTS5	30912	30912	30912	1	0	1	1	1	1	1	309120	0
LMP1	M0QZR4	ARHGEF1	24983	24983	24983	1	0	1	1	1	1	1	249830	0
LMP1	A0A0S2Z392	ADRBK1	58456	58456	58456	1	0	1	1	1	1	1	584560	0
LMP1	O75317	USP12	28596	28596	28596	1	0	1	1	1	1	1	285960	0
LMP1	Q99816	TSG101	569	569	569	1	0	1	1	1	1	1	5690	0
LMP1	Q9NUD5	ZCCHC3	48407	48407	48407	1	0	1	1	1	1	1	484070	0
LMP1	A0A087WUB9	CTNNBL1	7636	7636	7636	1	0	1	1	1	1	1	76360	0
LMP1	Q8N2K0	ABHD12	24562	24562	24562	1	0	1	1	1	1	1	245620	0
LMP1	Q5SRE5	NUP188	450	450	450	1	0	1	1	1	1	1	4500	0
LMP1	O43572	AKAP10	50762	50762	50762	1	0	1	1	1	1	1	507620	0
LMP1	Q14643	ITPR1	35403	35403	35403	1	0	1	1	1	1	1	354030	0
LMP1	Q9H4L5	OSBPL3	10035	10035	10035	1	0	1	1	1	1	1	100350	0
LMP1	Q96AA3	RFT1	26804	26804	26804	1	0	1	1	1	1	1	268040	0
LMP1	Q9BW27	NUP85	24030	24030	24030	1	0	1	1	1	1	1	240300	0
LMP1	Q9NVR2	INTS10	1810	1810	1810	1	0	1	1	1	1	1	18100	0
LMP1	P35579	MYH9	0	0	0	1	0	0	0	0	0	0	0	0
LMP1	A0A125QYY8		0	0	0	1	0	0	0	0	0	0	0	0
LMP1	A0A0S2Z4G8	TPM3	0	0	0	1	0	0	0	0	0	0	0	0
LMP1	A0A087WYE8	IGHV1OR21-1	0	0	0	1	0	0	0	0	0	0	0	0
LMP1	G8JLA2	MYL6	0	0	0	1	32670	0	0	0	0	0	0	0
LMP1	J3QRS3	MYL12A	0	0	0	1	16782	0	0	0	0	0	0	0
LMP1	Q7Z406	MYH14	0	0	0	1	18149	0	0	0	0	0	0	0
LMP1	S6BGE0		0	0	0	1	42945	0	0	0	0	0	0	0
LMP1	B0I1T2	MYO1G	0	0	0	1	20548	0	0	0	0	0	0	0
LMP1	P68104	EEF1A1	0	0	0	1	51886	0	0	0	0	0	0	0
LMP1	P33241	LSP1	0	0	0	1	39493	0	0	0	0	0	0	0
LMP1	J3KN67	TPM3	0	0	0	1	0	0	0	0	0	0	0	0
LMP1	H6VRG1	KRT1	51350	51350	51350	1	29740	0	0	0	0	0	1.73	0
LMP1	P07437	TUBB	45498	45498	45498	1	28437	0	0	0	0	0	1.6	0
LMP1	B4DW52		38397	38397	38397	1	20207	0	0	0	0	0	1.9	0
LMP1	Q6ZNK5	FLJ00293	9282	9282	9282	1	51816	0	0	0	0	0	0.18	0
LMP1	P05141	SLC25A5	60683	60683	60683	1	47013	0	0	0	0	0	1.29	0
LMP1	A0N5G3	V-lambda-3	61126	61126	61126	1	40466	0	0	0	0	0	1.51	0
LMP1	F5H5D3	TUBA1C	60196	60196	60196	1	30872	0	0	0	0	0	1.95	0
LMP1	P62917	RPL8	55878	55878	55878	1	25354	0	0	0	0	0	2.2	0
LMP1	O43707	ACTN4	61345	61345	61345	1	62862	0	0	0	0	0	0.98	0
LMP1	A2NJV5	IGKV2-29	26630	26630	26630	1	16433	0	0	0	0	0	1.62	0
LMP1	P19474	TRIM21	39117	39117	39117	1	33937	0	0	0	0	0	1.15	0
LMP1	Q13707	ACTA2	37211	37211	37211	1	48408	0	0	0	0	0	0.77	0
LMP1	V9HWE1	HEL113	2703	2703	2703	1	51816	0	0	0	0	0	0.05	0
LMP1	V9HW22	HEL-S-72p	31775	31775	31775	1	63610	0	0	0	0	0	0.5	0
LMP1	Q00325	SLC25A3	57953	57953	57953	1	26282	0	0	0	0	0	2.21	0
LMP1	A0A0A0MRZ8	IGKV3D-11	56680	56680	56680	1	64414	0	0	0	0	0	0.88	0
LMP1	B2R7T8		33016	33016	33016	1	64768	0	0	0	0	0	0.51	0
LMP1	P26373	RPL13	18255	18255	18255	1	49827	0	0	0	0	0	0.37	0
LMP1	P52907	CAPZA1	47738	47738	47738	1	48293	0	0	0	0	0	0.99	0
LMP1	Q6IPH7	RPL14	12976	12976	12976	1	29388	0	0	0	0	0	0.44	0
LMP1	P62906	RPL10A	60293	60293	60293	1	50587	0	0	0	0	0	1.19	0
LMP1	P18124	RPL7	31317	31317	31317	1	58355	0	0	0	0	0	0.54	0
LMP1	P67936	TPM4	8247	8247	8247	1	28655	0	0	0	0	0	0.29	0
LMP1	P39023	RPL3	60306	60306	60306	1	32035	0	0	0	0	0	1.88	0
LMP1	Q5HYL6	DKFZp686E1899	27577	27577	27577	1	39084	0	0	0	0	0	0.71	0
LMP1	J3QQ67	RPL18	6086	6086	6086	1	50721	0	0	0	0	0	0.12	0
LMP1	P27635	RPL10	12461	12461	12461	1	19877	0	0	0	0	0	0.63	0
LMP1	P62424	RPL7A	13382	13382	13382	1	61853	0	0	0	0	0	0.22	0
LMP1	Q9Y509	VH3	39562	39562	39562	1	30349	0	0	0	0	0	1.3	0
LMP1	P21796	VDAC1	45762	45762	45762	1	47662	0	0	0	0	0	0.96	0
LMP1	P84098	RPL19	15263	15263	15263	1	10477	0	0	0	0	0	1.46	0
LMP1	Q9HBB3		31684	31684	31684	1	21361	0	0	0	0	0	1.48	0
LMP1	Q9UL88		51765	51765	51765	1	53841	0	0	0	0	0	0.96	0
LMP1	A4QPB0	IQGAP1	23089	23089	23089	1	47510	0	0	0	0	0	0.49	0
LMP1	P62913	RPL11	35166	35166	35166	1	22433	0	0	0	0	0	1.57	0
LMP1	B2RDW0		23001	23001	23001	1	46650	0	0	0	0	0	0.49	0
LMP1	P62263	RPS14	32463	32463	32463	1	52087	0	0	0	0	0	0.62	0
LMP1	P62701	RPS4X	34087	34087	34087	1	61282	0	0	0	0	0	0.56	0
LMP1	M0QYS1	RPL13A	5985	5985	5985	1	55445	0	0	0	0	0	0.11	0
LMP1	Q96C19	EFHD2	34552	34552	34552	1	29069	0	0	0	0	0	1.19	0
LMP1	P30050	RPL12	8071	8071	8071	1	13213	0	0	0	0	0	0.61	0
LMP1	P62979	RPS27A	43382	43382	43382	1	52781	0	0	0	0	0	0.82	0
LMP1	A0A024R1V4	RPL27	56179	56179	56179	1	50307	0	0	0	0	0	1.12	0
LMP1	P62269	RPS18	27938	27938	27938	1	51384	0	0	0	0	0	0.54	0
LMP1	A0N5G1	V-kappa-1	37473	37473	37473	1	57977	0	0	0	0	0	0.65	0
LMP1	P35580	MYH10	15064	15064	15064	1	15084	0	0	0	0	0	1	0
LMP1	B7Z4C8	RPL31	1173	1173	1173	1	3862	0	0	0	0	0	0.3	0
LMP1	A0A024R2Q4	RPL15	7826	7826	7826	1	18238	0	0	0	0	0	0.43	0
LMP1	Q7Z351	DKFZp686N02209	25413	25413	25413	1	18816	0	0	0	0	0	1.35	0
LMP1	P62280	RPS11	11443	11443	11443	1	30666	0	0	0	0	0	0.37	0
LMP1	P62081	RPS7	4412	4412	4412	1	34639	0	0	0	0	0	0.13	0
LMP1	P46776	RPL27A	26457	26457	26457	1	32133	0	0	0	0	0	0.82	0
LMP1	A2KBC6		56736	56736	56736	1	30681	0	0	0	0	0	1.85	0
LMP1	Q16891	IMMT	38089	38089	38089	1	59985	0	0	0	0	0	0.63	0

LMP1	A0A0X9UWL5		15620	15620	15620	1	28522	0	0	0	0	0	0.55	0
LMP1	A0A024QZN9	VDAC2	14611	14611	14611	1	37552	0	0	0	0	0	0.39	0
LMP1	B3KMV8		42551	42551	42551	1	47106	0	0	0	0	0	0.9	0
LMP1	Q5JR94	RPS8	23159	23159	23159	1	63531	0	0	0	0	0	0.36	0
LMP1	Q6IAX2	RPL21	56015	56015	56015	1	53696	0	0	0	0	0	1.04	0
LMP1	Q14764	MVP	45066	45066	45066	1	43655	0	0	0	0	0	1.03	0
LMP1	P23396	RPS3	30460	30460	30460	1	55189	0	0	0	0	0	0.55	0
LMP1	A0A075B6K5	IGLV3-9	49455	49455	49455	1	48852	0	0	0	0	0	1.01	0
LMP1	P38646	HSPA9	37387	37387	37387	1	51817	0	0	0	0	0	0.72	0
LMP1	P15880	RPS2	16138	16138	16138	1	13212	0	0	0	0	0	1.22	0
LMP1	A0A024R4Q8	RPS5	3350	3350	3350	1	10501	0	0	0	0	0	0.32	0
LMP1	Q13077	TRAF1	45579	45579	45579	1	21302	0	0	0	0	0	2.14	0
LMP1	P27708	CAD	33651	33651	33651	1	45224	0	0	0	0	0	0.74	0
LMP1	A0A125QYY9		17360	17360	17360	1	8523	0	0	0	0	0	2.04	0
LMP1	A2J1M8		16815	16815	16815	1	7192	0	0	0	0	0	2.34	0
LMP1	A0A0F7TAV4	IGHV5-51	45009	45009	45009	1	54482	0	0	0	0	0	0.83	0
LMP1	Q5VV89	MGST3	42782	42782	42782	1	48580	0	0	0	0	0	0.88	0
LMP1	P68371	TUBB4B	61073	61073	61073	1	51173	0	0	0	0	0	1.19	0
LMP1	V9HW26	HEL-S-123m	24430	24430	24430	1	35582	0	0	0	0	0	0.69	0
LMP1	P62854	RPS26	27554	27554	27554	1	43273	0	0	0	0	0	0.64	0
LMP1	Q2M272	PCDHGC5	46425	46425	46425	1	44840	0	0	0	0	0	1.04	0
LMP1	P62851	RPS25	61647	61647	61647	1	27107	0	0	0	0	0	2.27	0
LMP1	P61254	RPL26	1794	1794	1794	1	36809	0	0	0	0	0	0.05	0
LMP1	H3BNS8	USP10	18511	18511	18511	1	50719	0	0	0	0	0	0.36	0
LMP1	P46781	RPS9	13246	13246	13246	1	34745	0	0	0	0	0	0.38	0
LMP1	A0A068LN03		17894	17894	17894	1	59434	0	0	0	0	0	0.3	0
LMP1	P62888	RPL30	13165	13165	13165	1	25274	0	0	0	0	0	0.52	0
LMP1	V9HW31	HEL-S-271	54657	54657	54657	1	51091	0	0	0	0	0	1.07	0
LMP1	B4DR52		58745	58745	58745	1	51797	0	0	0	0	0	1.13	0
LMP1	P49207	RPL34	41453	41453	41453	1	19316	0	0	0	0	0	2.15	0
LMP1	P83731	RPL24	22176	22176	22176	1	32770	0	0	0	0	0	0.68	0
LMP1	P10809	HSPD1	39059	39059	39059	1	52320	0	0	0	0	0	0.75	0
LMP1	P21333	FLNA	22273	22273	22273	1	29789	0	0	0	0	0	0.75	0
LMP1	A0A024R1Q8	RPL23	27659	27659	27659	1	27585	0	0	0	0	0	1	0
LMP1	A0A0U1RRH7		8205	8205	8205	1	36927	0	0	0	0	0	0.22	0
LMP1	Q6I9V5	SLC25A6	48533	48533	48533	1	49879	0	0	0	0	0	0.97	0
LMP1	A0A0A6YYL6	RPL17-C18orf32	20756	20756	20756	1	52586	0	0	0	0	0	0.39	0
LMP1	P18077	RPL35A	29606	29606	29606	1	62346	0	0	0	0	0	0.47	0
LMP1	Q9UL83		45708	45708	45708	1	24743	0	0	0	0	0	1.85	0
LMP1	A0A024R7I5	TRMT1	7112	7112	7112	1	25789	0	0	0	0	0	0.28	0
LMP1	B1N7B6		50217	50217	50217	1	57827	0	0	0	0	0	0.87	0
LMP1	P35268	RPL22	3921	3921	3921	1	25276	0	0	0	0	0	0.16	0
LMP1	P46779	RPL28	16707	16707	16707	1	21111	0	0	0	0	0	0.79	0
LMP1	C9JRZ6	CHCHD3	55248	55248	55248	1	34333	0	0	0	0	0	1.61	0
LMP1	A2A3R6	RPS6	770	770	770	1	8206	0	0	0	0	0	0.09	0
LMP1	P35908	KRT2	17880	17880	17880	1	37632	0	0	0	0	0	0.48	0
LMP1	Q5NV69	V1-13	8956	8956	8956	1	4197	0	0	0	0	0	2.13	0
LMP1	A0A0A0MSV9	TAPBP	62027	62027	62027	1	46117	0	0	0	0	0	1.34	0
LMP1	Q9UL89		13489	13489	13489	1	33499	0	0	0	0	0	0.4	0
LMP1	G1EPU0	HLA-B	51689	51689	51689	1	33815	0	0	0	0	0	1.53	0
LMP1	Q8WVX7		23413	23413	23413	1	17470	0	0	0	0	0	1.34	0
LMP1	Q53Z07	RPL9	56560	56560	56560	1	64413	0	0	0	0	0	0.88	0
LMP1	Q5NV90	V2-17	46033	46033	46033	1	49355	0	0	0	0	0	0.93	0
LMP1	Q92608	DOCK2	48502	48502	48502	1	45855	0	0	0	0	0	1.06	0
LMP1	P05387	RPLP2	37481	37481	37481	1	21746	0	0	0	0	0	1.72	0
LMP1	P78527	PRKDC	3373	3373	3373	1	2839	0	0	0	0	0	1.19	0
LMP1	A4D0V4	CAPZA2	16307	16307	16307	1	52042	0	0	0	0	0	0.31	0
LMP1	A0A0C4DH35	IGHV3-35	62136	62136	62136	1	38617	0	0	0	0	0	1.61	0
LMP1	Q9UL82		6787	6787	6787	1	60088	0	0	0	0	0	0.11	0
LMP1	Q92614	MYO18A	43662	43662	43662	1	61127	0	0	0	0	0	0.71	0
LMP1	A0A109PSY4		16836	16836	16836	1	11377	0	0	0	0	0	1.48	0
LMP1	Q5NV88	V1-22	34720	34720	34720	1	37994	0	0	0	0	0	0.91	0
LMP1	A0A0X9T7V9		26064	26064	26064	1	57401	0	0	0	0	0	0.45	0
LMP1	P08238	HSP90AB1	9266	9266	9266	1	5569	0	0	0	0	0	1.66	0
LMP1	Q0ZCF6		1642	1642	1642	1	8988	0	0	0	0	0	0.18	0
LMP1	B4E0T8		62332	62332	62332	1	52228	0	0	0	0	0	1.19	0
LMP1	A0A075B6H6	IGKC	26682	26682	26682	1	19458	0	0	0	0	0	1.37	0
LMP1	Q00839	HNRNPU	10584	10584	10584	1	50125	0	0	0	0	0	0.21	0
LMP1	A8K517	RPS23	41036	41036	41036	1	28945	0	0	0	0	0	1.42	0
LMP1	A0A068LKQ2		2350	2350	2350	1	53581	0	0	0	0	0	0.04	0
LMP1	H0Y2W2	ATAD3A	43942	43942	43942	1	50875	0	0	0	0	0	0.86	0
LMP1	Q96A19	CCDC102A	1059	1059	1059	1	31788	0	0	0	0	0	0.03	0
LMP1	Q9Y277	VDAC3	1540	1540	1540	1	20883	0	0	0	0	0	0.07	0
LMP1	P60866	RPS20	48243	48243	48243	1	26459	0	0	0	0	0	1.82	0
LMP1	P11940	PABPC1	13395	13395	13395	1	59105	0	0	0	0	0	0.23	0
LMP1	Q8NCL6		27856	27856	27856	1	21180	0	0	0	0	0	1.32	0
LMP1	K7ES00	H3F3B	986	986	986	1	60324	0	0	0	0	0	0.02	0
LMP1	C8C504	HBB	42297	42297	42297	1	49000	0	0	0	0	0	0.86	0
LMP1	A0A024R1N4	XRCC6	22756	22756	22756	1	44197	0	0	0	0	0	0.51	0
LMP1	A2J1N6		44813	44813	44813	1	52546	0	0	0	0	0	0.85	0
LMP1	Q9UKK3	PARP4	51814	51814	51814	1	64745	0	0	0	0	0	0.8	0
LMP1	E9PBG7	CAMK2D	1530	1530	1530	1	14807	0	0	0	0	0	0.1	0
LMP1	P08708	RPS17	25319	25319	25319	1	48825	0	0	0	0	0	0.52	0
LMP1	Q9HCC1		50171	50171	50171	1	26161	0	0	0	0	0	1.92	0
LMP1	A0A0X9V9B3		530	530	530	1	64192	0	0	0	0	0	0.01	0
LMP1	Q9Y3U8	RPL36	49488	49488	49488	1	20359	0	0	0	0	0	2.43	0
LMP1	Q9UE89		10479	10479	10479	1	37497	0	0	0	0	0	0.28	0
LMP1	Q8NEJ1		58873	58873	58873	1	44959	0	0	0	0	0	1.31	0
LMP1	F8W727	RPL32	57370	57370	57370	1	62326	0	0	0	0	0	0.92	0
LMP1	A0N5G7	VH3	9250	9250	9250	1	46920	0	0	0	0	0	0.2	0
LMP1	A0A125QYY5		62337	62337	62337	1	34769	0	0	0	0	0	1.79	0
LMP1	B2RBR9		1818	1818	1818	1	21516	0	0	0	0	0	0.08	0
LMP1	B2R5W3		29585	29585	29585	1	45529	0	0	0	0	0	0.65	0
LMP1	P13647	KRT5	26474	26474	26474	1	29746	0	0	0	0	0	0.89	0
LMP1	E1NZA1	PRIC295	3708	3708	3708	1	53448	0	0	0	0	0	0.07	0
LMP1	P42677	RPS27	23572	23572	23572	1	63149	0	0	0	0	0	0.37	0
LMP1	A2J1N5		25891	25891	25891	1	54424	0	0	0	0	0	0.48	0
LMP1	K7ELC2	RPS15	43100	43100	43100	1	31545	0	0	0	0	0	1.37	0
LMP1	A0A087WVQ6	CLTC	937	937	937	1	24547	0	0	0	0	0	0.04	0
LMP1	P23246	SFPQ	44665	44665	44665	1	60099	0	0	0	0	0	0.74	0

LMP1	E9PAV3	NACA	40902	40902	40902	1	48011	0	0	0	0	0	0.85	0
LMP1	P46783	RPS10	32226	32226	32226	1	53874	0	0	0	0	0	0.6	0
LMP1	P49411	TUFM	29407	29407	29407	1	20059	0	0	0	0	0	1.47	0
LMP1	P61160	ACTR2	50061	50061	50061	1	37342	0	0	0	0	0	1.34	0
LMP1	B9EIS5	PCM1	9753	9753	9753	1	41100	0	0	0	0	0	0.24	0
LMP1	B2DG08	HLA-B	49798	49798	49798	1	27073	0	0	0	0	0	1.84	0
LMP1	B3KUE6		6636	6636	6636	1	17366	0	0	0	0	0	0.38	0
LMP1	A0A0U1RRM4	PTBP1	22057	22057	22057	1	11594	0	0	0	0	0	1.9	0
LMP1	Q9H9B4	SFXN1	62595	62595	62595	1	37396	0	0	0	0	0	1.67	0
LMP1	Q67BC3		59527	59527	59527	1	48824	0	0	0	0	0	1.22	0
LMP1	Q562M5	ACT	37343	37343	37343	1	42497	0	0	0	0	0	0.88	0
LMP1	A2NW97		16723	16723	16723	1	21139	0	0	0	0	0	0.79	0
LMP1	P02545	LMNA	16253	16253	16253	1	31464	0	0	0	0	0	0.52	0
LMP1	P67809	YBX1	50080	50080	50080	1	63006	0	0	0	0	0	0.79	0
LMP1	P04003	C4BPA	40353	40353	40353	1	40191	0	0	0	0	0	1	0
LMP1	Q59G24		63246	63246	63246	1	25740	0	0	0	0	0	2.46	0
LMP1	Q13045	FLII	45774	45774	45774	1	35695	0	0	0	0	0	1.28	0
LMP1	P13639	EEF2	10165	10165	10165	1	25139	0	0	0	0	0	0.4	0
LMP1	Q53HQ0		26451	26451	26451	1	40428	0	0	0	0	0	0.65	0
LMP1	P22626	HNRNPA2B1	21708	21708	21708	1	17292	0	0	0	0	0	1.26	0
LMP1	Q9Y230	RUVBL2	44848	44848	44848	1	42915	0	0	0	0	0	1.05	0
LMP1	V9GZ56	LSM4	40807	40807	40807	1	36396	0	0	0	0	0	1.12	0
LMP1	Q08211	DHX9	6395	6395	6395	1	10880	0	0	0	0	0	0.59	0
LMP1	G8JLB6	HNRNPH1	42433	42433	42433	1	34508	0	0	0	0	0	1.23	0
LMP1	Q13501	SQSTM1	18709	18709	18709	1	31213	0	0	0	0	0	0.6	0
LMP1	A0A024R6S1	DNAJA2	10017	10017	10017	1	24154	0	0	0	0	0	0.41	0
LMP1	P13010	XRCC5	20307	20307	20307	1	63230	0	0	0	0	0	0.32	0
LMP1	Q6P5S8	IGK@	63987	63987	63987	1	42397	0	0	0	0	0	1.51	0
LMP1	A0A024R2K4	LRRFIP2	62276	62276	62276	1	42885	0	0	0	0	0	1.45	0
LMP1	Q9Y512	SAMM50	56445	56445	56445	1	41978	0	0	0	0	0	1.34	0
LMP1	A2J1N9		22951	22951	22951	1	31450	0	0	0	0	0	0.73	0
LMP1	Q5VWZ2	LYPLAL1	58844	58844	58844	1	46930	0	0	0	0	0	1.25	0
LMP1	Q9UL86		41834	41834	41834	1	37862	0	0	0	0	0	1.1	0
LMP1	Q9H936	SLC25A22	60618	60618	60618	1	26234	0	0	0	0	0	2.31	0
LMP1	A0A024RAI1	ACTR3	7506	7506	7506	1	52545	0	0	0	0	0	0.14	0
LMP1	D9HTE9	SLC25A1	30537	30537	30537	1	61053	0	0	0	0	0	0.5	0
LMP1	A0A024RCJ8		42296	42296	42296	1	56178	0	0	0	0	0	0.75	0
LMP1	P01709	IGLV2-8	36119	36119	36119	1	20592	0	0	0	0	0	1.75	0
LMP1	A0A024R7T3	HNRPF	15207	15207	15207	1	52692	0	0	0	0	0	0.29	0
LMP1	B2R4D5		47923	47923	47923	1	61764	0	0	0	0	0	0.78	0
LMP1	E9PR30	FAU	10800	10800	10800	1	45153	0	0	0	0	0	0.24	0
LMP1	Q15293	RCN1	57422	57422	57422	1	25294	0	0	0	0	0	2.27	0
LMP1	P27694	RPA1	49683	49683	49683	1	61094	0	0	0	0	0	0.81	0
LMP1	P01704	IGLV2-14	19471	19471	19471	1	24288	0	0	0	0	0	0.8	0
LMP1	A0A140VJW5		22905	22905	22905	1	51496	0	0	0	0	0	0.44	0
LMP1	P37802	TAGLN2	45690	45690	45690	1	21091	0	0	0	0	0	2.17	0
LMP1	A0A075B6R9	IGKV2D-24	30358	30358	30358	1	46575	0	0	0	0	0	0.65	0
LMP1	E5KNY5	LRPPRC	16730	16730	16730	1	56584	0	0	0	0	0	0.3	0
LMP1	Q14444	CAPRIN1	12019	12019	12019	1	44855	0	0	0	0	0	0.27	0
LMP1	Q99623	PHB2	45166	45166	45166	1	20206	0	0	0	0	0	2.24	0
LMP1	B2R7F8		4633	4633	4633	1	54076	0	0	0	0	0	0.09	0
LMP1	E9KL35		58037	58037	58037	1	62725	0	0	0	0	0	0.93	0
LMP1	Q5EC54	HNRPK	31969	31969	31969	1	42942	0	0	0	0	0	0.74	0
LMP1	Q96K68		8570	8570	8570	1	21612	0	0	0	0	0	0.4	0
LMP1	Q6P089	IGH@	55107	55107	55107	1	65239	0	0	0	0	0	0.84	0
LMP1	P15153	RAC2	3233	3233	3233	1	57709	0	0	0	0	0	0.06	0
LMP1	Q13283	G3BP1	46226	46226	46226	1	54421	0	0	0	0	0	0.85	0
LMP1	A2NYU7		1990	1990	1990	1	2223	0	0	0	0	0	0.9	0
LMP1	P31689	DNAJA1	13363	13363	13363	1	61076	0	0	0	0	0	0.22	0
LMP1	A0A024RBB7	NAP1L1	3190	3190	3190	1	6681	0	0	0	0	0	0.48	0
LMP1	P28799	GRN	34250	34250	34250	1	48496	0	0	0	0	0	0.71	0
LMP1	Q5QPK2	DPM1	24977	24977	24977	1	23154	0	0	0	0	0	1.08	0
LMP1	A0A0X9TD88		63411	63411	63411	1	64003	0	0	0	0	0	0.99	0
LMP1	P36542	ATP5F1C	4779	4779	4779	1	10055	0	0	0	0	0	0.48	0
LMP1	B2R4R0	HIST1H4L	48102	48102	48102	1	20308	0	0	0	0	0	2.37	0
LMP1	P15814	IGLL1	55322	55322	55322	1	47354	0	0	0	0	0	1.17	0
LMP1	A0A024R8P8	RPL38	19862	19862	19862	1	11066	0	0	0	0	0	1.79	0
LMP1	P41091	EIF2S3	65032	65032	65032	1	45127	0	0	0	0	0	1.44	0
LMP1	A0A024RBA9	RAB21	40264	40264	40264	1	36378	0	0	0	0	0	1.11	0
LMP1	A8K5Y7		23635	23635	23635	1	13872	0	0	0	0	0	1.7	0
LMP1	V9HW34	HEL-213	22370	22370	22370	1	19865	0	0	0	0	0	1.13	0
LMP1	A0A0F7KYT8	FXR1	7194	7194	7194	1	60454	0	0	0	0	0	0.12	0
LMP1	P07814	EPRS	49729	49729	49729	1	56978	0	0	0	0	0	0.87	0
LMP1	A0A0C7DW92	HLA-DQA1	46997	46997	46997	1	21845	0	0	0	0	0	2.15	0
LMP1	P05198	EIF2S1	1790	1790	1790	1	24758	0	0	0	0	0	0.07	0
LMP1	A2MYE1		10484	10484	10484	1	30099	0	0	0	0	0	0.35	0
LMP1	Q96K37	SLC35E1	39301	39301	39301	1	61108	0	0	0	0	0	0.64	0
LMP1	A0A125U0V2		12901	12901	12901	1	56219	0	0	0	0	0	0.23	0
LMP1	F4ZW62		29608	29608	29608	1	21199	0	0	0	0	0	1.4	0
LMP1	B4DR61	SEC61A1	44552	44552	44552	1	38475	0	0	0	0	0	1.16	0
LMP1	P42224	STAT1	33023	33023	33023	1	61654	0	0	0	0	0	0.54	0
LMP1	A0A0G2JMH6	HLA-DRA	7260	7260	7260	1	33796	0	0	0	0	0	0.21	0
LMP1	A0A075B6R2	IGHV4-4	27494	27494	27494	1	35786	0	0	0	0	0	0.77	0
LMP1	Q59H77		14188	14188	14188	1	54617	0	0	0	0	0	0.26	0
LMP1	A0A024RDT4	LCP1	28325	28325	28325	1	42597	0	0	0	0	0	0.66	0
LMP1	Q562R1	ACTBL2	41454	41454	41454	1	30716	0	0	0	0	0	1.35	0
LMP1	Q9Y3Z3	SAMHD1	20277	20277	20277	1	25005	0	0	0	0	0	0.81	0
LMP1	A0A0S2Z3L2	ATP2A2	54306	54306	54306	1	29104	0	0	0	0	0	1.87	0
LMP1	B3KM74		26979	26979	26979	1	33706	0	0	0	0	0	0.8	0
LMP1	B2R9K8		45031	45031	45031	1	62955	0	0	0	0	0	0.72	0
LMP1	A0A024R0M6	TIMM50	42756	42756	42756	1	32976	0	0	0	0	0	1.3	0
LMP1	Q0ZCI2		48256	48256	48256	1	22037	0	0	0	0	0	2.19	0
LMP1	Q549C5	MST065	57696	57696	57696	1	62274	0	0	0	0	0	0.93	0
LMP1	P04406	GAPDH	58858	58858	58858	1	34584	0	0	0	0	0	1.7	0
LMP1	A0A075B7F0	IGHV3OR16-10	9930	9930	9930	1	30108	0	0	0	0	0	0.33	0
LMP1	A0A0U3GXA9	CXCR4	5759	5759	5759	1	62048	0	0	0	0	0	0.09	0
LMP1	P62136	PPP1CA	23364	23364	23364	1	13141	0	0	0	0	0	1.78	0
LMP1	P23528	CFL1	17555	17555	17555	1	39695	0	0	0	0	0	0.44	0
LMP1	P28908	TNFRSF8	12958	12958	12958	1	40154	0	0	0	0	0	0.32	0
LMP1	A0A024R5F7	DHCR7	43407	43407	43407	1	26813	0	0	0	0	0	1.62	0

LMP1	Q6N095	DKFZp686K03196	13063	13063	13063	1	43675	0	0	0	0	0	0.3	0
LMP1	Q969V3	NCLN	6329	6329	6329	1	64752	0	0	0	0	0	0.1	0
LMP1	P51571	SSR4	60363	60363	60363	1	58042	0	0	0	0	0	1.04	0
LMP1	B2R8R5		21148	21148	21148	1	37160	0	0	0	0	0	0.57	0
LMP1	A0A024RB87	RAP1B	51342	51342	51342	1	25261	0	0	0	0	0	2.03	0
LMP1	Q59EA2		4793	4793	4793	1	32394	0	0	0	0	0	0.15	0
LMP1	P12268	IMPDH2	19922	19922	19922	1	54913	0	0	0	0	0	0.36	0
LMP1	A2NYV1		55657	55657	55657	1	35140	0	0	0	0	0	1.58	0
LMP1	Q9BTV4	TMEM43	63757	63757	63757	1	51739	0	0	0	0	0	1.23	0
LMP1	O15143	ARPC1B	41541	41541	41541	1	42612	0	0	0	0	0	0.97	0
LMP1	Q59F66		4849	4849	4849	1	4391	0	0	0	0	0	1.1	0
LMP1	P02747	C1QC	58842	58842	58842	1	39180	0	0	0	0	0	1.5	0
LMP1	A8K401	PHB	28846	28846	28846	1	65478	0	0	0	0	0	0.44	0
LMP1	P62847	RPS24	58143	58143	58143	1	46210	0	0	0	0	0	1.26	0
LMP1	Q16531	DDB1	31908	31908	31908	1	63889	0	0	0	0	0	0.5	0
LMP1	A0A0C4DH24	IGKV6-21	39750	39750	39750	1	59018	0	0	0	0	0	0.67	0
LMP1	Q9NVH1	DNAJC11	24349	24349	24349	1	57603	0	0	0	0	0	0.42	0
LMP1	P04843	RPN1	12418	12418	12418	1	55302	0	0	0	0	0	0.22	0
LMP1	B2R4P2		24766	24766	24766	1	18387	0	0	0	0	0	1.35	0
LMP1	Q8N8A2	ANKRD44	21737	21737	21737	1	57206	0	0	0	0	0	0.38	0
LMP1	S6B2B6		2347	2347	2347	1	4720	0	0	0	0	0	0.5	0
LMP1	Q14257	RCN2	2664	2664	2664	1	10176	0	0	0	0	0	0.26	0
LMP1	A0A0A0MTC1	RNF213	35724	35724	35724	1	18850	0	0	0	0	0	1.9	0
LMP1	K4ENJ5		39380	39380	39380	1	58273	0	0	0	0	0	0.68	0
LMP1	P14317	HCLS1	45011	45011	45011	1	50426	0	0	0	0	0	0.89	0
LMP1	A0A120HG44		48305	48305	48305	1	25107	0	0	0	0	0	1.92	0
LMP1	P36957	DLST	1753	1753	1753	1	46225	0	0	0	0	0	0.04	0
LMP1	Q562Z4	ACT	17632	17632	17632	1	7524	0	0	0	0	0	2.34	0
LMP1	Q6IAX1	FDFT1	13511	13511	13511	1	39394	0	0	0	0	0	0.34	0
LMP1	O96008	TOMM40	11102	11102	11102	1	40878	0	0	0	0	0	0.27	0
LMP1	D3DUZ3	IFI16	23573	23573	23573	1	39991	0	0	0	0	0	0.59	0
LMP1	A0A087WU53	MAGT1	2361	2361	2361	1	61884	0	0	0	0	0	0.04	0
LMP1	Q96HS1	PGAM5	21813	21813	21813	1	26424	0	0	0	0	0	0.83	0
LMP1	H7BY58	PCMT1	54612	54612	54612	1	32985	0	0	0	0	0	1.66	0
LMP1	O15511	ARPC5	7405	7405	7405	1	22607	0	0	0	0	0	0.33	0
LMP1	B2RE46		23850	23850	23850	1	48306	0	0	0	0	0	0.49	0
LMP1	Q5T1Z8	PUM1	2579	2579	2579	1	54409	0	0	0	0	0	0.05	0
LMP1	P02746	C1QB	20270	20270	20270	1	26919	0	0	0	0	0	0.75	0
LMP1	A0A0A0MT36	IGKV6D-21	14782	14782	14782	1	46031	0	0	0	0	0	0.32	0
LMP1	G1FM90		28740	28740	28740	1	59423	0	0	0	0	0	0.48	0
LMP1	P22695	UQCRC2	19787	19787	19787	1	55547	0	0	0	0	0	0.36	0
LMP1	Q9UBB4	ATXN10	58527	58527	58527	1	61500	0	0	0	0	0	0.95	0
LMP1	C9IZQ1	SSR1	39071	39071	39071	1	35311	0	0	0	0	0	1.11	0
LMP1	J3KQE5	RAN	57630	57630	57630	1	53890	0	0	0	0	0	1.07	0
LMP1	A2J1N7		64219	64219	64219	1	38458	0	0	0	0	0	1.67	0
LMP1	Q53SS8	PCBP1	56953	56953	56953	1	26957	0	0	0	0	0	2.11	0
LMP1	J3KTL2	SRSF1	42980	42980	42980	1	56536	0	0	0	0	0	0.76	0
LMP1	O94905	ERLIN2	16201	16201	16201	1	19382	0	0	0	0	0	0.84	0
LMP1	Q8NE71	ABCF1	53730	53730	53730	1	43157	0	0	0	0	0	1.24	0
LMP1	Q9Y6C9	MTCH2	3185	3185	3185	1	49849	0	0	0	0	0	0.06	0
LMP1	Q15366	PCBP2	5282	5282	5282	1	39183	0	0	0	0	0	0.13	0
LMP1	P55084	HADHB	39451	39451	39451	1	44694	0	0	0	0	0	0.88	0
LMP1	D9ZGG2	VTN	41672	41672	41672	1	21911	0	0	0	0	0	1.9	0
LMP1	P55060	CSE1L	64887	64887	64887	1	29794	0	0	0	0	0	2.18	0
LMP1	Q15046	KARS	61666	61666	61666	1	46009	0	0	0	0	0	1.34	0
LMP1	Q9Y5A9	YTHDF2	17154	17154	17154	1	42272	0	0	0	0	0	0.41	0
LMP1	B3KM36		43693	43693	43693	1	28343	0	0	0	0	0	1.54	0
LMP1	A2NYU8		11309	11309	11309	1	20064	0	0	0	0	0	0.56	0
LMP1	P53621	COPA	53308	53308	53308	1	27899	0	0	0	0	0	1.91	0
LMP1	A0A0S2Z4Z9	NONO	55692	55692	55692	1	59562	0	0	0	0	0	0.94	0
LMP1	B7Z268	SSBP1	51525	51525	51525	1	23753	0	0	0	0	0	2.17	0
LMP1	B9EK46	CGN	15148	15148	15148	1	59588	0	0	0	0	0	0.25	0
LMP1	D3YPT7	COX2	36825	36825	36825	1	61957	0	0	0	0	0	0.59	0
LMP1	P01766	IGHV3-13	41727	41727	41727	1	28435	0	0	0	0	0	1.47	0
LMP1	P60468	SEC61B	32464	32464	32464	1	35328	0	0	0	0	0	0.92	0
LMP1	E7EQZ4	SMN1	19169	19169	19169	1	49590	0	0	0	0	0	0.39	0
LMP1	A2NXP8		4505	4505	4505	1	27822	0	0	0	0	0	0.16	0
LMP1	P46087	NOP2	19650	19650	19650	1	28521	0	0	0	0	0	0.69	0
LMP1	A8K492		65237	65237	65237	1	44969	0	0	0	0	0	1.45	0
LMP1	A0A140T9T7	TAP1	41214	41214	41214	1	42862	0	0	0	0	0	0.96	0
LMP1	C9J6P4	ZC3HAV1	56839	56839	56839	1	42697	0	0	0	0	0	1.33	0
LMP1	Q13257	MAD2L1	61844	61844	61844	1	24858	0	0	0	0	0	2.49	0
LMP1	Q96P70	IPO9	58498	58498	58498	1	56876	0	0	0	0	0	1.03	0
LMP1	P52701	MSH6	43316	43316	43316	1	57128	0	0	0	0	0	0.76	0
LMP1	C0JYY2	APOB	18768	18768	18768	1	29864	0	0	0	0	0	0.63	0
LMP1	P62070	RRAS2	6070	6070	6070	1	23987	0	0	0	0	0	0.25	0
LMP1	B2R6F3	SFRS3	37515	37515	37515	1	41980	0	0	0	0	0	0.89	0
LMP1	O15260	SURF4	25865	25865	25865	1	58264	0	0	0	0	0	0.44	0
LMP1	B3KY94	CDIPT	36565	36565	36565	1	23882	0	0	0	0	0	1.53	0
LMP1	B2RB52		5283	5283	5283	1	46030	0	0	0	0	0	0.11	0
LMP1	P83111	LACTB	57866	57866	57866	1	53407	0	0	0	0	0	1.08	0
LMP1	P62316	SNRPD2	51919	51919	51919	1	22659	0	0	0	0	0	2.29	0
LMP1	Q8NF37	LPCAT1	46754	46754	46754	1	24388	0	0	0	0	0	1.92	0
LMP1	A0A024R5Z9	PKM2	23015	23015	23015	1	13941	0	0	0	0	0	1.65	0
LMP1	Q2M1J6	OXA1L	31393	31393	31393	1	27652	0	0	0	0	0	1.14	0
LMP1	P04899	GNAI2	15645	15645	15645	1	29799	0	0	0	0	0	0.53	0
LMP1	P40763	STAT3	24915	24915	24915	1	34748	0	0	0	0	0	0.72	0
LMP1	O75569	PRKRA	1497	1497	1497	1	40843	0	0	0	0	0	0.04	0
LMP1	A8K7F6		27901	27901	27901	1	36734	0	0	0	0	0	0.76	0
LMP1	B3KQ33		8388	8388	8388	1	35623	0	0	0	0	0	0.24	0
LMP1	E5KT65		40020	40020	40020	1	17592	0	0	0	0	0	2.27	0
LMP1	A8KA19		35996	35996	35996	1	58573	0	0	0	0	0	0.61	0
LMP1	Q0ZCH6		12063	12063	12063	1	51908	0	0	0	0	0	0.23	0
LMP1	P06733	ENO1	25972	25972	25972	1	37981	0	0	0	0	0	0.68	0
LMP1	Q9Y295	DRG1	58077	58077	58077	1	36480	0	0	0	0	0	1.59	0
LMP1	Q8IVM0	CCDC50	34261	34261	34261	1	56206	0	0	0	0	0	0.61	0
LMP1	P42166	TMPO	50915	50915	50915	1	25506	0	0	0	0	0	2	0
LMP1	P14625	HSP90B1	17214	17214	17214	1	24703	0	0	0	0	0	0.7	0
LMP1	O00159	MYO1C	21940	21940	21940	1	25326	0	0	0	0	0	0.87	0
LMP1	A8K132		61966	61966	61966	1	38073	0	0	0	0	0	1.63	0

LMP1	P10909	CLU	31378	31378	31378	1	36362	0	0	0	0	0	0.86	0
LMP1	Q4KMQ1	TPRN	4412	4412	4412	1	41590	0	0	0	0	0	0.11	0
LMP1	Q96T76	MMS19	6538	6538	6538	1	38734	0	0	0	0	0	0.17	0
LMP1	S6C4Q9		15854	15854	15854	1	58941	0	0	0	0	0	0.27	0
LMP1	A0A024R2T6	DHX30	3869	3869	3869	1	5329	0	0	0	0	0	0.73	0
LMP1	P26641	EEF1G	2468	2468	2468	1	38704	0	0	0	0	0	0.06	0
LMP1	O95831	AIFM1	61225	61225	61225	1	47616	0	0	0	0	0	1.29	0
LMP1	A0A068LKR7		44894	44894	44894	1	57972	0	0	0	0	0	0.77	0
LMP1	O43324	EEF1E1	13459	13459	13459	1	51212	0	0	0	0	0	0.26	0
LMP1	O75037	KIF21B	909	909	909	1	34207	0	0	0	0	0	0.03	0
LMP1	Q8NF20	FLJ00382	44505	44505	44505	1	51613	0	0	0	0	0	0.86	0
LMP1	A0A087WTJ2	GIMAP1-GIMAP5	43474	43474	43474	1	36858	0	0	0	0	0	1.18	0
LMP1	A8K6Q8		35951	35951	35951	1	30276	0	0	0	0	0	1.19	0
LMP1	A0A024R912	UCK2	70	70	70	1	10752	0	0	0	0	0	0.01	0
LMP1	Q6PKG0	LARP1	63842	63842	63842	1	57866	0	0	0	0	0	1.1	0
LMP1	Q6NXE6	ARMC6	49264	49264	49264	1	24370	0	0	0	0	0	2.02	0
LMP1	P61026	RAB10	18263	18263	18263	1	24154	0	0	0	0	0	0.76	0
LMP1	B4DJ38		55732	55732	55732	1	63516	0	0	0	0	0	0.88	0
LMP1	P78371	CCT2	30767	30767	30767	1	55147	0	0	0	0	0	0.56	0
LMP1	P16402	HIST1H1D	22751	22751	22751	1	49812	0	0	0	0	0	0.46	0
LMP1	A0A024RDE5	G3BP2	52487	52487	52487	1	57277	0	0	0	0	0	0.92	0
LMP1	V9GYM8	ARHGEF2	35740	35740	35740	1	62091	0	0	0	0	0	0.58	0
LMP1	A0A024R9D7	DECR1	26018	26018	26018	1	54433	0	0	0	0	0	0.48	0
LMP1	O75427	LRCH4	13642	13642	13642	1	20489	0	0	0	0	0	0.67	0
LMP1	O15269	SPTLC1	35245	35245	35245	1	15556	0	0	0	0	0	2.27	0
LMP1	Q9BRK5	SDF4	384	384	384	1	55117	0	0	0	0	0	0.01	0
LMP1	P04350	TUBB4A	7548	7548	7548	1	33635	0	0	0	0	0	0.22	0
LMP1	Q59GW5		47442	47442	47442	1	56635	0	0	0	0	0	0.84	0
LMP1	Q9BYM8	RBCK1	41207	41207	41207	1	47822	0	0	0	0	0	0.86	0
LMP1	D3DTH7	MYO1C	30180	30180	30180	1	23192	0	0	0	0	0	1.3	0
LMP1	P26038	MSN	5908	5908	5908	1	23160	0	0	0	0	0	0.26	0
LMP1	P16989	YBX3	23222	23222	23222	1	23527	0	0	0	0	0	0.99	0
LMP1	Q00653	NFKB2	14782	14782	14782	1	19553	0	0	0	0	0	0.76	0
LMP1	A8K690		34716	34716	34716	1	26553	0	0	0	0	0	1.31	0
LMP1	Q8TA92		26020	26020	26020	1	23189	0	0	0	0	0	1.12	0
LMP1	N1NV67	HLA-A	4929	4929	4929	1	60476	0	0	0	0	0	0.08	0
LMP1	O60488	ACSL4	38451	38451	38451	1	34774	0	0	0	0	0	1.11	0
LMP1	F8W1S1	KRT74	30496	30496	30496	1	56133	0	0	0	0	0	0.54	0
LMP1	Q92556	ELMO1	53220	53220	53220	1	25382	0	0	0	0	0	2.1	0
LMP1	Q9Y5M8	SRPRB	50302	50302	50302	1	52434	0	0	0	0	0	0.96	0
LMP1	Q7Z2U7		18299	18299	18299	1	29141	0	0	0	0	0	0.63	0
LMP1	Q53HV2		59210	59210	59210	1	41216	0	0	0	0	0	1.44	0
LMP1	A0A024R0C0	CEPT1	41059	41059	41059	1	54623	0	0	0	0	0	0.75	0
LMP1	P30049	ATP5F1D	18368	18368	18368	1	26678	0	0	0	0	0	0.69	0
LMP1	P46063	RECQL	10194	10194	10194	1	7701	0	0	0	0	0	1.32	0
LMP1	P30048	PRDX3	37063	37063	37063	1	19558	0	0	0	0	0	1.9	0
LMP1	P54136	RARS	38433	38433	38433	1	34469	0	0	0	0	0	1.12	0
LMP1	B5BU01	EIF2S2	34982	34982	34982	1	60590	0	0	0	0	0	0.58	0
LMP1	P51991	HNRNPA3	30311	30311	30311	1	27883	0	0	0	0	0	1.09	0
LMP1	P48047	ATP5PO	31374	31374	31374	1	18139	0	0	0	0	0	1.73	0
LMP1	A0A087WZN1	IDH3B	32553	32553	32553	1	55332	0	0	0	0	0	0.59	0
LMP1	P62258	YWHAE	8660	8660	8660	1	11222	0	0	0	0	0	0.77	0
LMP1	S6B294		37538	37538	37538	1	61331	0	0	0	0	0	0.61	0
LMP1	Q5H9N4	DKFZp686L20222	12362	12362	12362	1	63954	0	0	0	0	0	0.19	0
LMP1	B5BU24	YWHAB	47478	47478	47478	1	42350	0	0	0	0	0	1.12	0
LMP1	Q13344		10521	10521	10521	1	7172	0	0	0	0	0	1.47	0
LMP1	A0A109NGN6		40054	40054	40054	1	26186	0	0	0	0	0	1.53	0
LMP1	A0A024R324	RHOA	39825	39825	39825	1	63280	0	0	0	0	0	0.63	0
LMP1	E9KL48	GLUD1	46402	46402	46402	1	52862	0	0	0	0	0	0.88	0
LMP1	E5KSX8		54677	54677	54677	1	28876	0	0	0	0	0	1.89	0
LMP1	A8K5K0		32155	32155	32155	1	14958	0	0	0	0	0	2.15	0
LMP1	A0A0S2Z5U6	PYCR2	50193	50193	50193	1	55454	0	0	0	0	0	0.91	0
LMP1	A0A024R7F9	GCDH	11015	11015	11015	1	23460	0	0	0	0	0	0.47	0
LMP1	Q9H444	CHMP4B	51918	51918	51918	1	53329	0	0	0	0	0	0.97	0
LMP1	O95466	FMNL1	80	80	80	1	11704	0	0	0	0	0	0.01	0
LMP1	P27824	CANX	54652	54652	54652	1	33309	0	0	0	0	0	1.64	0
LMP1	V9HW37	HEL-S-69	48158	48158	48158	1	55134	0	0	0	0	0	0.87	0
LMP1	A0A0S2Z507	BTRC	3067	3067	3067	1	18648	0	0	0	0	0	0.16	0
LMP1	Q7L8L6	FASTKD5	41254	41254	41254	1	18932	0	0	0	0	0	2.18	0
LMP1	Q5JPC1	DKFZp667O1614	60978	60978	60978	1	30455	0	0	0	0	0	2	0
LMP1	V9HW87	HEL-S-299	28573	28573	28573	1	43280	0	0	0	0	0	0.66	0
LMP1	P49327	FASN	3104	3104	3104	1	57578	0	0	0	0	0	0.05	0
LMP1	V9HWA9	HEL-S-62p	37057	37057	37057	1	52511	0	0	0	0	0	0.71	0
LMP1	P00338	LDHA	29734	29734	29734	1	52942	0	0	0	0	0	0.56	0
LMP1	Q15717	ELAVL1	63888	63888	63888	1	31929	0	0	0	0	0	2	0
LMP1	Q65ZC9	scFv	23652	23652	23652	1	32183	0	0	0	0	0	0.73	0
LMP1	A0A024R1K7	YWHAH	61960	61960	61960	1	50905	0	0	0	0	0	1.22	0
LMP1	Q9NYL4	FKBP11	6608	6608	6608	1	5755	0	0	0	0	0	1.15	0
LMP1	Q86VV6	MMP24	6899	6899	6899	1	20879	0	0	0	0	0	0.33	0
LMP1	O95373	IPO7	22527	22527	22527	1	10532	0	0	0	0	0	2.14	0
LMP1	Q9NTJ3	SMC4	237	237	237	1	36042	0	0	0	0	0	0.01	0
LMP1	A0A0B4J2D9	IGKV1D-13	17445	17445	17445	1	46780	0	0	0	0	0	0.37	0
LMP1	Q53R41	FASTKD1	12407	12407	12407	1	39178	0	0	0	0	0	0.32	0
LMP1	Q6IQ30	PABPC4	4450	4450	4450	1	24739	0	0	0	0	0	0.18	0
LMP1	Q8NDX1	PSD4	40686	40686	40686	1	19220	0	0	0	0	0	2.12	0
LMP1	B5BUB1	RUVBL1	55080	55080	55080	1	30032	0	0	0	0	0	1.83	0
LMP1	O14828	SCAMP3	64243	64243	64243	1	60803	0	0	0	0	0	1.06	0
LMP1	A0A0S2Z428	KRT6A	6982	6982	6982	1	23199	0	0	0	0	0	0.3	0
LMP1	A0N7I9	F5-20	33262	33262	33262	1	53405	0	0	0	0	0	0.62	0
LMP1	B3KSH1	EIF3F	6837	6837	6837	1	52229	0	0	0	0	0	0.13	0
LMP1	H0Y8C2	RPL22L1	10742	10742	10742	1	7071	0	0	0	0	0	1.52	0
LMP1	Q0ZCI6		56373	56373	56373	1	49155	0	0	0	0	0	1.15	0
LMP1	A8K9A4		17851	17851	17851	1	60630	0	0	0	0	0	0.29	0
LMP1	Q9Y4B6	DCAF1	19322	19322	19322	1	64682	0	0	0	0	0	0.3	0
LMP1	A8K3C3		8832	8832	8832	1	8144	0	0	0	0	0	1.08	0
LMP1	A0A087WWE2		36676	36676	36676	1	27918	0	0	0	0	0	1.31	0
LMP1	A8K7D9		11604	11604	11604	1	64295	0	0	0	0	0	0.18	0
LMP1	C9J7E5	TNPO3	30772	30772	30772	1	36206	0	0	0	0	0	0.85	0
LMP1	B3KWN0		32360	32360	32360	1	46847	0	0	0	0	0	0.69	0
LMP1	E7EW20	MYO6	13675	13675	13675	1	23544	0	0	0	0	0	0.58	0

LMP1	A0A0U5JA32	TNLG8A	22194	22194	22194	1	59436	0	0	0	0	0	0.37	0
LMP1	Q02338	BDH1	45500	45500	45500	1	40672	0	0	0	0	0	1.12	0
LMP1	A0A0A0MQS1	PYCR3	3583	3583	3583	1	5718	0	0	0	0	0	0.63	0
LMP1	A0A0A0MT69	IGKJ4	1809	1809	1809	1	32078	0	0	0	0	0	0.06	0
LMP1	Q5UIP0	RIF1	17224	17224	17224	1	31373	0	0	0	0	0	0.55	0
LMP1	A8K725		14541	14541	14541	1	17994	0	0	0	0	0	0.81	0
LMP1	P17987	TCP1	62285	62285	62285	1	30196	0	0	0	0	0	2.06	0
LMP1	A0A024R3R5	LBR	13050	13050	13050	1	65517	0	0	0	0	0	0.2	0
LMP1	B5ME19	EIF3CL	10053	10053	10053	1	30768	0	0	0	0	0	0.33	0
LMP1	H0Y8C6	IPO5	3986	3986	3986	1	9951	0	0	0	0	0	0.4	0
LMP1	Q09028	RBBP4	48836	48836	48836	1	49149	0	0	0	0	0	0.99	0
LMP1	B2RDG1		19486	19486	19486	1	24283	0	0	0	0	0	0.8	0
LMP1	Q6NS36	FTH1	28923	28923	28923	1	26450	0	0	0	0	0	1.09	0
LMP1	Q59EG8		4610	4610	4610	1	2998	0	0	0	0	0	1.54	0
LMP1	F5H423		30047	30047	30047	1	62273	0	0	0	0	0	0.48	0
LMP1	E9KL44		9042	9042	9042	1	38721	0	0	0	0	0	0.23	0
LMP1	Q6ZUJ8	PIK3AP1	47963	47963	47963	1	53729	0	0	0	0	0	0.89	0
LMP1	P08779	KRT16	38821	38821	38821	1	44622	0	0	0	0	0	0.87	0
LMP1	Q15182	SNRPB	13251	13251	13251	1	42525	0	0	0	0	0	0.31	0
LMP1	O00743	PPP6C	20444	20444	20444	1	57767	0	0	0	0	0	0.35	0
LMP1	A0A024QZT2	EXOC2	3090	3090	3090	1	47217	0	0	0	0	0	0.07	0
LMP1	Q15370	ELOB	45962	45962	45962	1	24480	0	0	0	0	0	1.88	0
LMP1	A2NW98		33923	33923	33923	1	60798	0	0	0	0	0	0.56	0
LMP1	V9HW88	HEL-S-99n	19164	19164	19164	1	37743	0	0	0	0	0	0.51	0
LMP1	P61513	RPL37A	15294	15294	15294	1	13946	0	0	0	0	0	1.1	0
LMP1	Q8NB90	SPATA5	32007	32007	32007	1	37623	0	0	0	0	0	0.85	0
LMP1	Q13724	MOGS	31683	31683	31683	1	59566	0	0	0	0	0	0.53	0
LMP1	A2NKM7		61889	61889	61889	1	52281	0	0	0	0	0	1.18	0
LMP1	Q92900	UPF1	60658	60658	60658	1	61769	0	0	0	0	0	0.98	0
LMP1	Q8N3C0	ASCC3	25824	25824	25824	1	18932	0	0	0	0	0	1.36	0
LMP1	B2R806	EIF3E	43248	43248	43248	1	43456	0	0	0	0	0	1	0
LMP1	E9PF32	DENND3	65150	65150	65150	1	36338	0	0	0	0	0	1.79	0
LMP1	A0A1B2JLU7	PYCR1	58537	58537	58537	1	42662	0	0	0	0	0	1.37	0
LMP1	P53618	COPB1	37385	37385	37385	1	44209	0	0	0	0	0	0.85	0
LMP1	Q32MZ4	LRRFIP1	10846	10846	10846	1	60358	0	0	0	0	0	0.18	0
LMP1	A0A024RDS1	HSPH1	1285	1285	1285	1	44998	0	0	0	0	0	0.03	0
LMP1	P30101	PDIA3	5748	5748	5748	1	31055	0	0	0	0	0	0.19	0
LMP1	A0A024R6W2	NUDT21	64564	64564	64564	1	29269	0	0	0	0	0	2.21	0
LMP1	H3BS72	HACD3	58755	58755	58755	1	54425	0	0	0	0	0	1.08	0
LMP1	A0A0S2Z3H3	SLC25A4	2550	2550	2550	1	42072	0	0	0	0	0	0.06	0
LMP1	Q9NWB6	ARGLU1	50200	50200	50200	1	58956	0	0	0	0	0	0.85	0
LMP1	E6Y8C6	PRF1	3985	3985	3985	1	36193	0	0	0	0	0	0.11	0
LMP1	Q9H061	TMEM126A	43569	43569	43569	1	42330	0	0	0	0	0	1.03	0
LMP1	B4E1T5		49171	49171	49171	1	38411	0	0	0	0	0	1.28	0
LMP1	A0A024R152		29875	29875	29875	1	30134	0	0	0	0	0	0.99	0
LMP1	O00483	NDUFA4	28439	28439	28439	1	31755	0	0	0	0	0	0.9	0
LMP1	A0A024R9Z8	AHR	32806	32806	32806	1	25791	0	0	0	0	0	1.27	0
LMP1	Q6NX51	EXOC4	50254	50254	50254	1	38077	0	0	0	0	0	1.32	0
LMP1	A0A109PW74		48633	48633	48633	1	63966	0	0	0	0	0	0.76	0
LMP1	A0A024R0E2	CSDE1	41089	41089	41089	1	28043	0	0	0	0	0	1.47	0
LMP1	B0YIW6	ARCN1	28500	28500	28500	1	25431	0	0	0	0	0	1.12	0
LMP1	Q96SB0		11635	11635	11635	1	7572	0	0	0	0	0	1.54	0
LMP1	M0R2B7	POLD1	11552	11552	11552	1	38651	0	0	0	0	0	0.3	0
LMP1	A8K032		53850	53850	53850	1	60023	0	0	0	0	0	0.9	0
LMP1	Q96FS4	SIPA1	23281	23281	23281	1	22016	0	0	0	0	0	1.06	0
LMP1	B2R7C2		4411	4411	4411	1	6547	0	0	0	0	0	0.67	0
LMP1	A8K3A8		57165	57165	57165	1	58462	0	0	0	0	0	0.98	0
LMP1	E7ESC6	XPO7	26184	26184	26184	1	16814	0	0	0	0	0	1.56	0
LMP1	P05413	FABP3	55403	55403	55403	1	39846	0	0	0	0	0	1.39	0
LMP1	P12004	PCNA	3941	3941	3941	1	32707	0	0	0	0	0	0.12	0
LMP1	Q9UHD2	TBK1	12607	12607	12607	1	7811	0	0	0	0	0	1.61	0
LMP1	P27348	YWHAQ	41547	41547	41547	1	40481	0	0	0	0	0	1.03	0
LMP1	Q86UP2	KTN1	60078	60078	60078	1	33186	0	0	0	0	0	1.81	0
LMP1	Q8TCJ2	STT3B	45759	45759	45759	1	60016	0	0	0	0	0	0.76	0
LMP1	A0A087X211	CIP2A	21559	21559	21559	1	33785	0	0	0	0	0	0.64	0
LMP1	O60725	ICMT	23171	23171	23171	1	63545	0	0	0	0	0	0.36	0
LMP1	Q4LE58	EIF4G1 variant protein	6711	6711	6711	1	10421	0	0	0	0	0	0.64	0
LMP1	S6C4S0		38469	38469	38469	1	62551	0	0	0	0	0	0.62	0
LMP1	B9EG90	TOP1	9320	9320	9320	1	44866	0	0	0	0	0	0.21	0
LMP1	P02763	ORM1	35403	35403	35403	1	36962	0	0	0	0	0	0.96	0
LMP1	A8K6I5		64480	64480	64480	1	32462	0	0	0	0	0	1.99	0
LMP1	Q01650	SLC7A5	7479	7479	7479	1	53668	0	0	0	0	0	0.14	0
LMP1	O00165	HAX1	51617	51617	51617	1	40697	0	0	0	0	0	1.27	0
LMP1	Q59FD4		49986	49986	49986	1	42189	0	0	0	0	0	1.18	0
LMP1	O75592	MYCBP2	37181	37181	37181	1	35655	0	0	0	0	0	1.04	0
LMP1	Q96TA2	YME1L1	13446	13446	13446	1	21579	0	0	0	0	0	0.62	0
LMP1	A0A068LN13		28525	28525	28525	1	35699	0	0	0	0	0	0.8	0
LMP1	A0A024RD93	PAICS	39616	39616	39616	1	57164	0	0	0	0	0	0.69	0
LMP1	Q8TBD0		40040	40040	40040	1	44805	0	0	0	0	0	0.89	0
LMP1	E5KBQ3	TRAF2	60865	60865	60865	1	51495	0	0	0	0	0	1.18	0
LMP1	J3KPF3	SLC3A2	44790	44790	44790	1	25620	0	0	0	0	0	1.75	0
LMP1	Q9UPN7	PPP6R1	21312	21312	21312	1	56243	0	0	0	0	0	0.38	0
LMP1	Q96QR8	PURB	6374	6374	6374	1	24402	0	0	0	0	0	0.26	0
LMP1	P08574	CYC1	1035	1035	1035	1	16242	0	0	0	0	0	0.06	0
LMP1	B3KPC7		30264	30264	30264	1	47772	0	0	0	0	0	0.63	0
LMP1	O75947	ATP5PD	23302	23302	23302	1	38647	0	0	0	0	0	0.6	0
LMP1	P49721	PSMB2	48903	48903	48903	1	42010	0	0	0	0	0	1.16	0
LMP1	B2R5M8		56349	56349	56349	1	49172	0	0	0	0	0	1.15	0
LMP1	A2JA17		23689	23689	23689	1	18141	0	0	0	0	0	1.31	0
LMP1	A0A0X9V9C4		44126	44126	44126	1	22393	0	0	0	0	0	1.97	0
LMP1	Q59EK7		20309	20309	20309	1	56773	0	0	0	0	0	0.36	0
LMP1	E5RJR5	SKP1	64053	64053	64053	1	44805	0	0	0	0	0	1.43	0
LMP1	Q14498	RBM39	16513	16513	16513	1	43187	0	0	0	0	0	0.38	0
LMP1	Q9UJZ1	STOML2	41046	41046	41046	1	24855	0	0	0	0	0	1.65	0
LMP1	Q14157	UBAP2L	31812	31812	31812	1	63902	0	0	0	0	0	0.5	0
LMP1	Q9NTJ5	SACM1L	57913	57913	57913	1	48639	0	0	0	0	0	1.19	0
LMP1	A0A024R6I7	SERPINA1	13007	13007	13007	1	53940	0	0	0	0	0	0.24	0
LMP1	Q549M8	C14orf166	59369	59369	59369	1	26551	0	0	0	0	0	2.24	0
LMP1	A0A125QYY7		38411	38411	38411	1	41361	0	0	0	0	0	0.93	0
LMP1	Q15393	SF3B3	2133	2133	2133	1	52747	0	0	0	0	0	0.04	0

LMP1	Q5U5J2	CSNK2A1	6873	6873	6873	1	44027	0	0	0	0	0	0.16	0
LMP1	A4D105	RPA3	34141	34141	34141	1	63434	0	0	0	0	0	0.54	0
LMP1	B2R665		62315	62315	62315	1	46207	0	0	0	0	0	1.35	0
LMP1	B4DL07		45164	45164	45164	1	21481	0	0	0	0	0	2.1	0
LMP1	P11388	TOP2A	22210	22210	22210	1	50656	0	0	0	0	0	0.44	0
LMP1	Q9H6D7	HAUS4	60543	60543	60543	1	39285	0	0	0	0	0	1.54	0
LMP1	A6QKW0	SHINC3	62946	62946	62946	1	30385	0	0	0	0	0	2.07	0
LMP1	A0A0B4J1V9	HELLS	59979	59979	59979	1	55098	0	0	0	0	0	1.09	0
LMP1	Q969X5	ERGIC1	16142	16142	16142	1	18644	0	0	0	0	0	0.87	0
LMP1	A0A024R7C0	KEAP1	19425	19425	19425	1	32585	0	0	0	0	0	0.6	0
LMP1	A0A109PT03		39939	39939	39939	1	42780	0	0	0	0	0	0.93	0
LMP1	P23381	WARS	31600	31600	31600	1	48253	0	0	0	0	0	0.65	0
LMP1	O95758	PTBP3	23449	23449	23449	1	23316	0	0	0	0	0	1.01	0
LMP1	P46977	STT3A	6925	6925	6925	1	56021	0	0	0	0	0	0.12	0
LMP1	Q9NU22	MDN1	26756	26756	26756	1	34357	0	0	0	0	0	0.78	0
LMP1	A8K4M4		8608	8608	8608	1	45489	0	0	0	0	0	0.19	0
LMP1	Q96AG4	LRRC59	11743	11743	11743	1	59073	0	0	0	0	0	0.2	0
LMP1	O60711	LPXN	52772	52772	52772	1	55933	0	0	0	0	0	0.94	0
LMP1	Q9HBL7	PLGRKT	27285	27285	27285	1	47443	0	0	0	0	0	0.58	0
LMP1	A0A1B4Z394	TUBG2	52736	52736	52736	1	48763	0	0	0	0	0	1.08	0
LMP1	O94927	HAUS5	44520	44520	44520	1	64205	0	0	0	0	0	0.69	0
LMP1	Q30030	HLA-DPB1	49268	49268	49268	1	46142	0	0	0	0	0	1.07	0
LMP1	O75390	CS	61987	61987	61987	1	30844	0	0	0	0	0	2.01	0
LMP1	P62879	GNB2	5584	5584	5584	1	58540	0	0	0	0	0	0.1	0
LMP1	Q9UL92		9923	9923	9923	1	55378	0	0	0	0	0	0.18	0
LMP1	B4E0Z6		18679	18679	18679	1	13714	0	0	0	0	0	1.36	0
LMP1	Q9BTY7	HGH1	64090	64090	64090	1	57575	0	0	0	0	0	1.11	0
LMP1	O14545	TRAFD1	64925	64925	64925	1	52220	0	0	0	0	0	1.24	0
LMP1	P78328		55075	55075	55075	1	38091	0	0	0	0	0	1.45	0
LMP1	A2N2G5	VH87-2	46547	46547	46547	1	27988	0	0	0	0	0	1.66	0
LMP1	B4E2S3		35184	35184	35184	1	28055	0	0	0	0	0	1.25	0
LMP1	P27105	STOM	65069	65069	65069	1	48388	0	0	0	0	0	1.34	0
LMP1	P35606	COPB2	35293	35293	35293	1	28254	0	0	0	0	0	1.25	0
LMP1	P14866	HNRNPL	31122	31122	31122	1	29951	0	0	0	0	0	1.04	0
LMP1	A0A024QZE6	C16orf58	27374	27374	27374	1	37156	0	0	0	0	0	0.74	0
LMP1	Q9P0L0	VAPA	59476	59476	59476	1	37745	0	0	0	0	0	1.58	0
LMP1	A2J423		56278	56278	56278	1	47567	0	0	0	0	0	1.18	0
LMP1	A5A3E0	POTEF	39730	39730	39730	1	56963	0	0	0	0	0	0.7	0
LMP1	Q86YZ3	HRNR	31190	31190	31190	1	12473	0	0	0	0	0	2.5	0
LMP1	Q92621	NUP205	2839	2839	2839	1	7930	0	0	0	0	0	0.36	0
LMP1	Q5HYM3	DKFZp686C0249	25335	25335	25335	1	45940	0	0	0	0	0	0.55	0
LMP1	Q59ES3		23069	23069	23069	1	12510	0	0	0	0	0	1.84	0
LMP1	A0A0A0MT89	IGKJ1	43388	43388	43388	1	24132	0	0	0	0	0	1.8	0
LMP1	Q86UX7	FERMT3	24894	24894	24894	1	11003	0	0	0	0	0	2.26	0
LMP1	Q9GZR7	DDX24	15226	15226	15226	1	6154	0	0	0	0	0	2.47	0
LMP1	A8K430		38868	38868	38868	1	48839	0	0	0	0	0	0.8	0
LMP1	P57740	NUP107	22096	22096	22096	1	43934	0	0	0	0	0	0.5	0
LMP1	Q59G75		11116	11116	11116	1	40117	0	0	0	0	0	0.28	0
LMP1	Q92769	HDAC2	29215	29215	29215	1	26419	0	0	0	0	0	1.11	0
LMP1	A0A0U4C4L3	HLA-DQA1	52613	52613	52613	1	50196	0	0	0	0	0	1.05	0
LMP1	P07737	PFN1	33391	33391	33391	1	63579	0	0	0	0	0	0.53	0
LMP1	A0A0K0K1H8	HEL-S-71p	24625	24625	24625	1	13271	0	0	0	0	0	1.86	0
LMP1	P08559	PDHA1	55636	55636	55636	1	46251	0	0	0	0	0	1.2	0
LMP1	Q14204	DYNC1H1	65478	65478	65478	1	60281	0	0	0	0	0	1.09	0
LMP1	D3DR37	CEP55	8655	8655	8655	1	42425	0	0	0	0	0	0.2	0
LMP1	Q9H845	ACAD9	58410	58410	58410	1	44433	0	0	0	0	0	1.31	0
LMP1	B4DTK7		26950	26950	26950	1	45191	0	0	0	0	0	0.6	0
LMP1	A0A075B6I0	IGLV8-61	9559	9559	9559	1	36616	0	0	0	0	0	0.26	0
LMP1	A0A024R6W0	GOT2	7980	7980	7980	1	33354	0	0	0	0	0	0.24	0
LMP1	B2RD09		44876	44876	44876	1	65248	0	0	0	0	0	0.69	0
LMP1	A8KAH1		40880	40880	40880	1	35630	0	0	0	0	0	1.15	0
LMP1	Q8IV63	VRK3	21438	21438	21438	1	17644	0	0	0	0	0	1.22	0
LMP1	A0A0S2Z489	PSMD12	18741	18741	18741	1	8535	0	0	0	0	0	2.2	0
LMP1	O43143	DHX15	51419	51419	51419	1	64651	0	0	0	0	0	0.8	0
LMP1	B5BUD2	RFC2	44166	44166	44166	1	26725	0	0	0	0	0	1.65	0
LMP1	Q8TDB6	DTX3L	54524	54524	54524	1	63075	0	0	0	0	0	0.86	0
LMP1	B3KRQ2		49896	49896	49896	1	36511	0	0	0	0	0	1.37	0
LMP1	E9PCR7	OGDH	49367	49367	49367	1	52253	0	0	0	0	0	0.94	0
LMP1	A0A087X1S7	CD48	3478	3478	3478	1	36635	0	0	0	0	0	0.09	0
LMP1	B4E218		31479	31479	31479	1	17885	0	0	0	0	0	1.76	0
LMP1	Q4LE60	TNPO2 variant protein	31975	31975	31975	1	27579	0	0	0	0	0	1.16	0
LMP1	A0A1C7CYX9	DPYSL2	62292	62292	62292	1	55991	0	0	0	0	0	1.11	0
LMP1	P01023	A2M	56458	56458	56458	1	59130	0	0	0	0	0	0.95	0
LMP1	P78406	RAE1	22053	22053	22053	1	21701	0	0	0	0	0	1.02	0
LMP1	Q8TC12	RDH11	38298	38298	38298	1	58568	0	0	0	0	0	0.65	0
LMP1	Q9BW92	TARS2	64822	64822	64822	1	59426	0	0	0	0	0	1.09	0
LMP1	Q86YV0	RASAL3	25011	25011	25011	1	12245	0	0	0	0	0	2.04	0
LMP1	A0A0K0K1L8	HEL-S-129m	38549	38549	38549	1	57286	0	0	0	0	0	0.67	0
LMP1	A5D8X2	ANAPC7	19011	19011	19011	1	56844	0	0	0	0	0	0.33	0
LMP1	D3TTZ3	TNFAIP3	59202	59202	59202	1	58899	0	0	0	0	0	1.01	0
LMP1	A8K5K5	EIF3G	8871	8871	8871	1	4428	0	0	0	0	0	2	0
LMP1	Q6P2Q9	PRPF8	64502	64502	64502	1	56949	0	0	0	0	0	1.13	0
LMP1	Q6R327	RICTOR	15823	15823	15823	1	60050	0	0	0	0	0	0.26	0
LMP1	Q96S55	WRNIP1	13407	13407	13407	1	58552	0	0	0	0	0	0.23	0
LMP1	A8K984		64457	64457	64457	1	36996	0	0	0	0	0	1.74	0
LMP1	Q59ER5		12328	12328	12328	1	14497	0	0	0	0	0	0.85	0
LMP1	P53597	SUCLG1	6876	6876	6876	1	49008	0	0	0	0	0	0.14	0
LMP1	B4DT57		33330	33330	33330	1	35904	0	0	0	0	0	0.93	0
LMP1	A0A0A0MT64	FDXR	12441	12441	12441	1	15803	0	0	0	0	0	0.79	0
LMP1	B4E0E0		15746	15746	15746	1	8853	0	0	0	0	0	1.78	0
LMP1	A0A024R3E3	APOA1	28483	28483	28483	1	32959	0	0	0	0	0	0.86	0
LMP1	Q6NUK7	LYN	17315	17315	17315	1	47306	0	0	0	0	0	0.37	0
LMP1	O43615	TIMM44	37306	37306	37306	1	45828	0	0	0	0	0	0.81	0
LMP1	Q96I99	SUCLG2	1427	1427	1427	1	15031	0	0	0	0	0	0.09	0
LMP1	A8K6U2		18449	18449	18449	1	12260	0	0	0	0	0	1.5	0
LMP1	Q15084	PDIA6	63313	63313	63313	1	32116	0	0	0	0	0	1.97	0
LMP1	A0A024R9Y7	MAGED2	52180	52180	52180	1	22575	0	0	0	0	0	2.31	0
LMP1	P99999	CYCS	31309	31309	31309	1	32655	0	0	0	0	0	0.96	0
LMP1	Q9UL90		49255	49255	49255	1	30277	0	0	0	0	0	1.63	0
LMP1	B2R7P8		15092	15092	15092	1	59977	0	0	0	0	0	0.25	0

LMP1	Q92538	GBF1	62204	62204	62204	1	36035	0	0	0	0	0	1.73	0
LMP1	Q5VYY1	ANKRD22	38711	38711	38711	1	50799	0	0	0	0	0	0.76	0
LMP1	A0A0X9SZU9		3089	3089	3089	1	55588	0	0	0	0	0	0.06	0
LMP1	A8K5S3		49300	49300	49300	1	57679	0	0	0	0	0	0.85	0
LMP1	Q7Z7L1	SLFN11	46621	46621	46621	1	55878	0	0	0	0	0	0.83	0
LMP1	P47989	XDH	19529	19529	19529	1	27897	0	0	0	0	0	0.7	0
LMP1	Q9UBS4	DNAJB11	39338	39338	39338	1	42937	0	0	0	0	0	0.92	0
LMP1	P07741	APRT	49785	49785	49785	1	40008	0	0	0	0	0	1.24	0
LMP1	A0A024QZY1	JTV1	35401	35401	35401	1	51667	0	0	0	0	0	0.69	0
LMP1	A0A087X0V5	OAS2	665	665	665	1	49592	0	0	0	0	0	0.01	0
LMP1	A0A024R8W0	DDX48	31513	31513	31513	1	62992	0	0	0	0	0	0.5	0
LMP1	P43246	MSH2	43510	43510	43510	1	21442	0	0	0	0	0	2.03	0
LMP1	A0A0G2JIW1	HSPA1B	38136	38136	38136	1	49441	0	0	0	0	0	0.77	0
LMP1	A0A0S2Z410	HSD17B10	55698	55698	55698	1	55236	0	0	0	0	0	1.01	0
LMP1	Q96L58	B3GALT6	35836	35836	35836	1	47759	0	0	0	0	0	0.75	0
LMP1	Q99973	TEP1	4096	4096	4096	1	22495	0	0	0	0	0	0.18	0
LMP1	Q9Y3D0	CIAO2B	62850	62850	62850	1	49748	0	0	0	0	0	1.26	0
LMP1	Q04695	KRT17	51943	51943	51943	1	62127	0	0	0	0	0	0.84	0
LMP1	Q9H1I8	ASCC2	45775	45775	45775	1	19172	0	0	0	0	0	2.39	0
LMP1	A0A087WUL9	PSMD13	32571	32571	32571	1	24975	0	0	0	0	0	1.3	0
LMP1	Q96D53	COQ8B	24921	24921	24921	1	18345	0	0	0	0	0	1.36	0
LMP1	P23526	AHCY	40160	40160	40160	1	55764	0	0	0	0	0	0.72	0
LMP1	P33527	ABCC1	55227	55227	55227	1	29800	0	0	0	0	0	1.85	0
LMP1	A0A0S2Z2Z6	ANXA6	3006	3006	3006	1	37281	0	0	0	0	0	0.08	0
LMP1	A0JP11	PIK3R4	59674	59674	59674	1	29195	0	0	0	0	0	2.04	0
LMP1	Q9NW13	RBM28	16178	16178	16178	1	19320	0	0	0	0	0	0.84	0
LMP1	O94822	LTN1	53070	53070	53070	1	46798	0	0	0	0	0	1.13	0
LMP1	B2R623		18540	18540	18540	1	28891	0	0	0	0	0	0.64	0
LMP1	A0A125QYY4		15360	15360	15360	1	64840	0	0	0	0	0	0.24	0
LMP1	Q8IXI1	RHOT2	56895	56895	56895	1	59353	0	0	0	0	0	0.96	0
LMP1	A8K486		75	75	75	1	64213	0	0	0	0	0	0	0
LMP1	A0A0A0MTH3	ILK	30599	30599	30599	1	54074	0	0	0	0	0	0.57	0
LMP1	Q4LE36	ACLY variant protein	21215	21215	21215	1	38518	0	0	0	0	0	0.55	0
LMP1	B5BU38	ANXA1	58704	58704	58704	1	64508	0	0	0	0	0	0.91	0
LMP1	P54886	ALDH18A1	49504	49504	49504	1	43720	0	0	0	0	0	1.13	0
LMP1	A8K4T6		31663	31663	31663	1	33516	0	0	0	0	0	0.94	0
LMP1	B3KNC3		5478	5478	5478	1	9308	0	0	0	0	0	0.59	0
