# Supplementary material for: LMP1 enhances aerobic glycolysis in natural killer/T cell lymphoma
Source: Cell Death Dis. 2024 Aug 20;15(8):604. doi: 10.1038/s41419-024-06999-7 (PMC11335758; doi:10.1038/s41419-024-06999-7)
Supplement: Supplementary file 4 — Additional File 4 [file 41419_2024_6999_MOESM4_ESM.pdf]

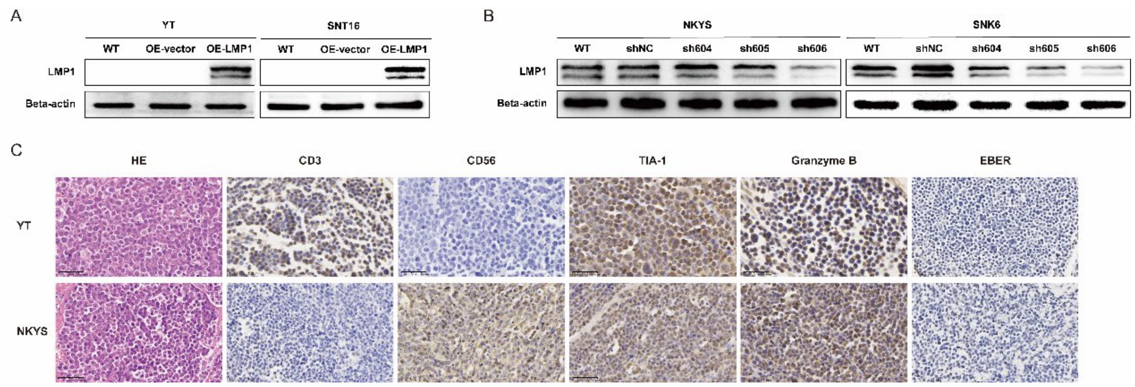

Figure S1. Construction of stable LMP1 overexpression and LMP1 knockdown cells and establishment of NKTCL xenograft mouse models.

A. LMP1 expression was examined on OE-LMP1 cells (YT, SNT16) by western blotting.

B. LMP1 expression was examined on shLMP1 cells (NKYS, SNK6) by western blotting.

C. Representative HE staining and IHC staining of CD3, CD56, TIA-1, and Granzyme B, and EBER in situ hybridization in the NKTCL xenograft tumor tissues (400X).

NKTCL, natural killer/T cell lymphoma; HE, hematoxylin-eosin; IHC, immunohistochemistry; EBER, Epstein-Barr virus-encoded RNA.

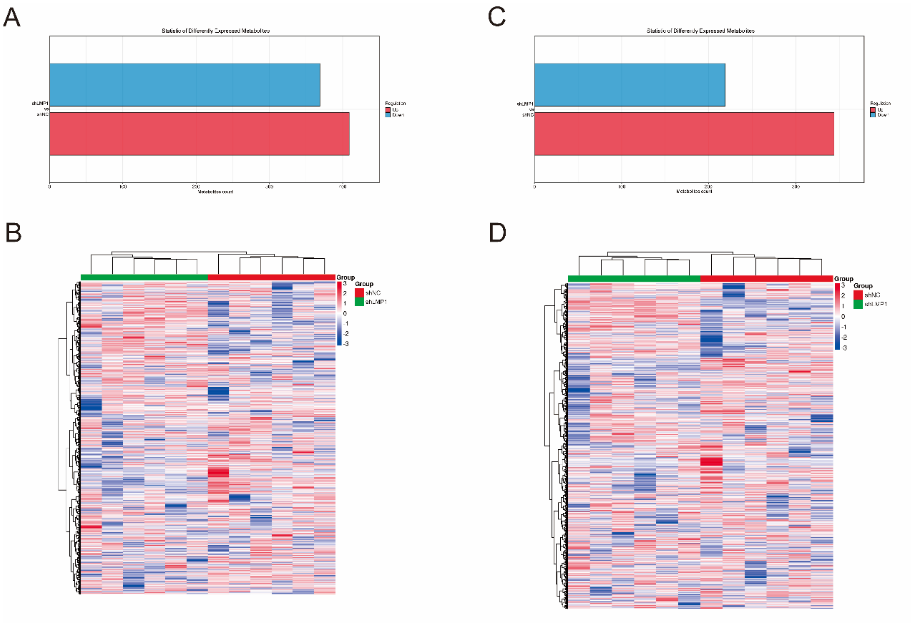

Figure S2. Untargeted metabolomic sequencing between NKYS^{shNC} and NKYS^{shLMP1} cells.

A. Statistic of differentially expressed metabolites of in positive mode.

B. Hierarchical Clustering heat map of differentially expressed metabolites of in positive mode.

C. Statistic of differentially expressed metabolites of in negative mode.

D. Hierarchical Clustering heat map of differentially expressed metabolites of in negative mode.

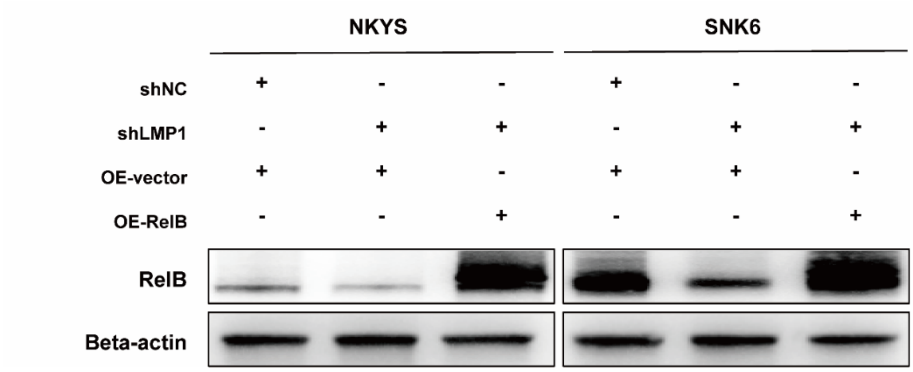

Figure S3. Construction of stable RelB overexpression and RelB knockdown in NKYS and SNK6 cells.

RelB expression was examined by western blotting
