# Supplementary material for: LMP1 enhances aerobic glycolysis in natural killer/T cell lymphoma
Source: Cell Death Dis. 2024 Aug 20;15(8):604. doi: 10.1038/s41419-024-06999-7 (PMC11335758; doi:10.1038/s41419-024-06999-7)

Figure 1A

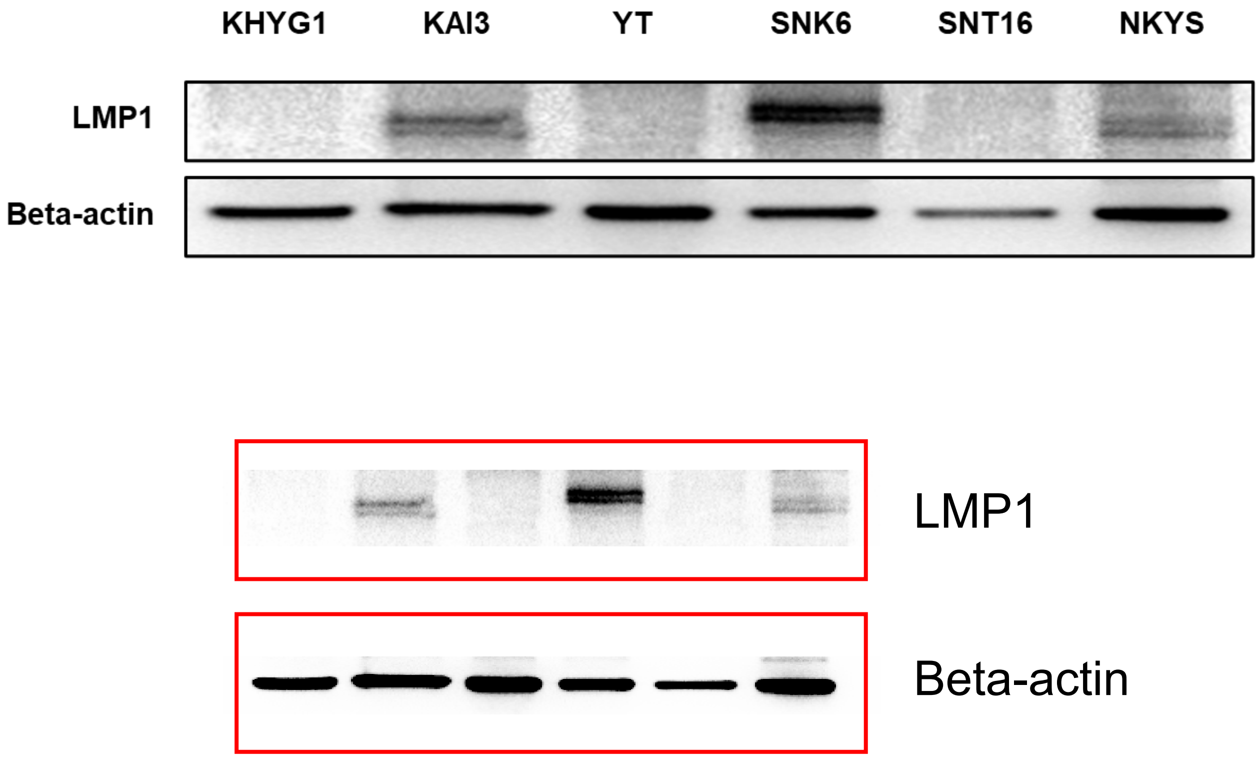

KHYG1
KAI3
YT
SNK6
SNT16
NKYS
LMP1
Beta-actin
LMP1
Beta-actin

Figure 2H-YT

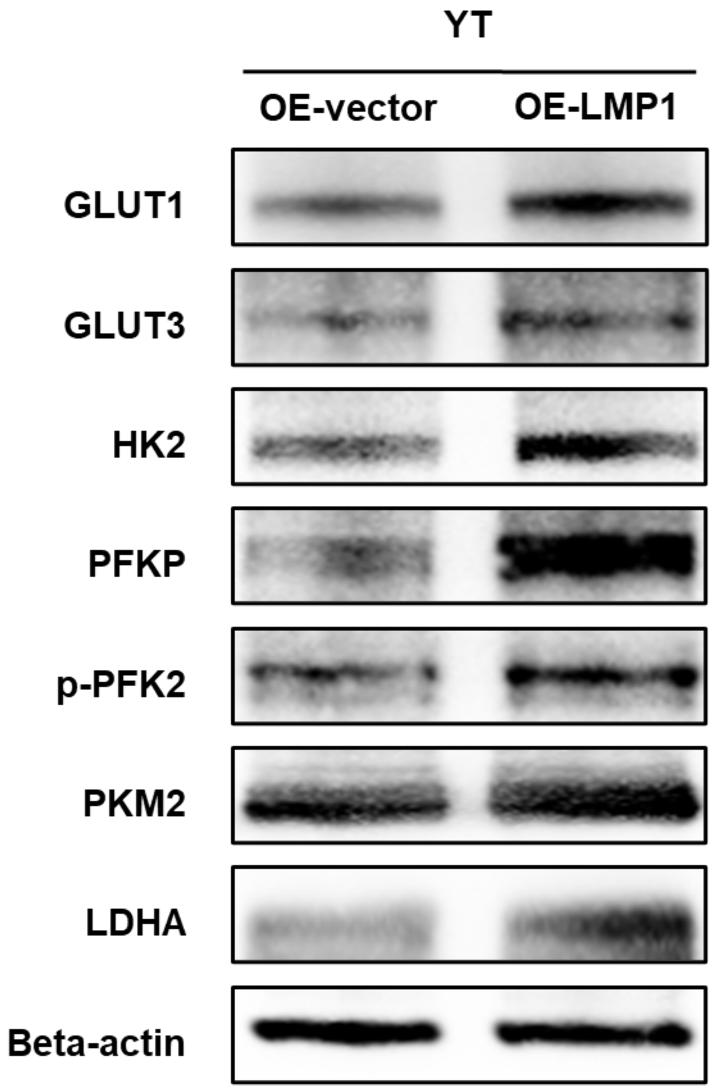

YT
OE-vector
OE-LMP1
GLUT1
GLUT3
HK2
PFKP
p-PFK2
PKM2
LDHA
Beta-actin

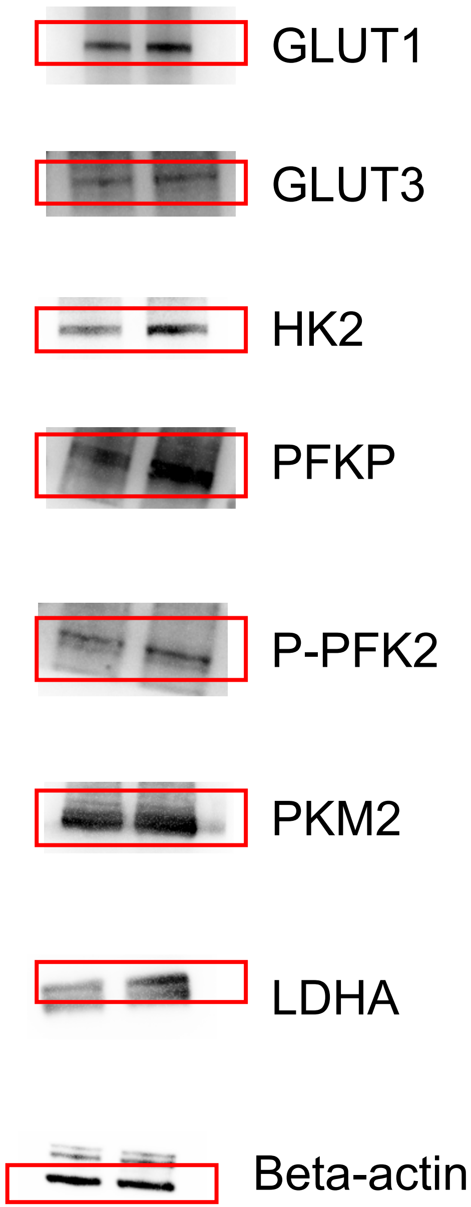

GLUT1
GLUT3
HK2
PFKP
P-PFK2
PKM2
LDHA
Beta-actin

Figure 2H-SNT16

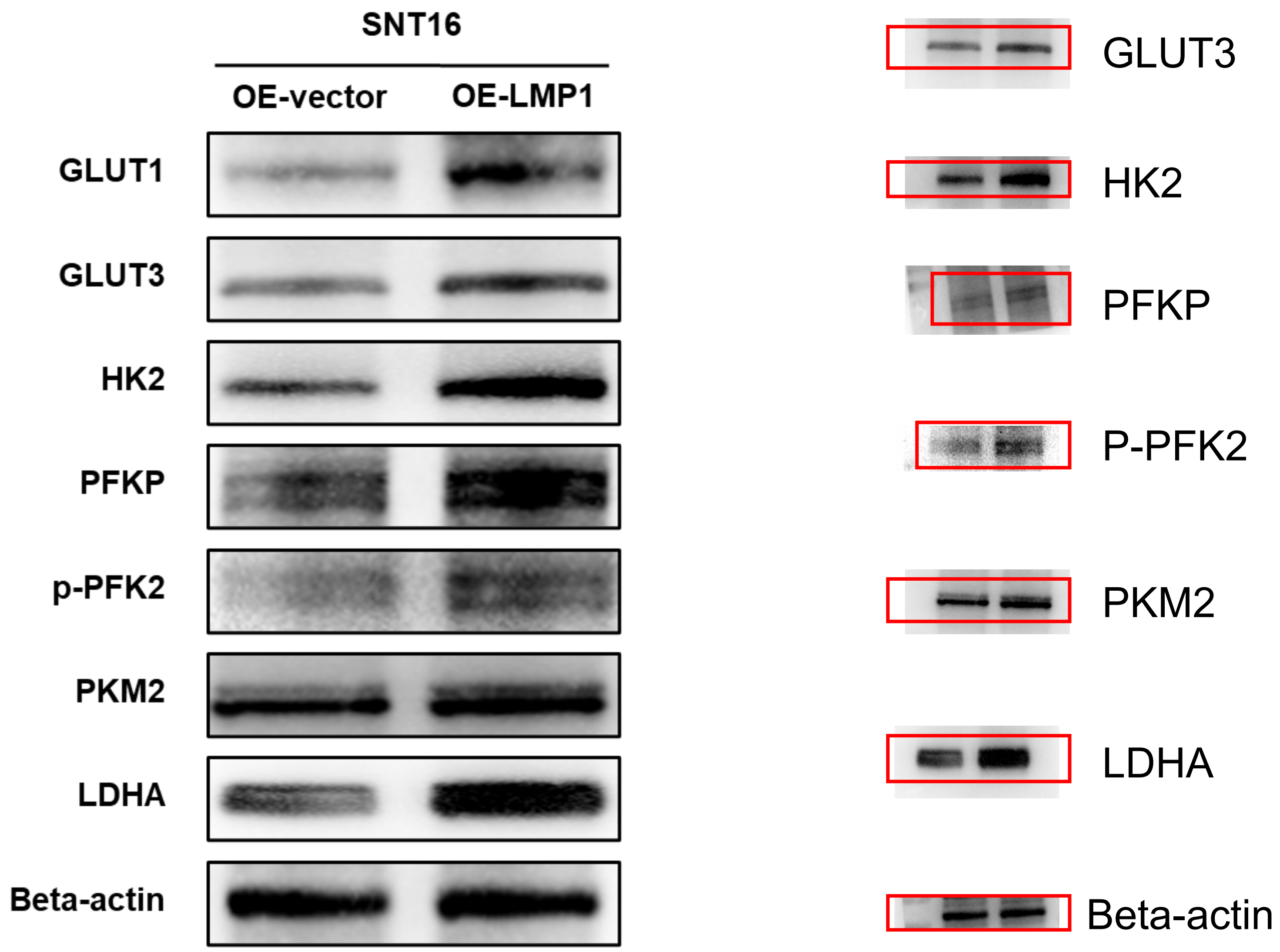

Figure 2H-NKYS

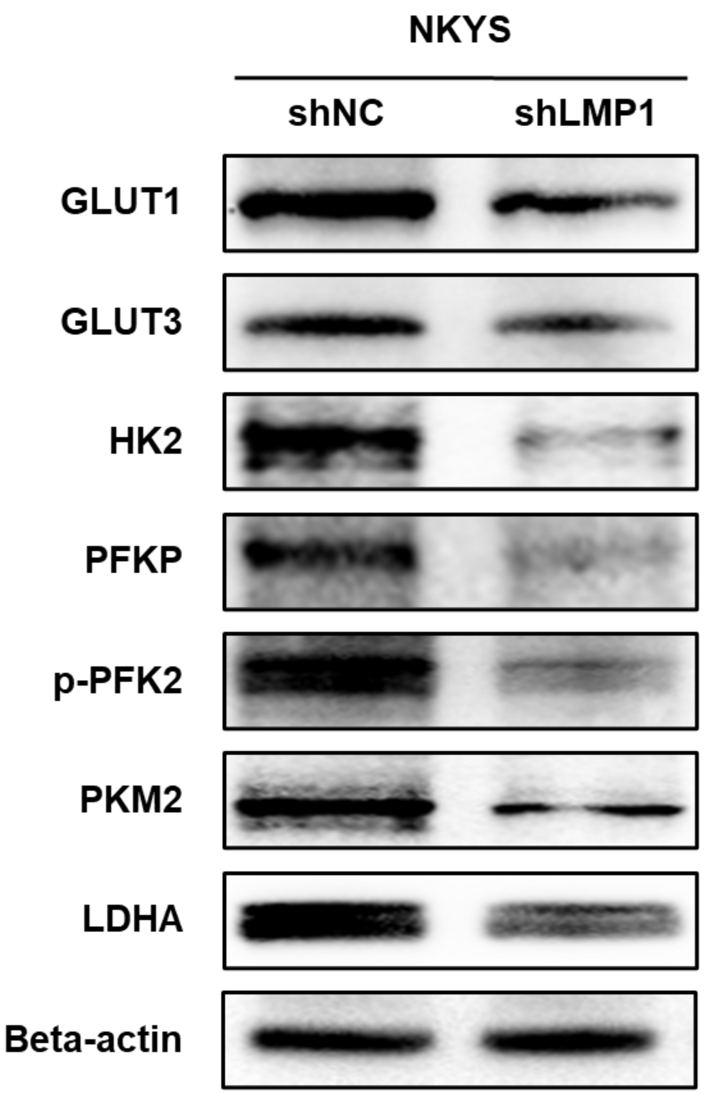

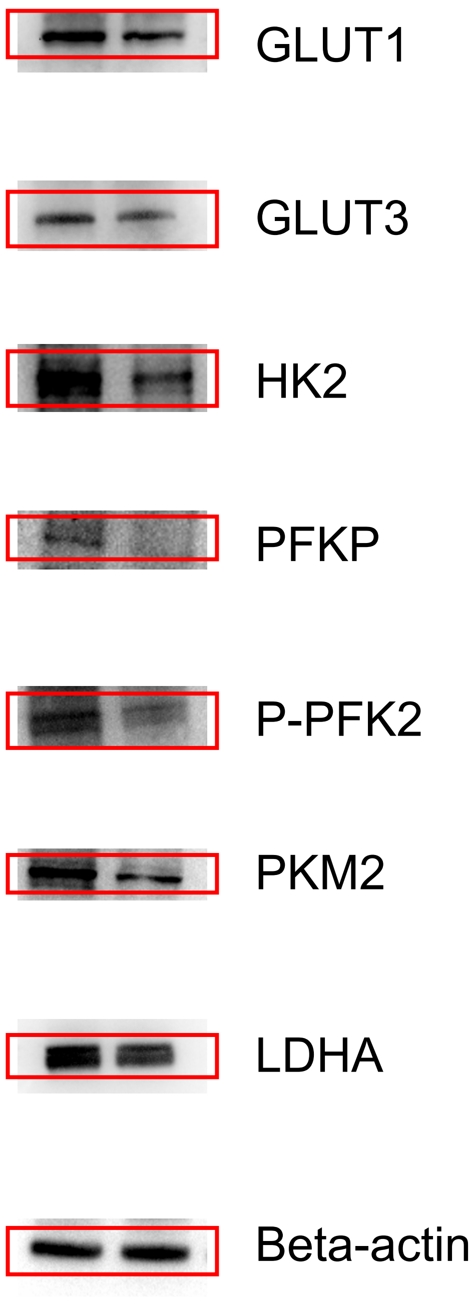

Figure 2H-SNK6

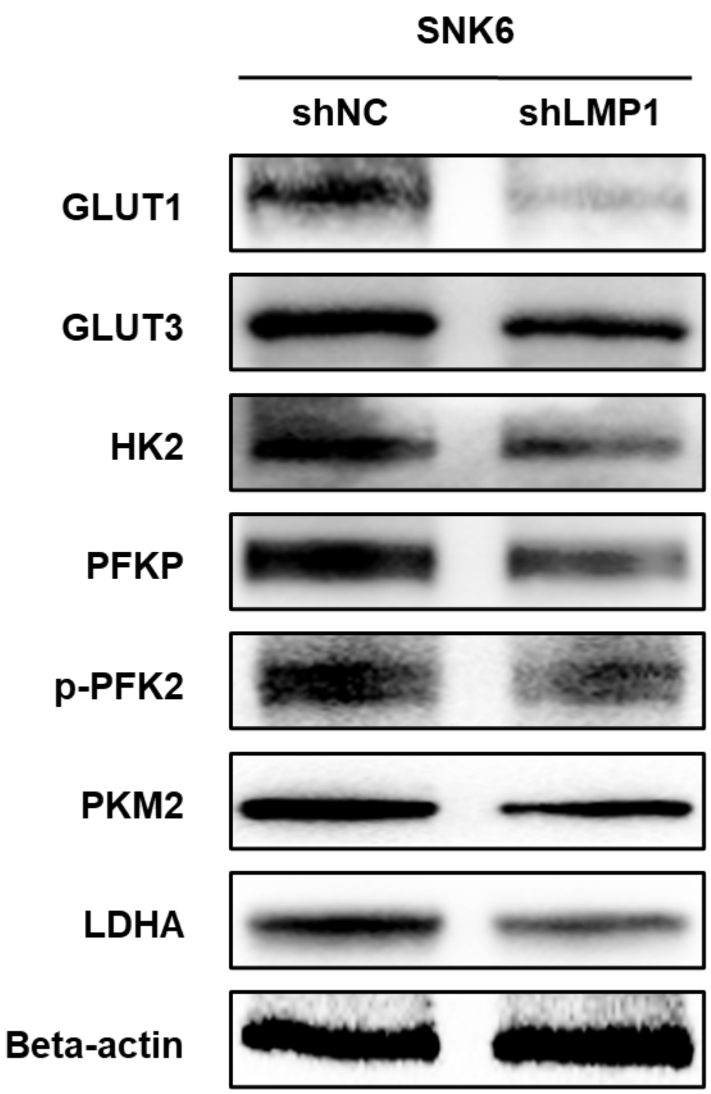

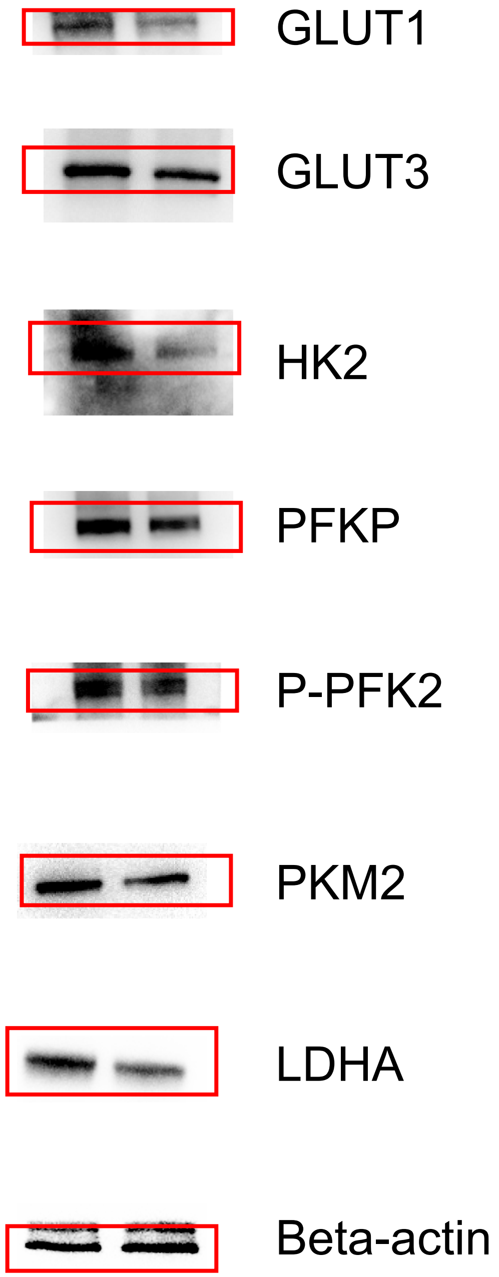

Figure 3C-NKYS

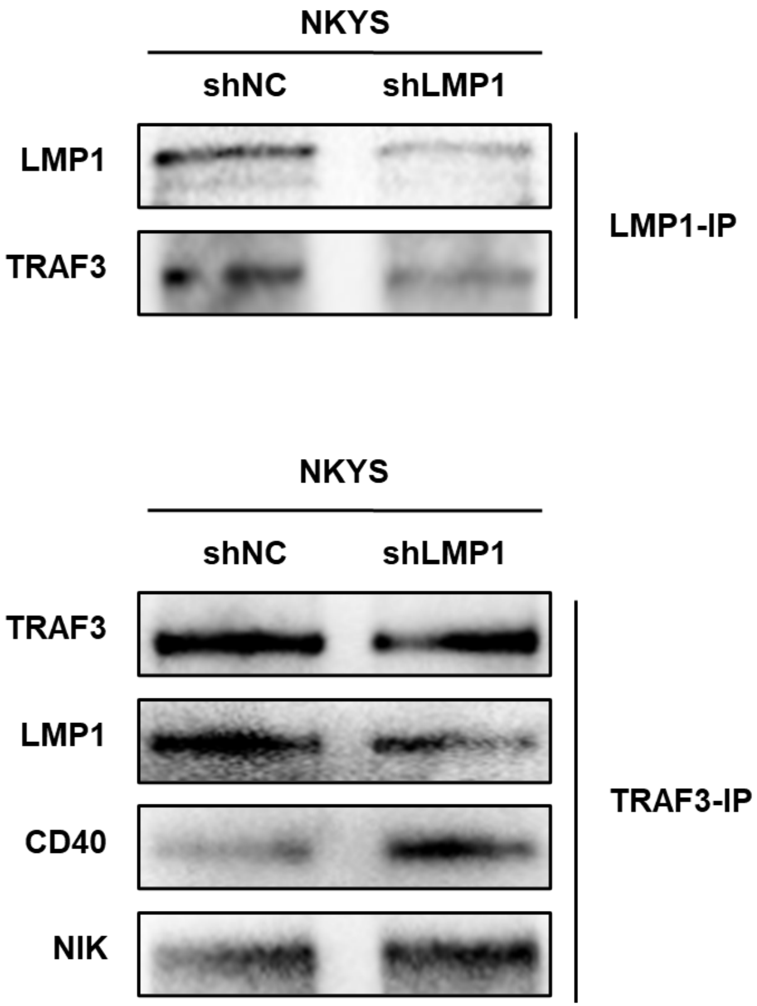

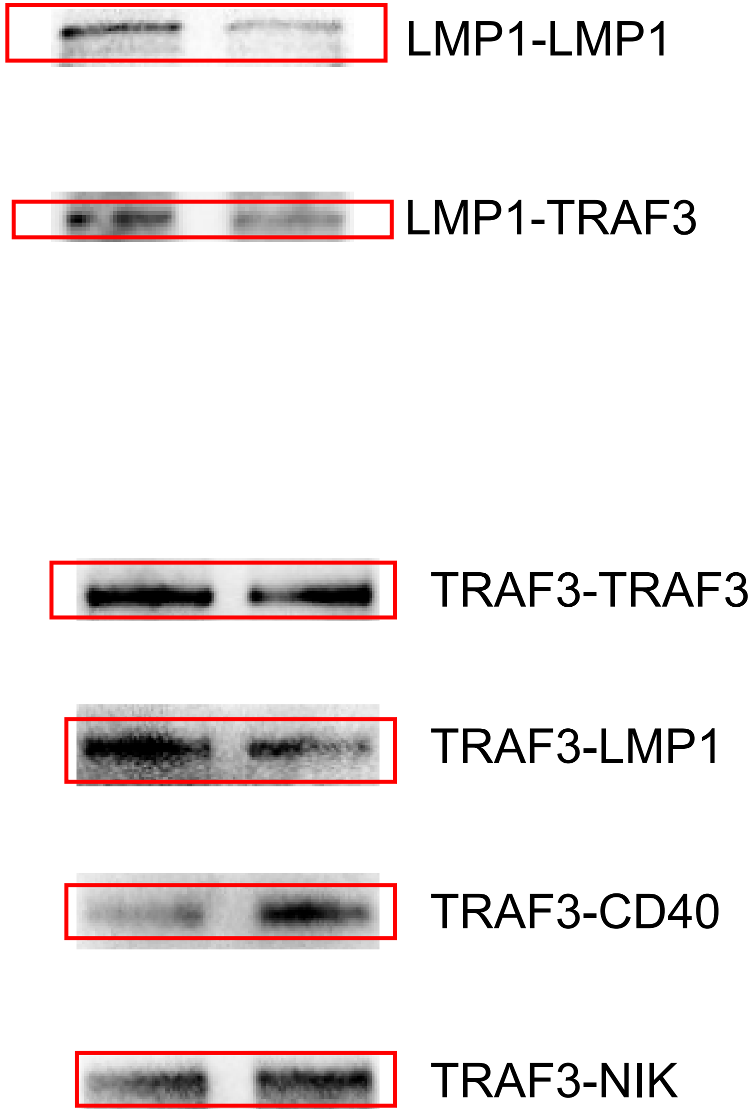

Figure 3C-SNK6

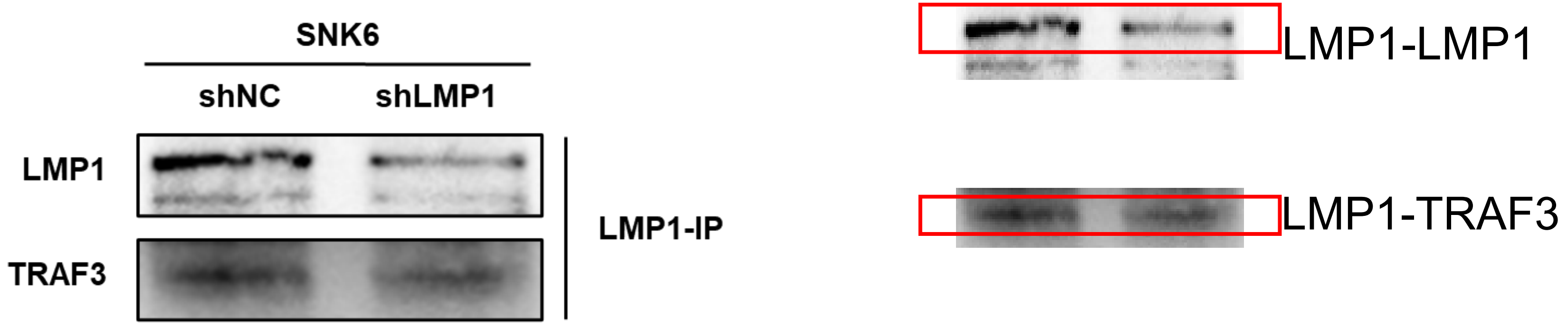

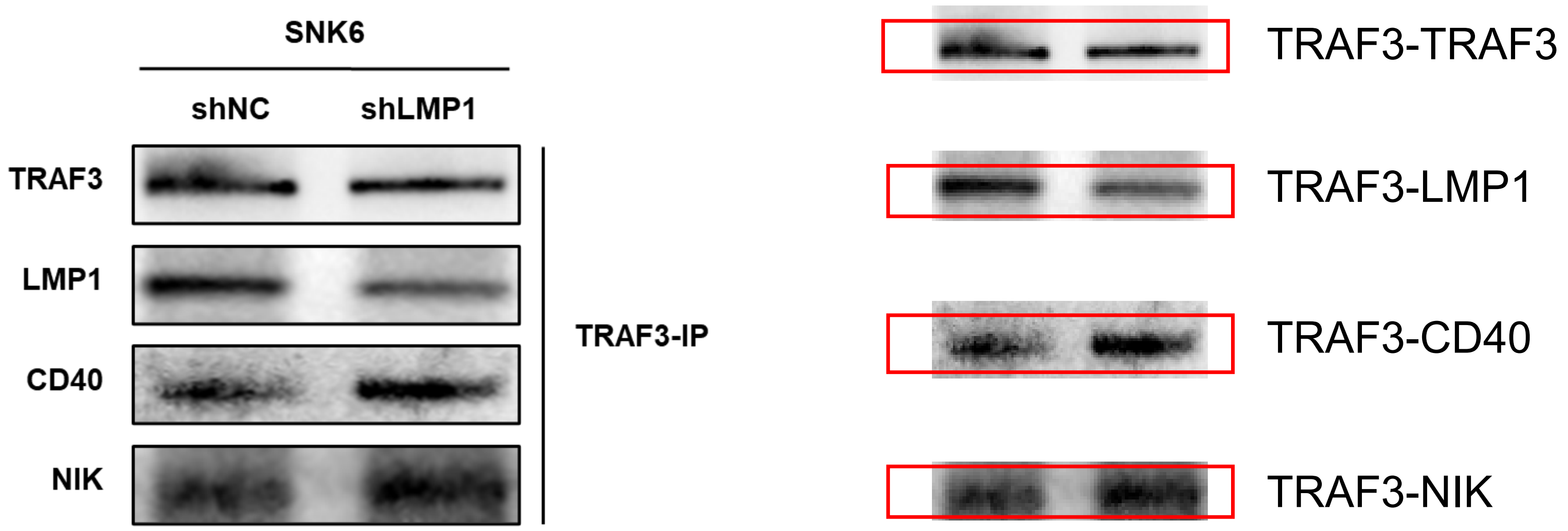

Figure 3E-YT

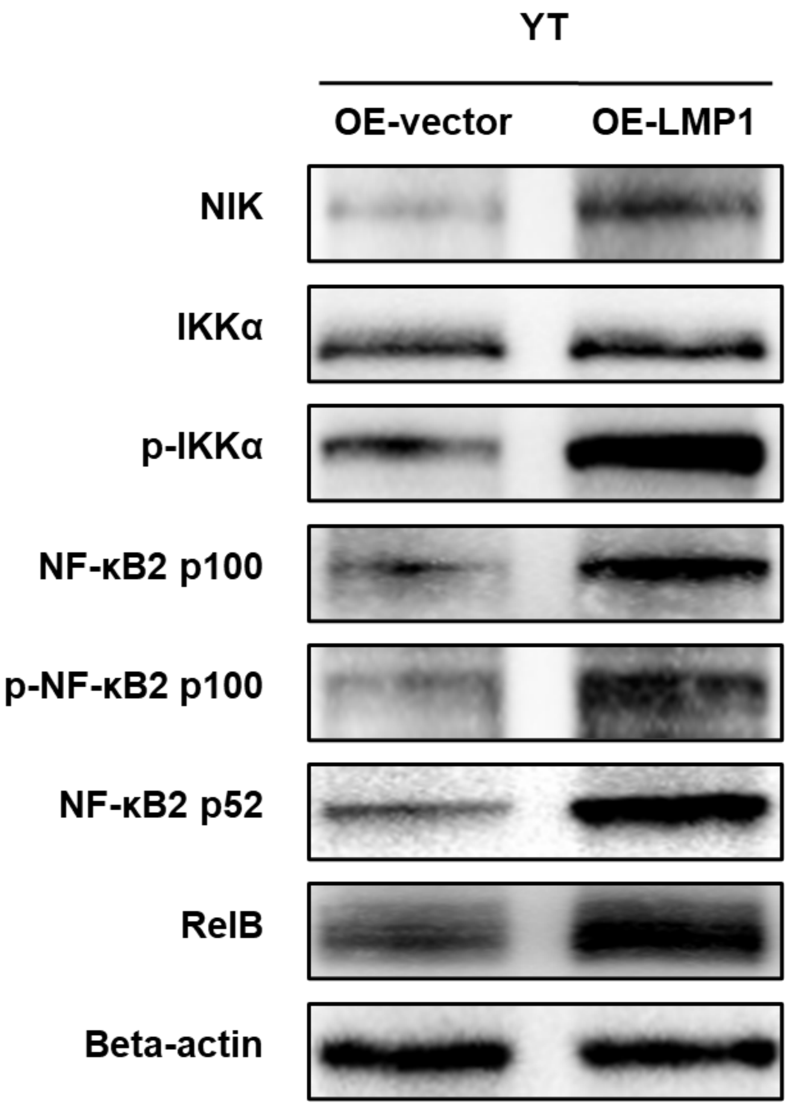
YT
OE-vector
OE-LMP1
NIK
IKKα
p-IKKα
NF-κB2 p100
p-NF-κB2 p100
NF-κB2 p52
RelB
Beta-actin

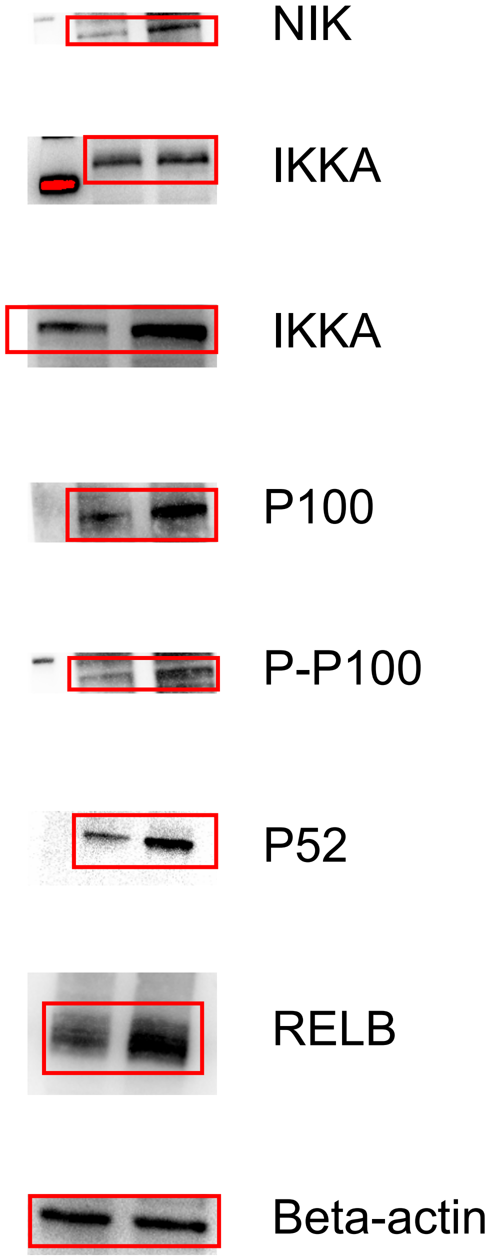
NIK
IKKA
IKKA
P100
P-P100
P52
RELB
Beta-actin

Figure 3E-SNT16

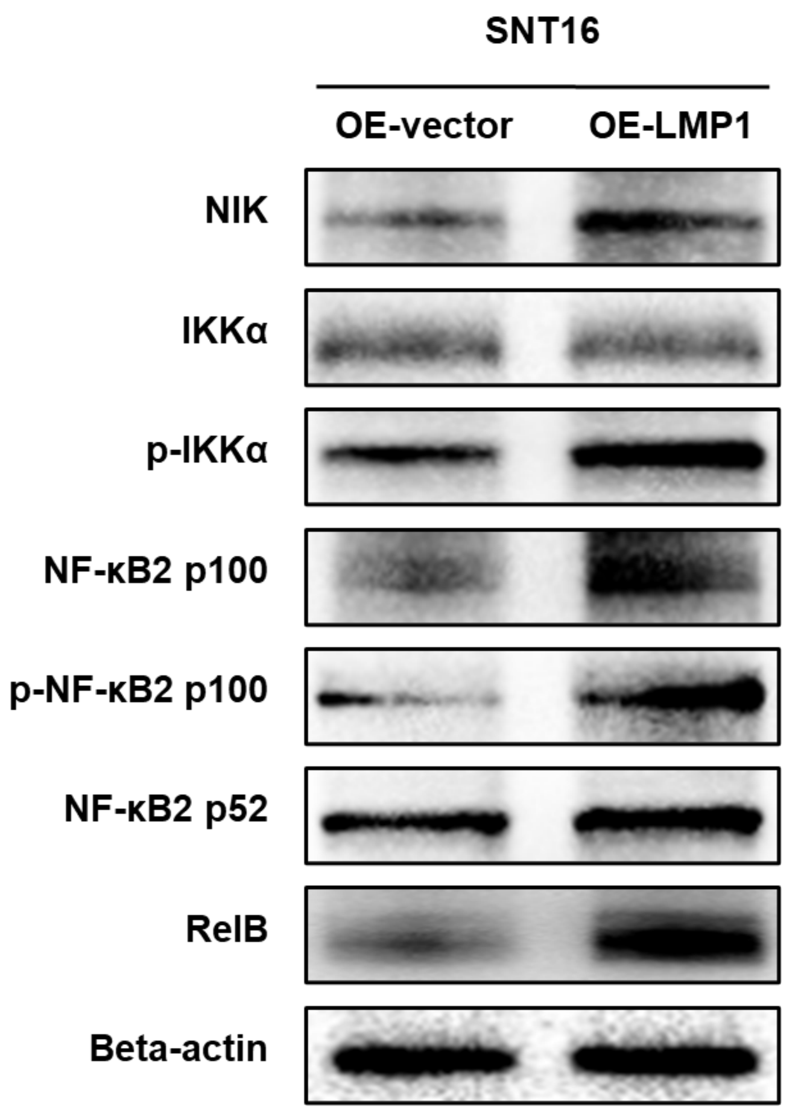

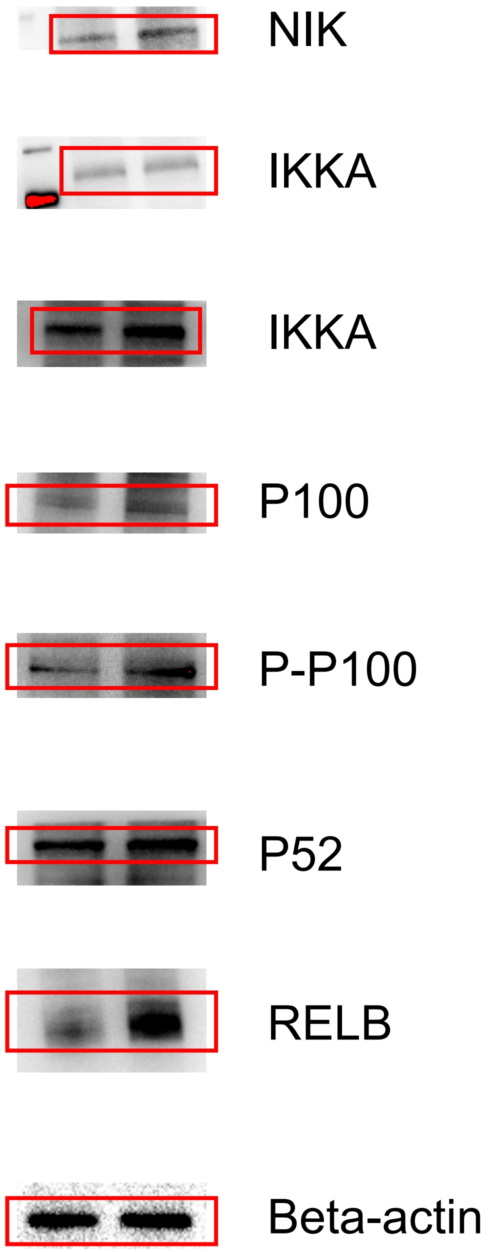

Figure 3E-NKYS

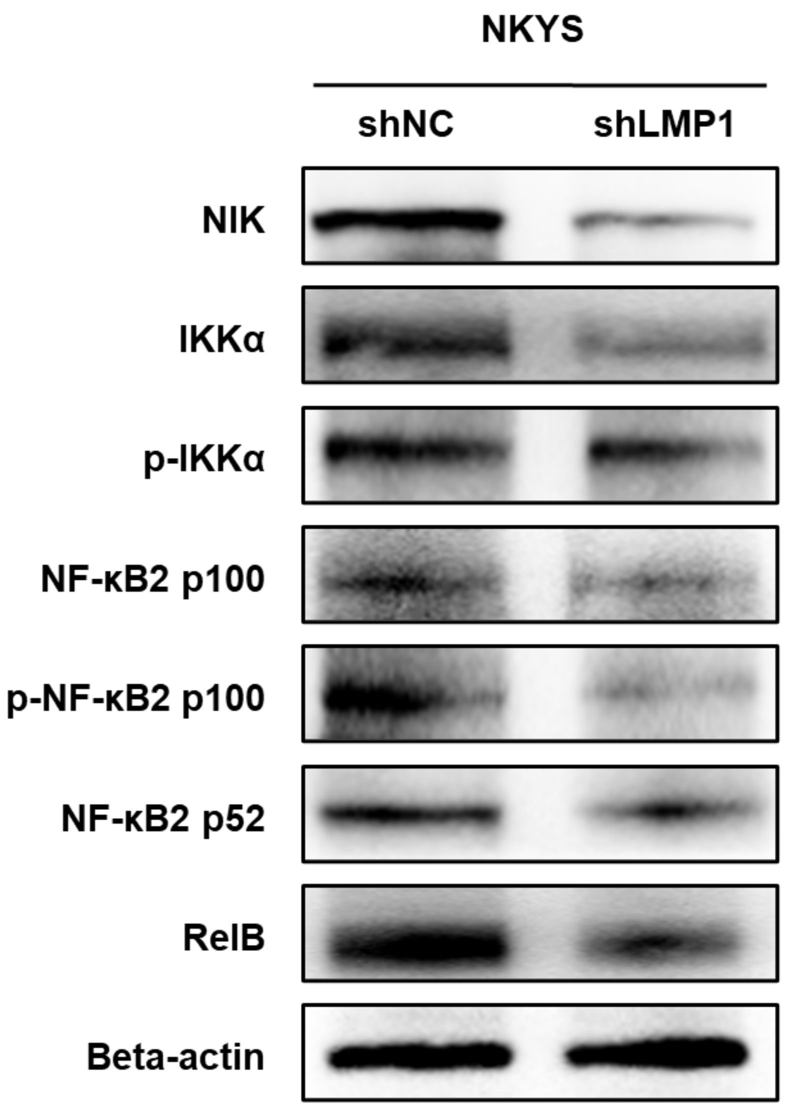

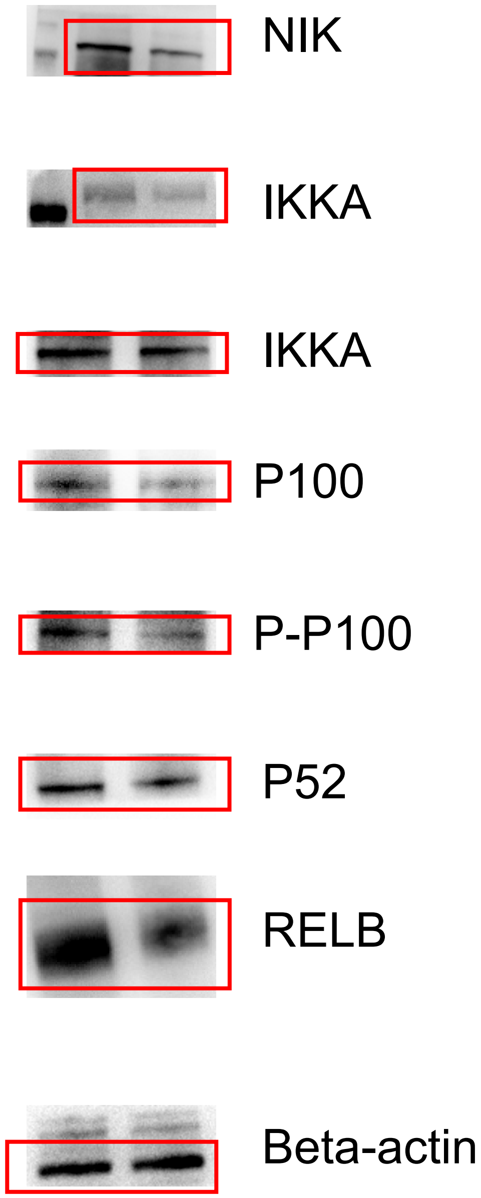

Figure 3E-SNK6

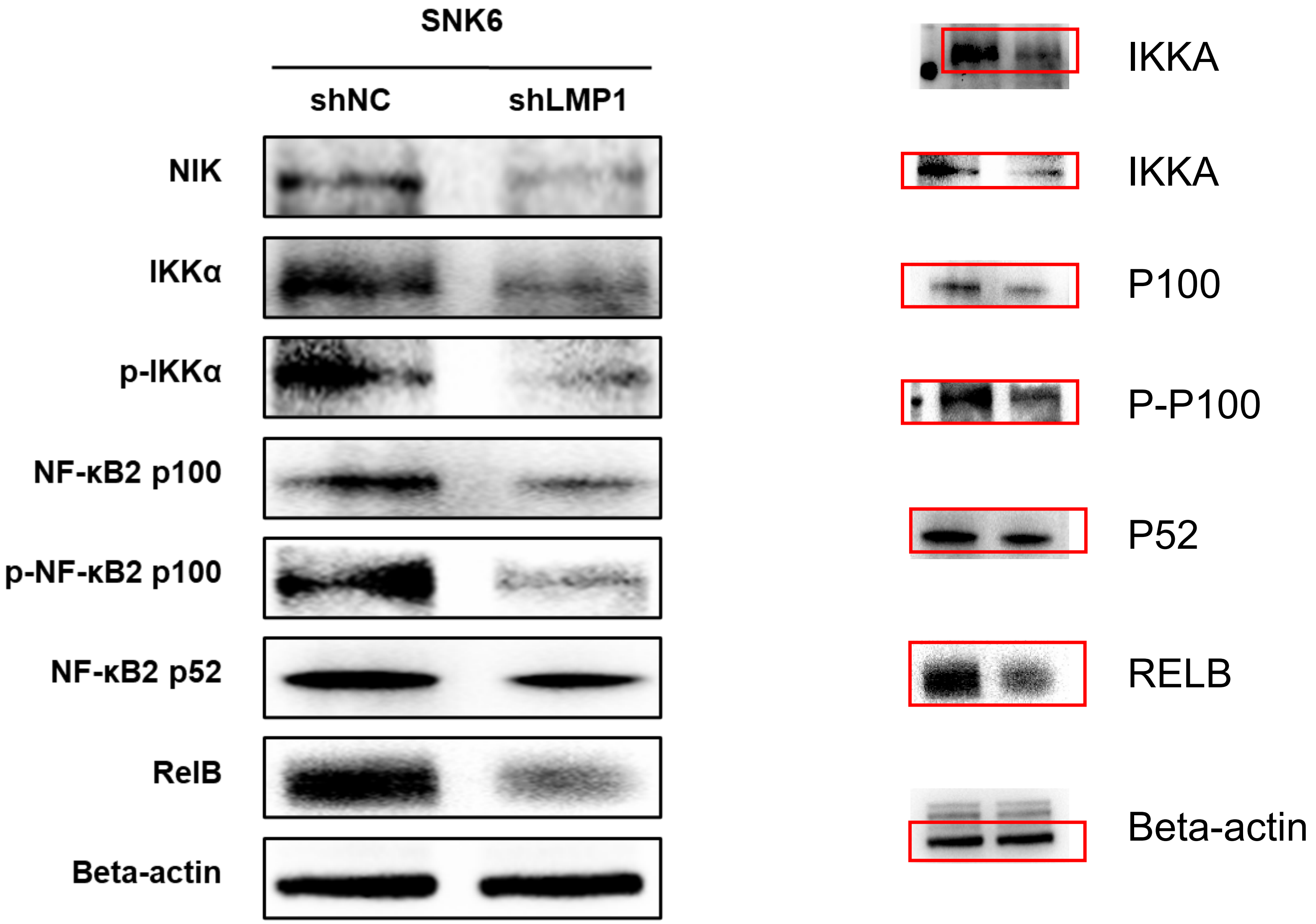

Figure 5I-YT

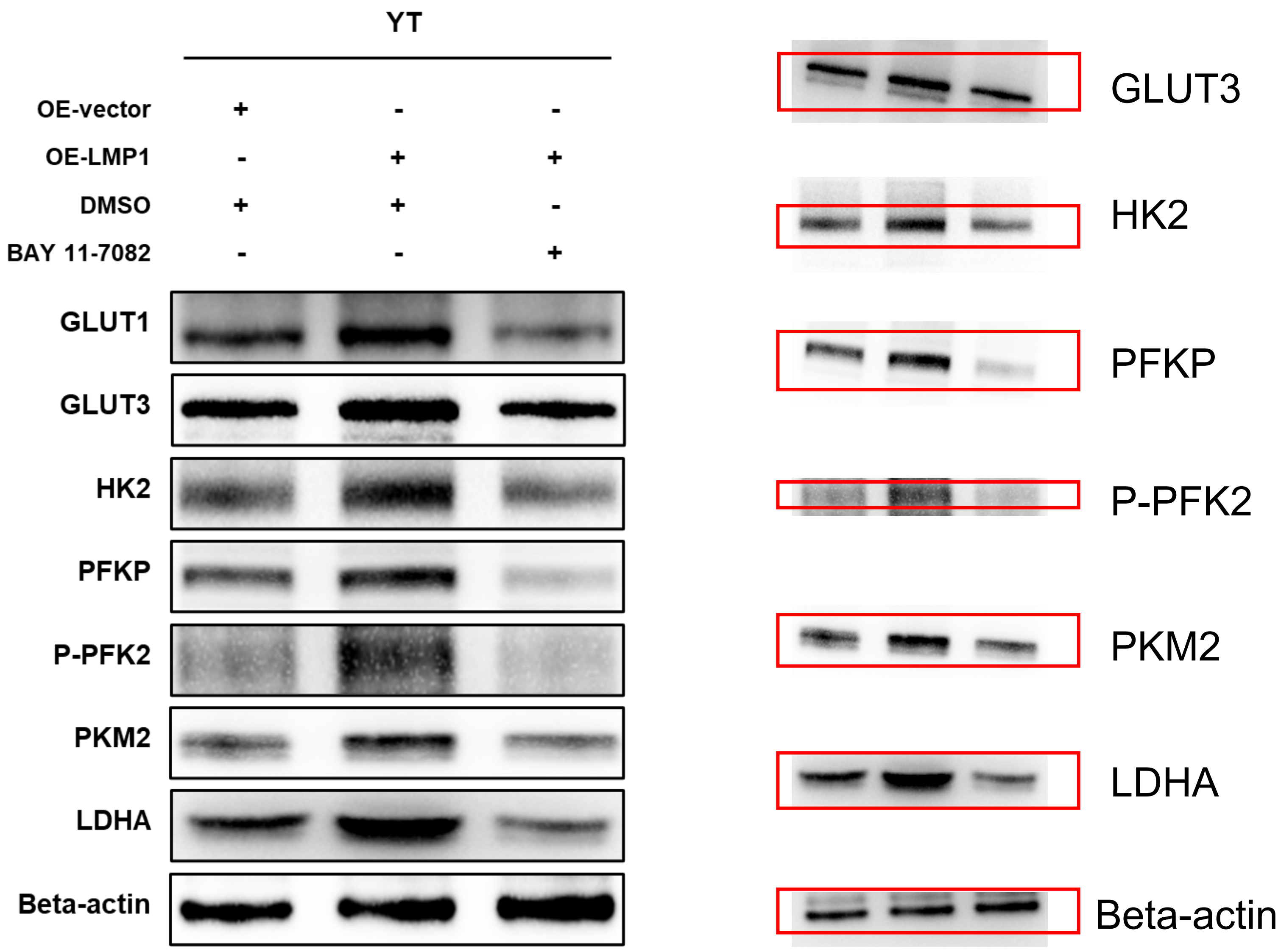
YT
OE-vector + - -
OE-LMP1 - + +
DMSO + + -
BAY 11-7082 - - +
GLUT1
GLUT3
HK2
PFKP
P-PFK2
PKM2
LDHA
Beta-actin
GLUT1
GLUT3
HK2
PFKP
P-PFK2
PKM2
LDHA
Beta-actin

Figure 5I-SNT16

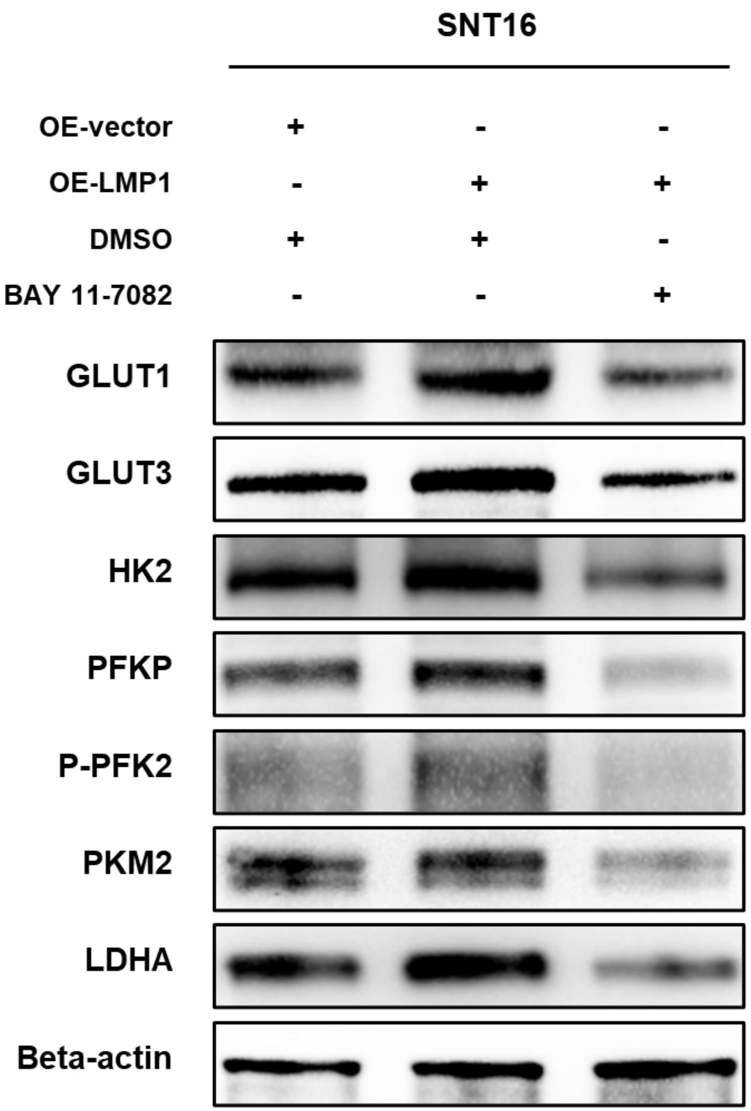

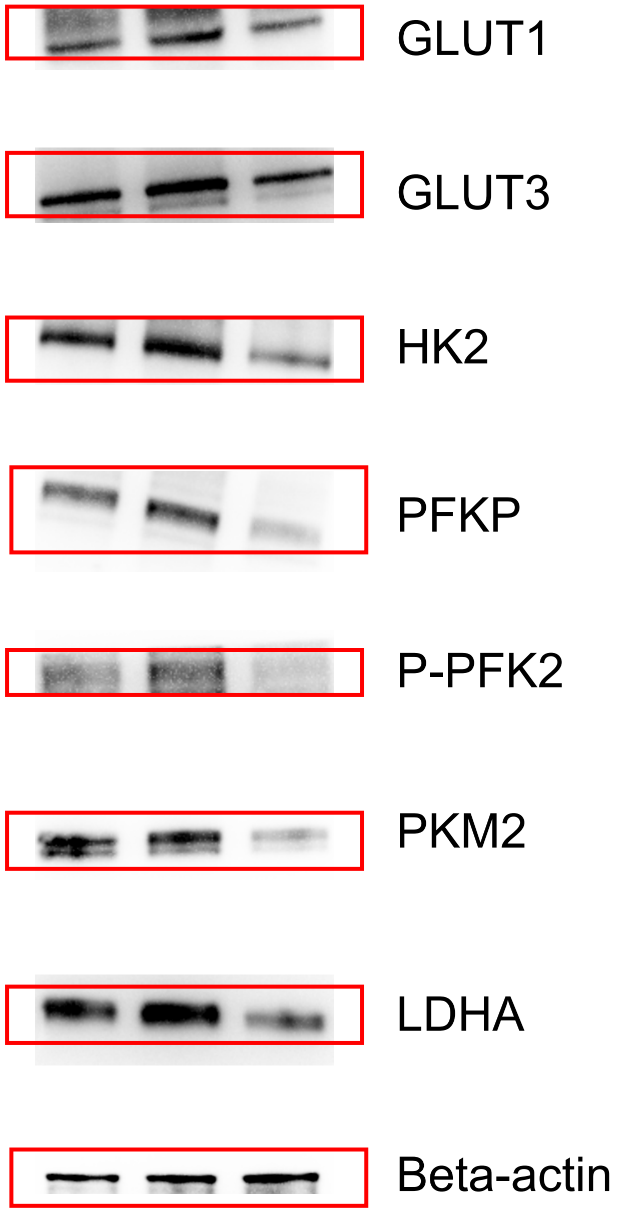

Figure 5J-NKYS

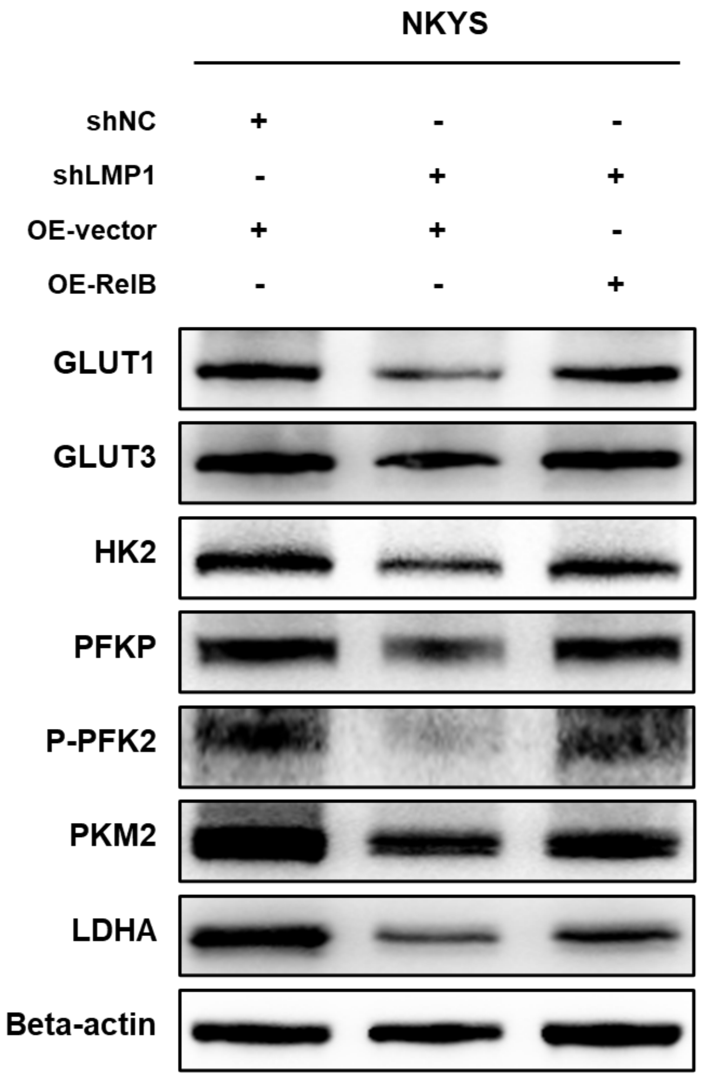

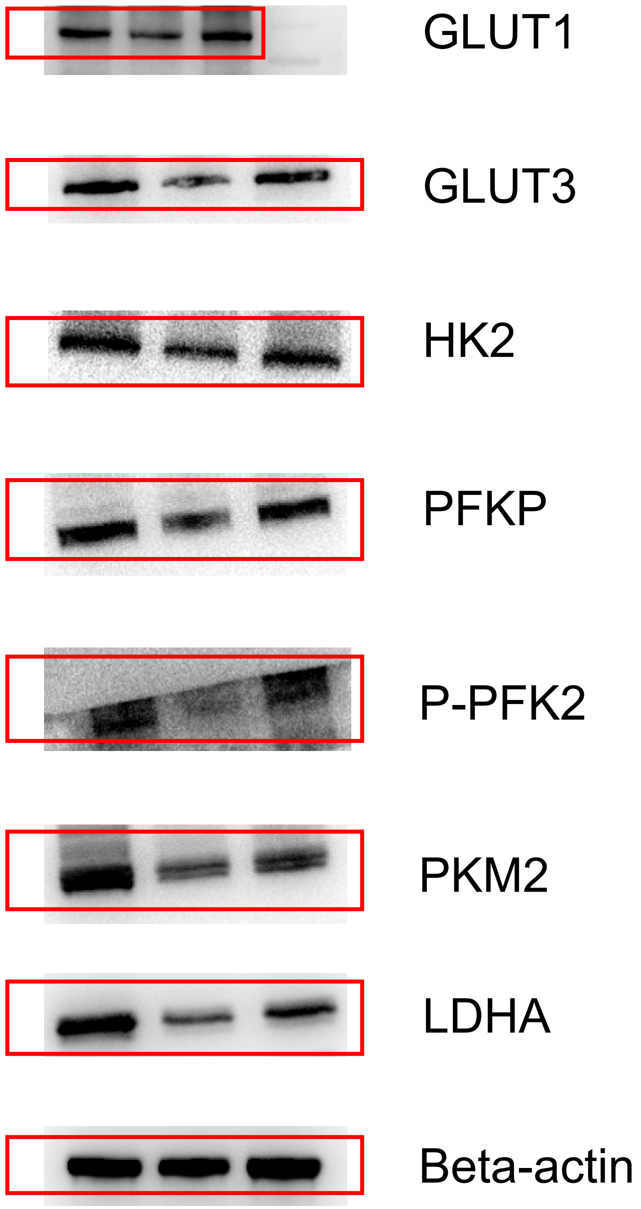

Figure 5J-SNK6

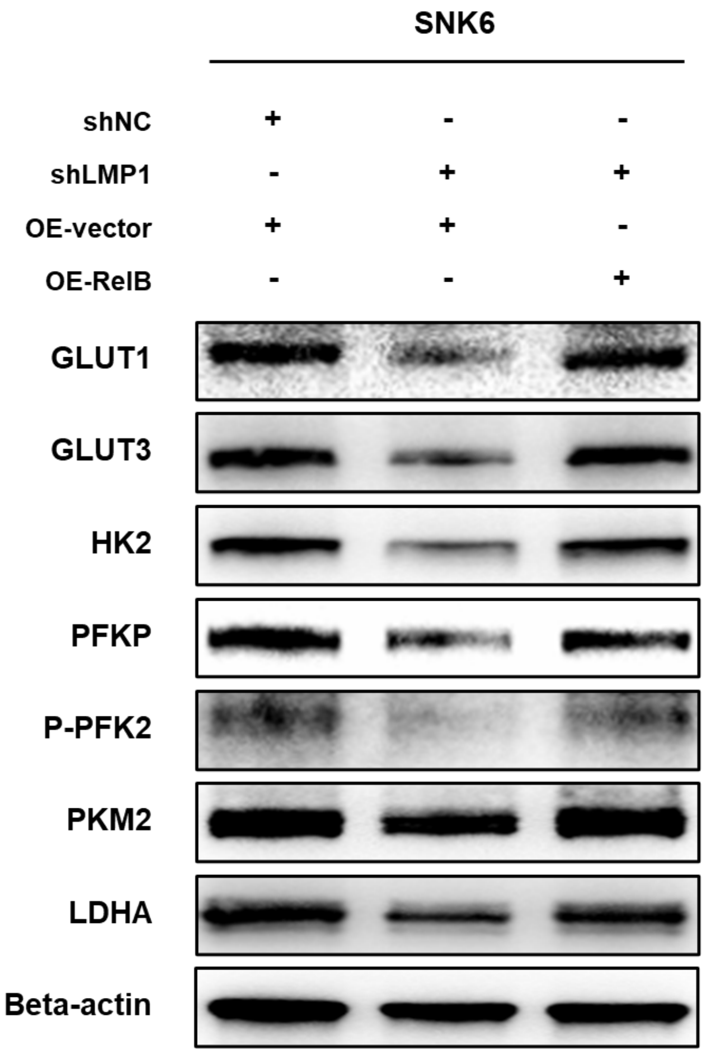

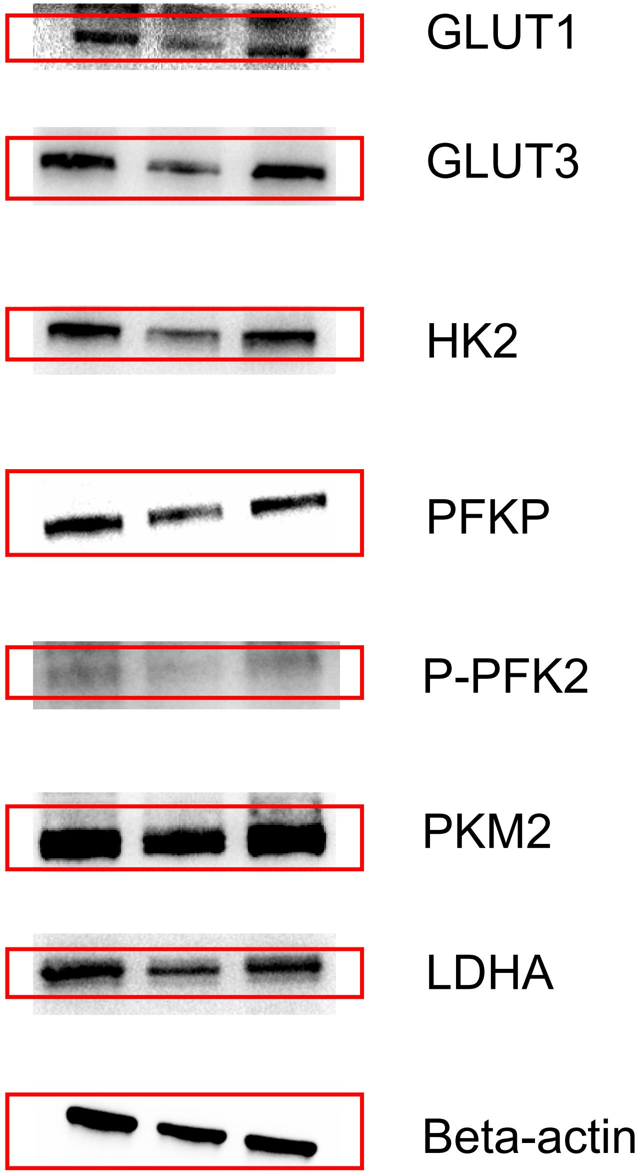

Additional file 4: Figure S1A

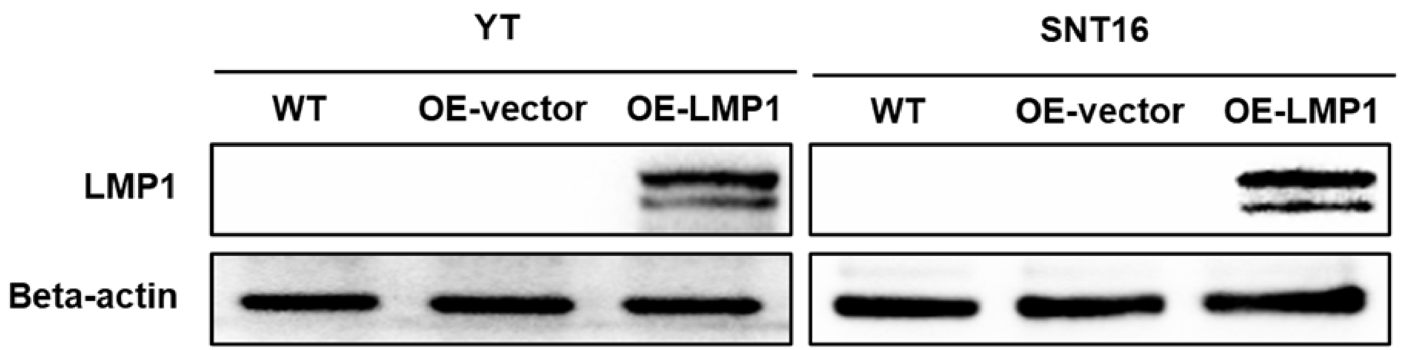

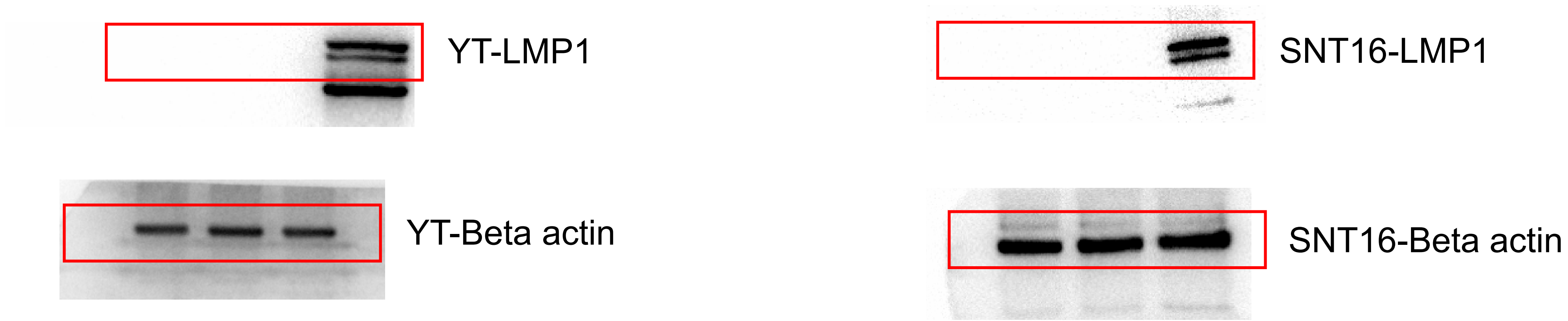

Additional file 4: Figure S1B

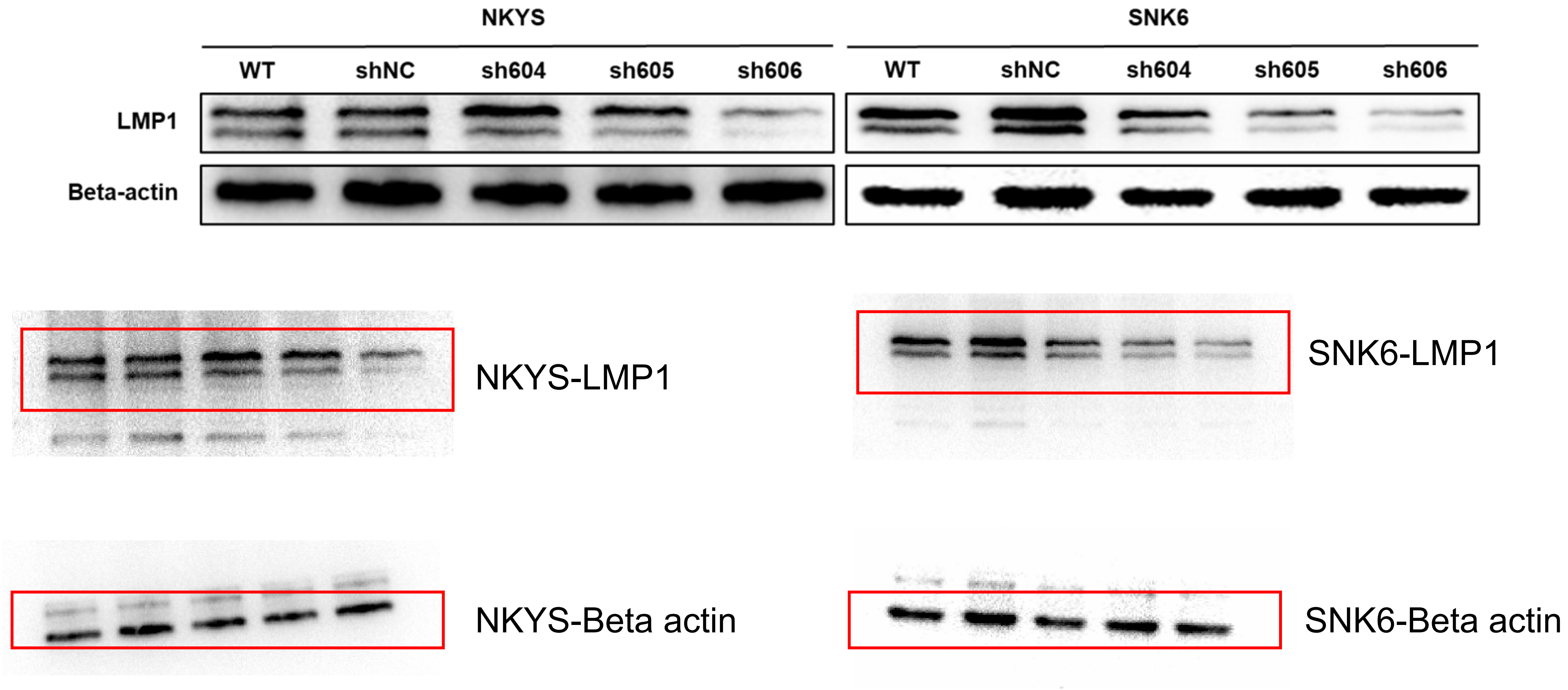

Additional file 4: Figure S3

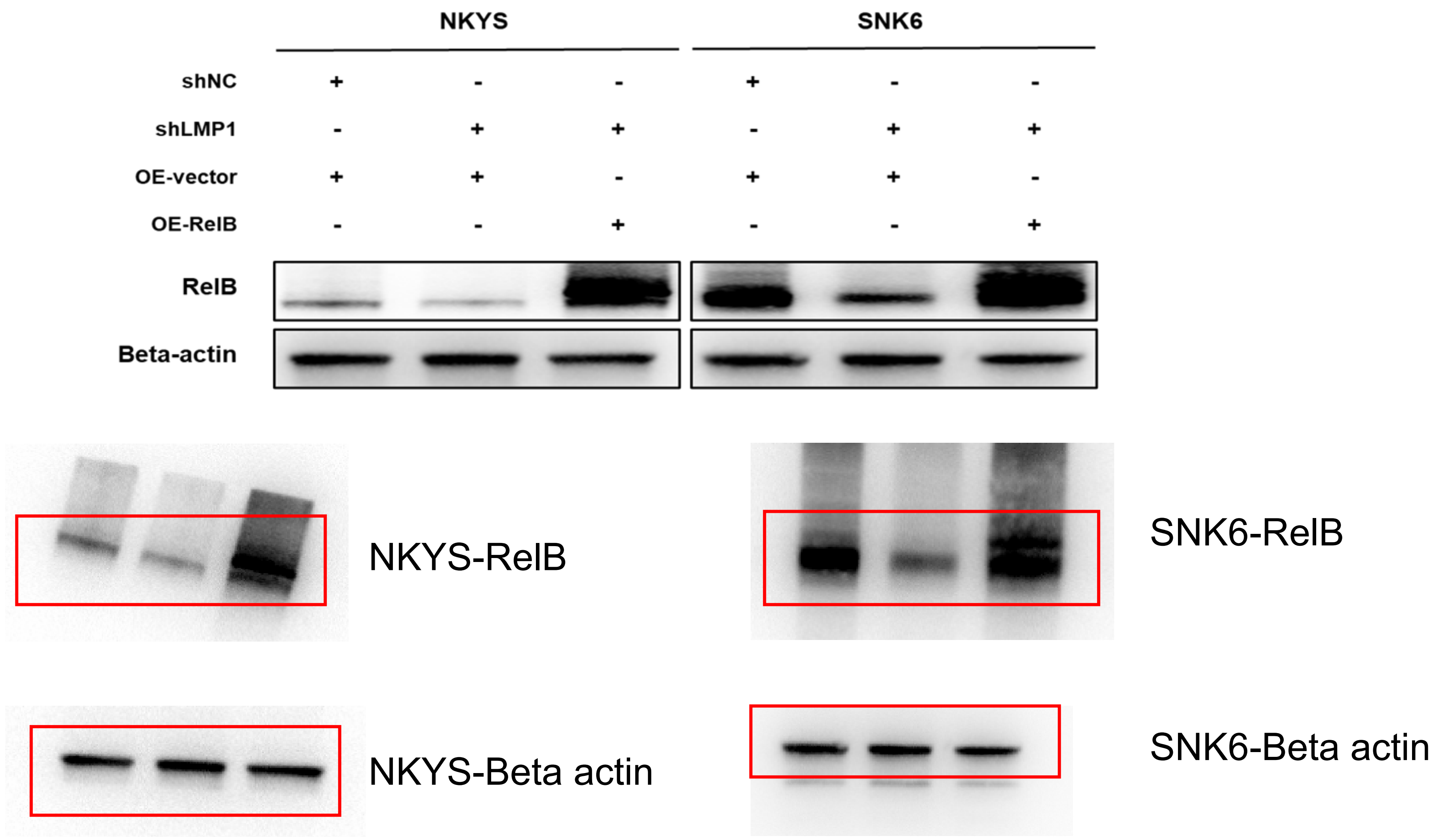

Supplement: Supplementary file 6 — Original Data File [file 41419_2024_6999_MOESM6_ESM.pdf]
